# Hb-EGF directs systemic muscle repair

**DOI:** 10.1242/dev.204993

**Published:** 2026-03-23

**Authors:** Haley C. Dean, Vishnu M. Saraswathy, Amulya Saini, Sibani Nachadalingam, Jennifer McAdow, Amruta Tendolkar, Mayssa H. Mokalled, Aaron N. Johnson

**Affiliations:** Department of Developmental Biology, Washington University in St. Louis, St Louis, MO 63108, USA

**Keywords:** Regenerative capacity, Muscle regeneration, Systemic muscle injury, Hb-EGF, Zebrafish

## Abstract

Regenerative capacity varies between tissues, species and stages of the life cycle. Regenerative capacity also varies with the magnitude of the injury, even within a single tissue. Vertebrate skeletal muscle fully regenerates in response to small-scale injuries, but large-scale injuries often lead to incomplete repair and debilitating loss of muscle use. To understand if small- and large-scale muscle injuries activate distinct regenerative programs, we developed a systemic muscle injury model in zebrafish. Transcriptomic analysis of muscle and non-muscle tissues revealed that systemic and local muscle injuries elicit distinct molecular responses, both quantitatively and qualitatively. We found that systemic muscle injury activates the expression of heparin-binding epidermal-like growth factor (Hb-EGF) in the epidermis and that Hb-EGF is necessary for systemic muscle repair. Conversely, local muscle injury did not induce Hb-EGF expression and Hb-EGF was not required for local muscle repair. These studies suggest that large- and small-scale muscle injuries activate different regenerative programs, resulting in either systemic or local repair.

## Teaser

**Summary:** Small- and large-scale muscle injuries activate distinct regenerative programs, and Hb-EGF signaling coordinates regeneration during systemic muscle repair.

## INTRODUCTION

Regenerative capacity varies significantly across species and developmental stages. Planaria and *Hydra* can regenerate entire body axes (Morgan, 1898, 1901; Gierer et al., 1972; Zammit, 2017); amphibians can regenerate entire limbs and organs (Fraisse, 1885; Harrison, 1921; Tanaka, 2003; Roddy et al., 2008). Regenerative capacity in mammals is, by comparison, limited to a few tissues, including skin, liver, bone and skeletal muscle. In addition, regenerative capacity is reduced with age. Fracture healing and muscle repair, for example, are slowed in the aging population, which contributes to a significant burden on the healthcare system and impacts the quality of life for older adults (Saul and Khosla, 2022; Grima-Terrén et al., 2024; Nguyen et al., 2025). While extensive comparative studies have revealed mechanisms that confer differential regenerative capacity, it remains unclear if the magnitude of the injury itself activates distinct regenerative programs in tissues and organisms with high regenerative capacity.

Transection injury models have provided some insights into the organismal response to injuries of different magnitudes. Planarians regenerate a new head after amputation, but do not duplicate the spared tissues or create additional body axes (Morgan, 1904a,b). This foundational observation argues that planarians have the capacity to recognize the extent of tissue loss and then elicit the appropriate regenerative response. Planarians repair large-scale injuries by activating a growth factor-mediated ‘missing tissue response’ that is thought to accelerate a single regenerative program, as opposed to activating a unique regenerative response (Tewari et al., 2018). The rate of regenerative growth in response to forelimb amputation in newts is also dependent on the magnitude of the injury. The regenerate produced after forelimb amputation proximal to the body grows faster than regenerates produced by distal amputations (Iten and Bryant, 1973). Similarly, the regeneration rate of zebrafish caudal fin rays following amputation correlates with the length of fin ray that is being replaced (Uemoto et al., 2020). Modulating the regeneration of individual rays reproduces a forked caudal fin when all rays are amputated along a single plane. These studies suggest the magnitude of tissue injury in highly regenerative organisms is sensed by a rheostat-like mechanism that governs the regeneration growth rate to appropriately compensate for the lost tissue.

Even though differential regeneration rates can compensate for the magnitude of tissue loss in certain contexts, some regenerative organs are limited in the volume of tissue that can be replaced following injury. The adult zebrafish heart, for example, can regenerate a ventricular resection, but only if less than 20% of the ventricular volume is lost (Poss et al., 2002). Similarly, neonatal mice can regenerate a ventricular amputation that removes up to 15% of the ventricular mass (Porrello et al., 2011). Skeletal muscle is also highly regenerative but, unlike heart regeneration, which depends on the dedifferentiation and proliferation of spared cardiomyocytes (Kikuchi et al., 2010), skeletal muscle repair depends on dedicated populations of muscle stem cells (MuSCs). Muscle injury activates MuSCs, which undergo asymmetric divisions to replenish the stem cell pool, as well as transient amplification and differentiation to generate myoblast progenitors dedicated to muscle repair (Troy et al., 2012; Pawlikowski et al., 2017; Feige et al., 2018). Mature myoblasts can fuse with and repair surviving myofibers, or fuse with each other to replace damaged myofibers with new myofibers (Demonbreun et al., 2015). MuSC-dependent regeneration can repair small-scale injuries, including mechanical ablation or toxin-based injuries that target a local region in fish or a single muscle in mammals (Seger et al., 2011; Gurevich et al., 2016; Hardy et al., 2016; Ratnayake et al., 2021). However, large-scale muscle loss in mammals that exceeds 20% of muscle mass results in incomplete muscle repair and often long-term disability (Sicherer et al., 2020). The striking contrast in regenerative capacity between amputated limbs and resected striated muscles argues that accelerating regenerative growth rates alone is not sufficient to repair all large-scale injuries.

One unexplored mechanism that could explain differential regenerative capacity in response to large-scale injuries is that independent regenerative programs direct organ repair in response to small- and large-scale injuries. We developed an inducible skeletal muscle injury model in highly regenerative zebrafish that damages all skeletal muscles simultaneously. Injured larvae showed a dramatic loss of motor function 2 days after systemic muscle injury and, incredibly, injured animals regained full motor function 10 days later. Local muscle injury and repair models have been established for zebrafish larvae (Knappe et al., 2015; Gurevich et al., 2016; Pipalia et al., 2016; Sharma et al., 2019; Ratnayake et al., 2021), and we used single-cell RNA sequencing (scSeq) of muscle and non-muscle tissues to compare the molecular responses to systemic and local muscle injuries. Our analysis identified quantitative differences in nearly 90% of the signaling pathways activated by systemic or local muscle injuries. In addition, systemic muscle injury activated the expression of heparin-binding epidermal like growth factor (Hb-EGF) and Hb-EGF is required for systemic repair. In contrast, local muscle injury did not induce Hb-EGF expression and Hb-EGF was dispensable for local repair. These studies support a model in which large- and small-scale muscle injuries activate distinct regenerative programs that direct systemic or local repair.

## RESULTS

### An inducible injury model to investigate systemic muscle repair

**Fig. 1.**
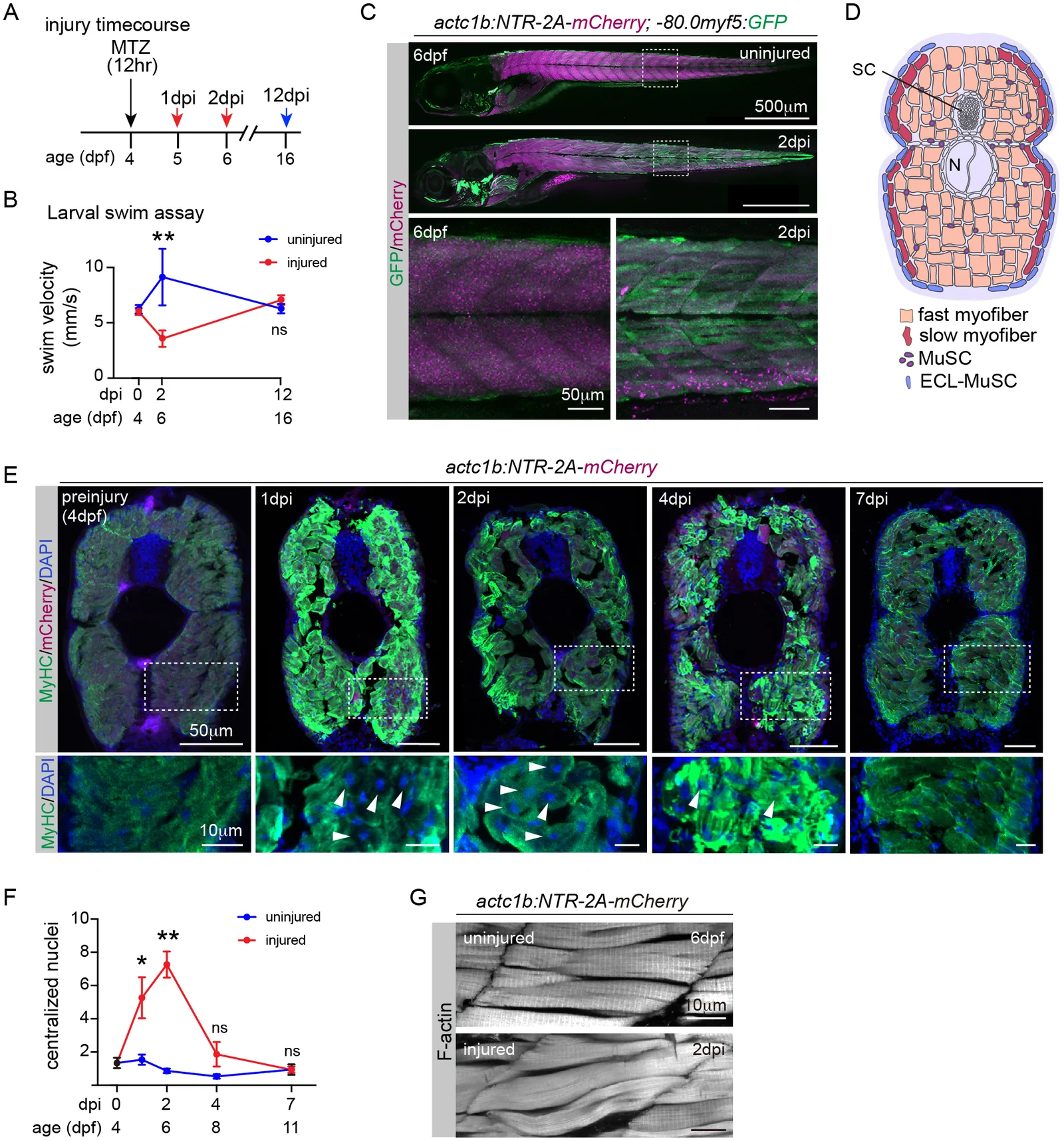
**An inducible model of systemic skeletal muscle injury.** (A) Experimental design to induce muscle injury and assess regeneration. (B) Larval swim function assay. The swim function of injured Tg(*actc1b*:NTR-2A-mCherry) larvae was drastically impaired at 2 dpi compared to uninjured controls, and was restored by 12 dpi. *n*≥19 per cohort. (C) Live images of Tg(*actc1b*:NTR-2A-mCherry) (*-80.0myf5*:GFP) double transgenic larvae. Uninjured larvae showed minimal *myf5*:GFP expression (green) in the trunk musculature. Larvae treated with MTZ for 12 h at 4 dpf showed distinct GFP fluorescence in myofibers at 2 dpi. The outlined areas are shown at higher magnification below. (D) Diagram of a transverse section through the larval trunk musculature, showing the cellular composition of the myotome. Dorsal is towards the top. SC, spinal cord; N, notochord. (E) Transverse sections of Tg(*actc1b*:NTR-2A-mCherry) larvae labelled for mCherry (red) and MyHC (green). At 1 dpi, intense MyHC signal from aggregates is present in myofibers throughout the trunk but resolves by 7 dpi. MyHC is enriched at the myofiber cortex at 7 dpi. High magnification panels below show centralized nuclei (arrowheads) at 1-, 2- and 4 dpi. Samples were prepared and imaged in parallel; systemic injury dramatically enhanced MyHC fluorescence intensity. (F) Quantification of centralized nuclei. Data points represent the average number of nuclei in three sections from five larvae. (G) Longitudinal sections of Tg(*actc1b*:NTR-2A-mCherry) larvae labelled for F-actin (phalloidin). Striated F-actin was absent from fast muscle fibers at 2 dpi. (B,F) *n*=5 fish per treatment group. Significance was determined using an unpaired Student's *t*-test. Data are mean±s.e.m. *P<0.05, **P<0.01.

To understand if systemic and local muscle injuries activate distinct regenerative programs, we developed an inducible systemic muscle injury model. The nitroreductase (NTR)-metronidazole (MTZ) system uses tissue-specific NTR expression to induce targeted injuries in zebrafish (Curado et al., 2008). NTR converts the pro-drug MTZ into a DNA crosslinker that causes cellular damage. We generated a stable zebrafish transgenic line in which the strong *actin alpha cardiac muscle1b* (*actc1b*) skeletal muscle promoter expresses NTR and mCherry in mature myofibers [hereafter Tg(*actc1b:*NTR-2A-mCherry)]. Tg(*actc1b:*NTR-2A-mCherry) larvae did not express mCherry in the heart, suggesting NTR-induced injury would be skeletal muscle specific. Larval muscles injured at 4 days post-fertilization (dpf) with a local needlestick injury showed efficient muscle repair by 6 days post injury (dpi). To identify a MTZ treatment that would induce a reparable muscle injury, we treated 4 dpf Tg(*actc1b:*NTR-2A-mCherry) larvae with a range of MTZ concentrations and incubation times, and assessed swim function at 2 and 12 dpi. Larvae exposed to 5 mM MTZ for 12 h showed decreased swim velocity at 2 dpi compared with controls (Fig. 1A,B). There was no difference in swim velocity at 12 dpi (Fig. 1B), arguing motor function was impaired and the larvae subsequently recovered. 5 mM MTZ treatment for 12 h was used for the remainder of our study to induce systemic muscle injuries.

Next, we evaluated the regenerative response to systemic muscle injury. Myf5 is expressed in activated MuSCs and bipolar myotubes in response to local muscle injury (Gurevich et al., 2016). Systemic injury of Tg(*actc1b:*NTR-2A-mCherry) (*myf5*:GFP) double transgenic larvae resulted in GFP-positive muscle fibers at 2 dpi (Fig. 1C), suggesting systemic and local regeneration use common pathways to repair damaged myofibers.

Histology on transverse sections of larvae after systemic injury showed myosin heavy chain (MyHC) aggregated at 1-, 2- and 4 dpi, which caused a dramatic increase in immunofluorescence (Fig. 1D,E). Regenerating myofibers in mammals can be identified by the presence of centralized myonuclei, and we found that the number of myofibers with centralized nuclei significantly increased at 1- and 2 dpi compared to uninjured controls (Fig. 1D,E). MyHC aggregates began to clear from myofibers at 4 dpi; by 7 dpi, MyHC was predominantly localized to the myofiber cortex (Fig. 1E).

Zebrafish trunk muscles comprise spatially distinct slow oxidative myofibers and fast glycolytic myofibers (Fig. 1D). *slow myosin heavy chain 1* (*smyhc1*) is expressed in the superficial slow myofibers and we generated a *smyhc1^sfgfp^* knock-in line to visualize slow myofibers in live animals. Uninjured *smyhc1^sfgfp^* larvae showed uniform GFP expression throughout the trunk, and the individual slow myofibers were highly organized and striated, indicating Smyhc1.GFP was incorporated into sarcomeres (Fig. S1A). In contrast, GFP accumulated at the somite boundaries in injured *smyhc1^sfgfp^* larvae at 2 dpi, and the myofibrils were highly disorganized and often lacked striations (Fig. S1A). We used longitudinal sections to visualize fast myofiber morphology and found injured fast fibers also lacked striations at 2 dpi (Fig. 1E). These data suggest that systemic injury impairs myofiber morphology and disrupts the contractile machinery in both slow and fast myofibers.

A 5 mM MTZ treatment for 12 h is not expected to cause toxicity in the absence of NTR or to cause a ‘bystander effect’ that injures neighboring cells (Sharrock et al., 2022). To further validate the specificity of our systemic muscle injury model, we assessed the morphology of the central nervous system, the vascular system and the heart in Tg(*actc1b:*NTR-2A-mCherry) larvae exposed to 5 mM MTZ for 12 h. We did not observe any obvious morphological defects in non-muscle tissues at 2 dpi (Fig. S1B-D), arguing NTR-MTZ based systemic muscle injury specifically affects skeletal muscle.

### Systemic muscle repair involves cell fusion

Systemic injury induced GFP fluorescence in Tg(*myf5*:GFP) larvae (Fig. 1C). To determine whether the GFP-positive myofibers arose from the fusion of GFP-expressing cells with injured myofibers, we live imaged Tg(*actc1b:*NTR-2A-mCherry) (*myf5*:GFP) larvae immediately after injury. Round GFP-positive cells localized to pre-existing myofibers and, over the imaging time course, GFP fluorescence from the round cells decreased until the cells were no longer visible, whereas GFP fluorescence in the proximal myofiber increased (Movie 1). These cellular events were similar to those reported for fusion of *pax7a*:GFP cells to pre-existing myofibers in response to needlestick injury (Pipalia et al., 2016), arguing that systemic muscle regeneration involves fusion-based myofiber repair.

To functionally test the requirement of MuSCs for systemic repair, we knocked an GFP-2A-NTR cassette into the *pax7a* locus to generate *pax7a^GFP-2A-NTR^* fish. *pax7a* is broadly expressed quiescent and activated MuSC populations (Pipalia et al., 2016), and *pax7a^GFP-2A-NTR^* larvae treated with MTZ are expected to have fewer Pax7a-expressing cells than control larvae. We treated transgenic larvae with MTZ and found that Tg(*actc1b:*NTR-2A-mCherry) *pax7a^GFP-2A-NTR^* larvae had significantly reduced viability compared to Tg(*actc1b:*NTR-2A-mCherry) or *pax7a^GFP-2A-NTR^* larvae (Fig. S1E,F). Importantly, *pax7a^GFP-2A-NTR^* larvae treated with MTZ did not show a significant change in viability compared to uninjured controls (Fig. S1F). Our live imaging and *pax7a^GFP-2A-NTR^* studies argue that systemic muscle repair is a fusion-based process that requires *pax7a*-expressing cells.

### Myofibers survive systemic muscle injury

Myf5-expressing cells fused to pre-existing myofibers, suggesting myofibers survive systemic injury and are then repaired. We labeled fast and slow myofibers for the apoptosis marker cleaved caspase 3 and found systemic injury induced cleaved caspase 3 expression in just a few myofibers (Fig. S2A). By comparison, local needlestick injury induced cleaved caspase 3 expression in most of the injured myofibers (Fig. S2A). Although slow muscles did not express appreciable levels of cleaved caspase 3 in response to systemic injury, the number of GFP-positive cells was significantly reduced in transverse sections of *smyhc1^sfgfp^* larvae at 2 and 4 days after systemic injury compared to uninjured controls (Fig. S2A-C). This change in GFP expression likely arises from the disorganization of slow myofibers with mislocalized Smyhc1-GFP (Fig. S1A). A majority of slow and fast myofibers appeared to survive systemic injury.

**Fig. 2.**
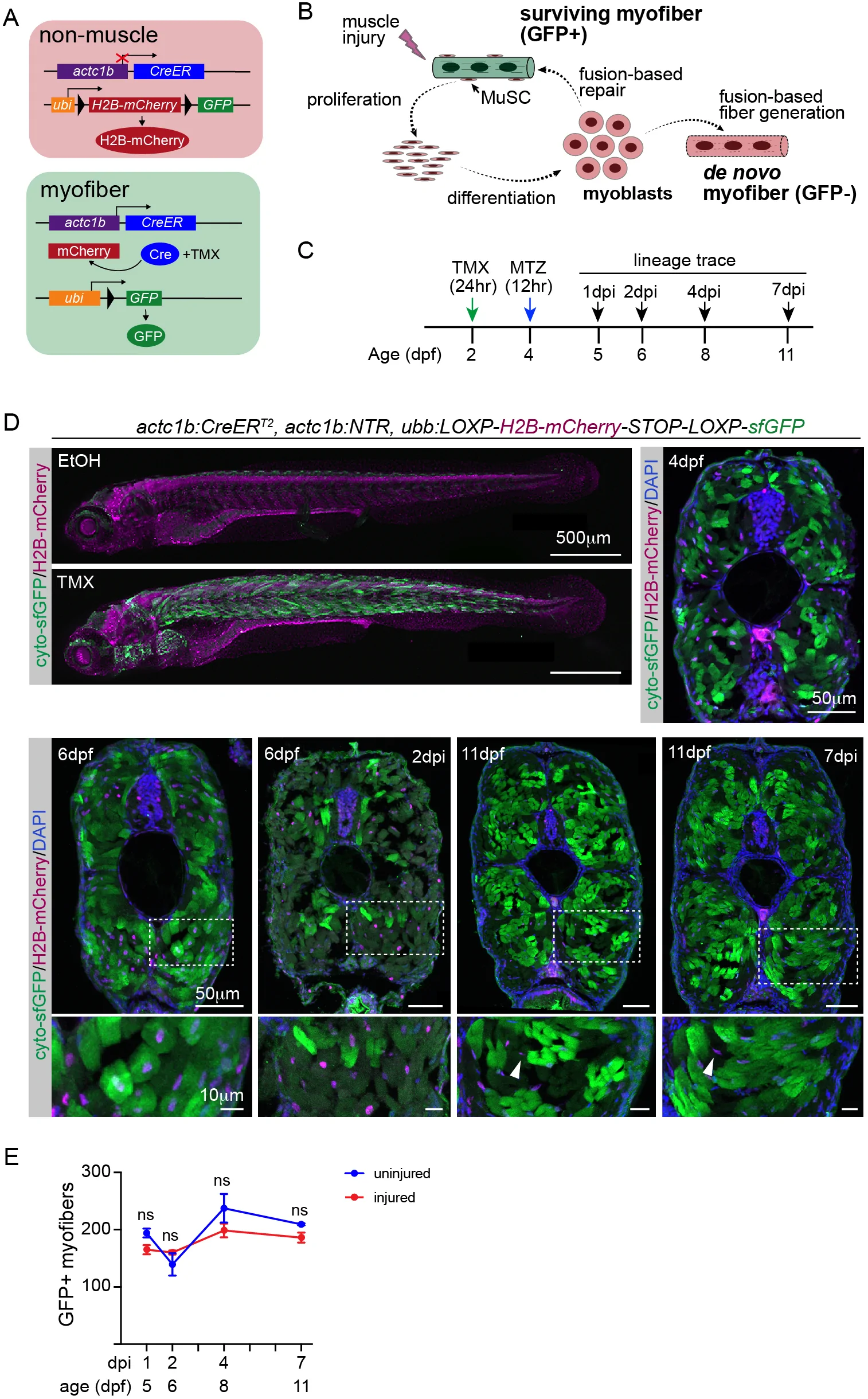
**Injured myofibers survive systemic injury.** (A) Lineage-tracing strategy. In the presence of tamoxifen (TMX), *actc1b*:CreER^T2^-derived CreER^T2^ induces recombination of the target cassette, which activates GFP expression in myofibers. Non-muscle cells do not express CreER^T2^ and should not express GFP. (B) Myofiber lineage trace during muscle regeneration. Myofibers that survive muscle injury are GFP positive, while newly generated myofibers are GFP negative. (C) Experimental strategy to lineage trace myofibers. (D) Larvae that were triple transgenic for Tg (*actc1b*:NTR) (*actc1b*:CreER^T2^) (*ubb:LOXP-H2B-mCherry-STOP-LOXP-SFGFP*) were lineage traced in the presence and absence of MTZ-induced injury. Lateral views of whole-mount larvae show GFP-positive myofibers after 24 h of TMX treatment. Vehicle-treated larvae (ethanol) did not activate GFP expression. H2B-mCherry protein was highly stable and continued to fluoresce after recombination. Transverse sections show myofiber lineage labelling of pre-injured (4 dpf), control (6 dpf and 11 dpf) and MTZ-injured (2 dpi and 7 dpi) larvae. Injured and control fish had a comparable number of GFP-positive myofibers in the trunk musculature, although some GFP-negative myofibers were present on both treatment groups (arrowheads). The outlined areas are shown at higher magnification below. (E) Quantification of GFP-positive myofibers from lineage-traced larvae. The number of GFP-positive myofibers was not significantly different between uninjured and injured larvae at any time point. Significance was determined using an unpaired Student's *t*-test. Data points represent the average number of GFP-positive myofibers±s.e.m. in three sections from ten larvae. ns, not significant; cyto, cytoplasmic.

*smyhc1* is expressed in newly created fast myofibers after local muscle injury (Gurevich et al., 2016), and larvae from our *smyhc1^sfgfp^* knock-in line showed GFP-positive myofibers in the fast myotome 2 days after local injury (Fig. S2D). In contrast, systemic injury did not induce GFP-positive myofibers in the fast myotome of *smyhc1^sfgfp^* larvae at 2 dpi, arguing that systemic repair does not generate new myofibers (Fig. S2B). Since it was possible that systemic repair generates new myofibers through a Smyhc1-independent mechanism, we generated Tg(*actc1b:*CreER^T2^) transgenic fish to lineage trace myofibers using the reporter Tg(*ubb:LOXP-H2B-mCherry-STOP-LOXP-GFP*) (Fig. 2A). Myofibers that survive systemic injury are predicted to remain GFP positive over the course of the lineage trace, while myofibers that are replaced with new myofibers are predicted to be GFP negative (Fig. 2B). Larvae that were triple transgenic for Tg(*actc1b:*NTR), Tg(*actc1b:*CreER^T2^) and the lineage trace reporter Tg(*ubb:LOXP-H2B-mCherry-STOP-LOXP-GFP*) were treated with tamoxifen (TMX) at 2 dpf for 24 h, injured with MTZ for 12 h, and either directly live imaged for GFP or collected at 1-, 2-, 4- and 7 dpi for histology (Fig. 2C). Triple transgenic larvae showed strong recombination of the lineage trace reporter in myofibers 24 h hours after TMX treatment, arguing the lineage trace system strongly labeled mature myofibers prior to injury (Fig. 2D). Live imaging of TMX-treated, MTZ-injured larvae revealed injured myofibers retained GFP expression for 24 h but did not show evidence of myofiber death or clearance (Fig. S2E). In addition, histological analysis showed the number of GFP-positive myofibers at 1-, 2-, 4- and 7 dpi were comparable between injured and uninjured larvae (Fig. 2D,E). Our lineage-tracing and Smyhc1-GFP expression studies further argue that the majority of myofibers survive systemic injury.

### The cellular response to systemic and local injuries

**Fig. 3.**
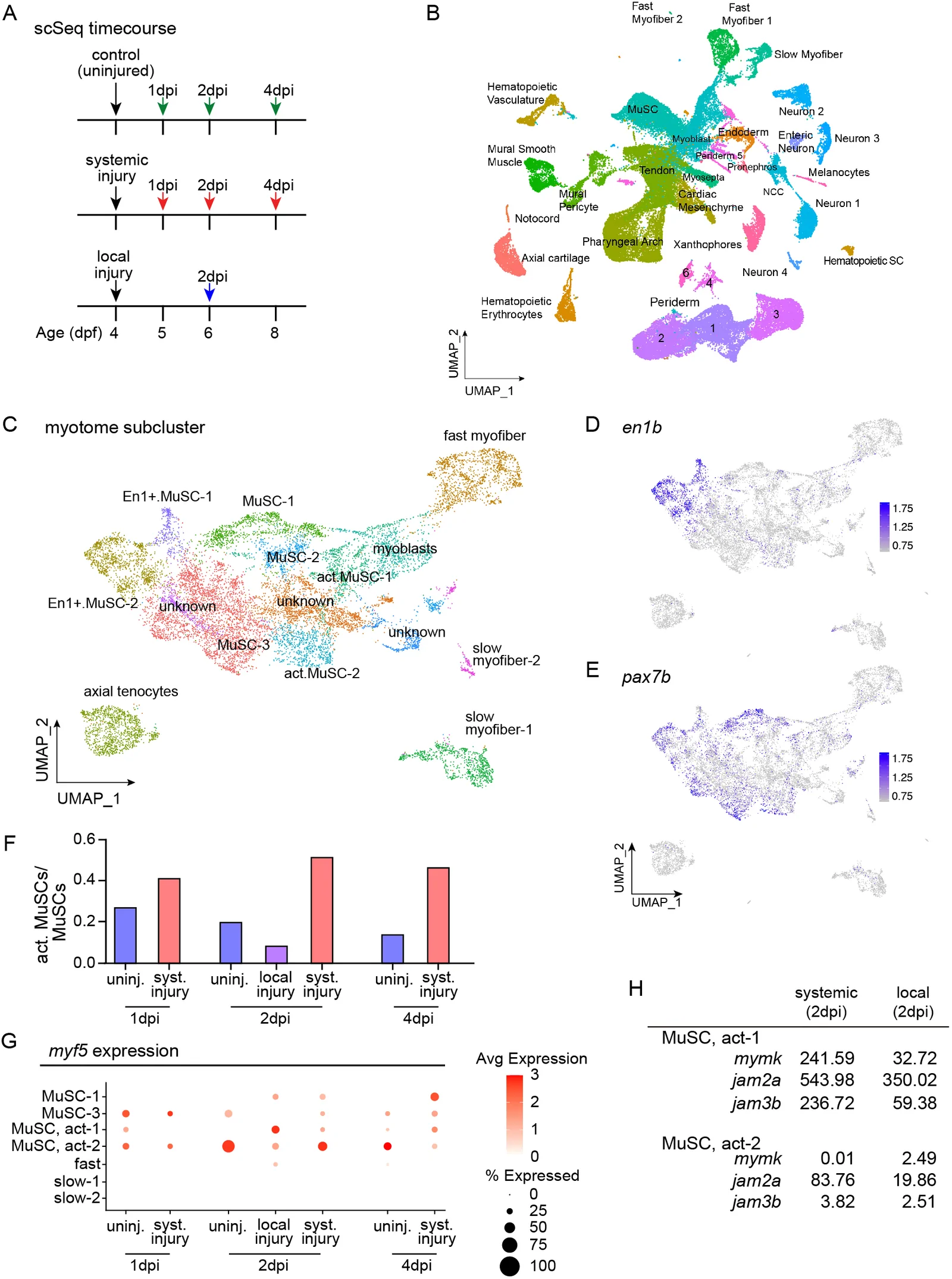
**scSeq of larvae with systemic and local muscle injuries.** (A) Experimental design for single-cell sequencing (scSeq). Seven cohorts were collected for scSeq. (B) Merged UMAP representation of the complete data set, representing 67,554 cells. A total of 32 clusters were identified. (C) UMAP plot showing 15 muscle subclusters, including multiple population of MuSCs. The precise identities of three clusters remain unknown. (D,E) Feature plots showing the distributions of canonical MuSC markers *en1b* (D) and *pax7b* (E) among the muscle subclusters. (F) Ratio of activated MuSCs to MuSCs during muscle repair. Cell proportions were normalized as the total number of cells in each sample. Systemic injury produced more activated MuSCs than local injury. (G) Dot plot of *myf5* expression in the muscle subcluster dataset is restricted to the MuSC clusters shown. *myf5* was not detected in mature myofibers. Dot colors and diameters represent average gene expression and the percentage of cells expressing a given marker throughout the manuscript. (H) Normalized expression of *mymk, jam2a* and *jam3b* transcripts in activated MuSCs. mRNA expression was similar between injury models. Paralogs with the highest expression are shown.

We hypothesized that systemic and local muscle injuries activate distinct regenerative programs. To evaluate the cellular and molecular responses to systemic and local injuries, we performed scSeq of uninjured and MTZ-injured larvae at 1-, 2- and 4-dpi, and scSeq of local needlestick-injured larvae at 2 dpi (Fig. 3A). Cell suspensions of dissociated trunks from multiple individuals were pooled for each cohort, and isolated cells were sequenced using 10X genomics platform (3′ v3.1 chemistry). Sequence reads were aligned to zebrafish genome GRCz11, and cells were filtered using the Decontx and DoubletFinder packages to exclude cells that include doublets or high quantities of ambient and mitochondrial RNA (see Materials and Methods for a complete description of scSeq bioinformatic analyses). A total of 67,554 cells were used for subsequent analyses.

Unsupervised clustering identified 32 cell populations in the larval trunk (Fig. 3B). We used the differentially expressed genes (DEGs) among cell populations from a zebrafish embryo and larva scSeq atlas (Sur et al., 2023) to generate a matrix that assigned the 32 clusters to one of 14 tissues (Fig. S3A, Table S1). Six of the clusters were assigned to multiple tissues, which we resolved by comparing only the identifier genes from the scSeq atlas to each tissue type (Fig. S3B). Clusters were then assigned subidentities using cell type-specific markers from the scSeq atlas (Fig. 3B). In all, five clusters of muscle cells were identified (MuSCs, myoblasts, slow myofibers and two clusters of fast myofibers; Fig. S3C), along with other cell types expected to be present in the larval trunk (cartilage, tendons, neurons, periderm, vasculature and monocytes). Subclustering the muscle cell populations uncovered seven MuSC clusters (Fig. 3C), which showed differential expression of the MuSC markers *en1b* and *pax7b* (Fig. 3D,E). The muscle subclusters also contained differentiating muscle progenitors that expressed *myod* and *myog*, and terminally differentiated myofibers that expressed *actc1b* (Fig. S3D). To contextualize the zebrafish muscle subclusters, we performed interspecies comparisons with single-cell and single-nucleus studies of regenerating mouse muscles (McKellar et al., 2021). Our matrix analysis identified two populations of zebrafish MuSCs that were similar to activated MuSCs in mice, which we could then use quantify MuSC dynamics during zebrafish systemic repair. The ratio of activated MuSCs to MuSCs was higher in larvae at 1-, 2- and 4 dpi after systemic injury than in uninjured larvae or in larvae with local injuries, arguing systemic injury activates more MuSCs to complete a large-scale repair than local injury (Fig. 3F). In addition, MuSCs and activated MuSCs expressed *myf5*, but myofibers did not (Fig. 3G), supporting our hypothesis that GFP fluorescence in myofibers after the systemic injury of Tg(*myf5*:GFP) fish is the result of fusion with GFP-positive myoblasts and not of the transcription of *myf5*:GFP in damaged myofibers. Activated MuSCs in larvae with systemic and local injuries also expressed similar levels of the fusogen-encoding transcripts *myomaker* (*mymk*) and *junctional adhesion molecule 2a* (*jam2a*) (Fig. 3H). These data suggest that systemic injuries activate more MuSCs than local injuries but, once activated, MuSCs initiate a similar regenerative response to direct systemic and local repair.

### Systemic and local injuries activate distinct signaling pathways

**Fig. 4.**
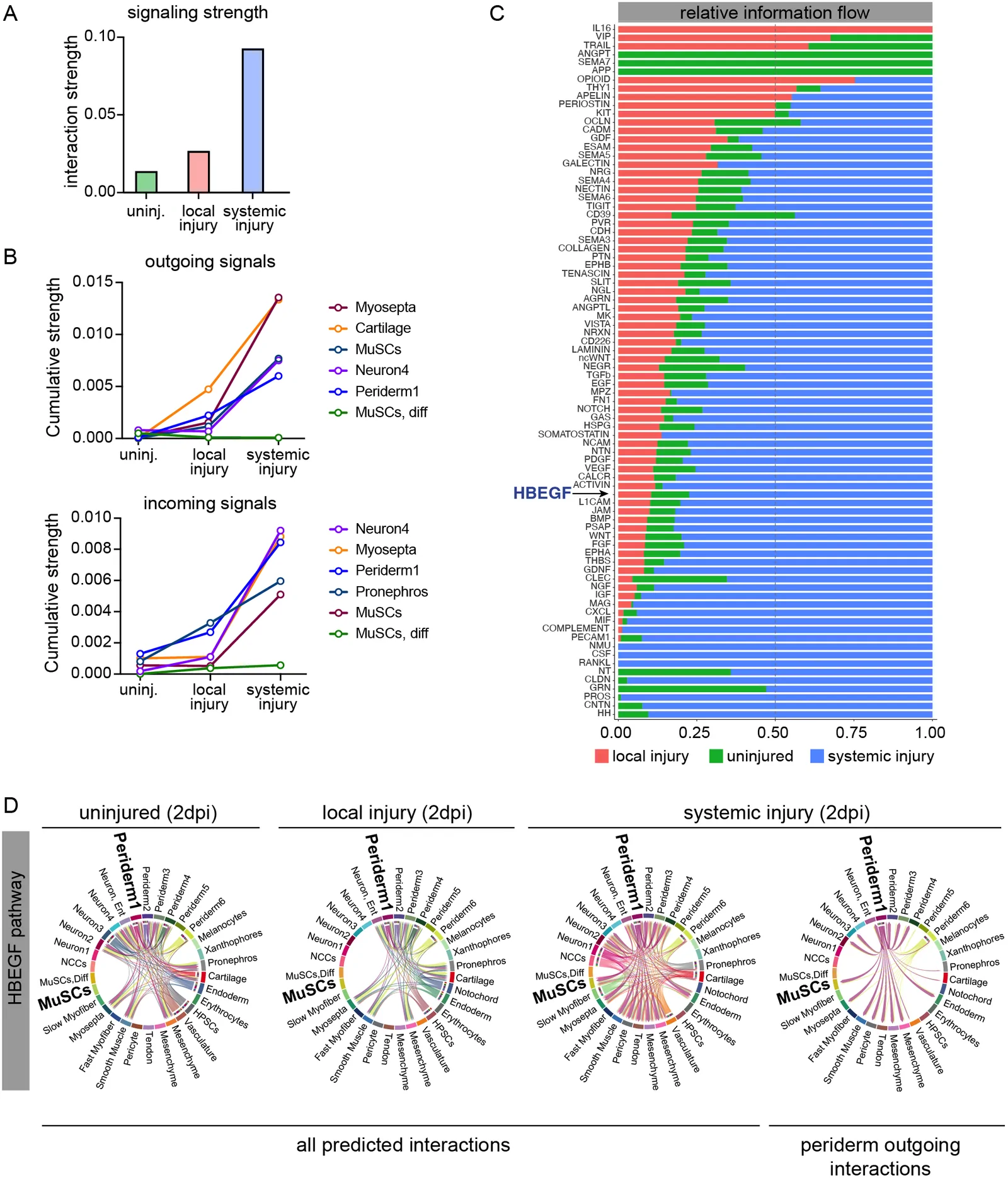
**Systemic injury activates unique cell-cell communication networks.** (A) Relative interaction strengths in uninjured (uninj.), locally injured and systemically injured samples at 2 dpi. (B) Cumulative strengths of signaling pathways outgoing from or received by the five strongest cell types at 2 dpi. The ‘MuSC, diff’ (differentiated MuSCs) population is shown as a low-strength outlier. (C) Information flow plot showing relative signaling flow among the three samples at 2 dpi. Most signaling pathways showed greater information flow in response to systemic injury, including heparin-binding epidermal-like growth factor (Hb-EGF). (D) Chord diagrams showing Hb-EGF intercellular communication in response to injury at 2 dpi. Only systemic injury induces Hb-EGF signaling to MuSCs. A chord diagram for periderm 1 outgoing only (far right) shows predicted Hb-EGF communication with other cell types, including MuSCs.

To understand if systemic and local injuries induce distinct signaling responses, we used the CellChat program to quantify intercellular communication networks based on the expression of ligands, receptors and pathway modifiers (Jin et al., 2021, 2025). Zebrafish genes were first converted to human orthologues, as we previously described (Saraswathy et al., 2024), and then entered into the CellChat platform to calculate the communication probabilities of individual signal transduction pathways in outgoing (signaling) and incoming (signal receiving) cells. The cumulative probabilities of outgoing and incoming signals were integrated across all pathways, which revealed the cumulative interaction strength was higher in response to systemic injury than local injury (Fig. 4A). The cumulative outgoing and incoming signaling strength was also higher in response to systemic injury (Fig. 4B). Cumulative signaling strength can also be calculated for individual clusters. Four clusters (periderm1, MuSCs, neuron4 and myosepta) showed dramatically higher incoming and outgoing signaling strength in response to systemic injury than local injury (Fig. S4A).

Next, we compared the information flow for each signaling pathway between uninjured, local and systemic injuries (Fig. 4C). Information flow is the sum of communication probabilities across clusters and conditions for an individual pathway. We found that several signaling pathways showed information flow exclusively under uninjured (ANGPT, SEMA7 and APP), locally injured (IL16) or systemically injured (CSF, NMU and RANKL) conditions, but a majority of the signaling pathways showed dynamic information flow across conditions (Fig. 4C). In addition, information flow for 62 of the remaining 74 signaling pathways was higher in systemic injury than in local injury or in uninjured samples. These analyses suggest systemic and local injuries induce distinct signaling responses that activate qualitatively different regenerative programs.

### Systemic injuries activate signaling pathways outside the MuSC niche

To identify potential signaling pathways that control unique systemic repair programs, we looked for pathways with high information flow in systemic injury that either signaled to MuSCs only in response to systemic injury or that signaled from MuSCs only in response to systemic injury. Three signaling pathways met these criteria including the epidermal growth factor (EGF), macrophage migration inhibitory factor (MIF) and nerve growth factor (NGF) pathways. EGF and MIF are MuSC incoming signals; NGF is a MuSC outgoing signal (Fig. S4B). To understand which EGF ligand might be signaling to MuSCs after systemic injury, we repeated the CellChat analysis using individual ligands and found that heparin binding epidermal growth factor (Hb-EGF) signaling recapitulated the outputs observed using all EGF ligands (Fig. 4D, Fig. S4B). Periderm 1 and MuSCs had some of the highest cumulative interaction strengths among the clusters (Fig. S4A), and Hb-EGF showed the strongest signaling from periderm 1 to MuSCs (Fig. 4D). Hb-EGF was thus a strong candidate for a specific signaling response that is activated by systemic, but not local, injury.

**Fig. 5.**
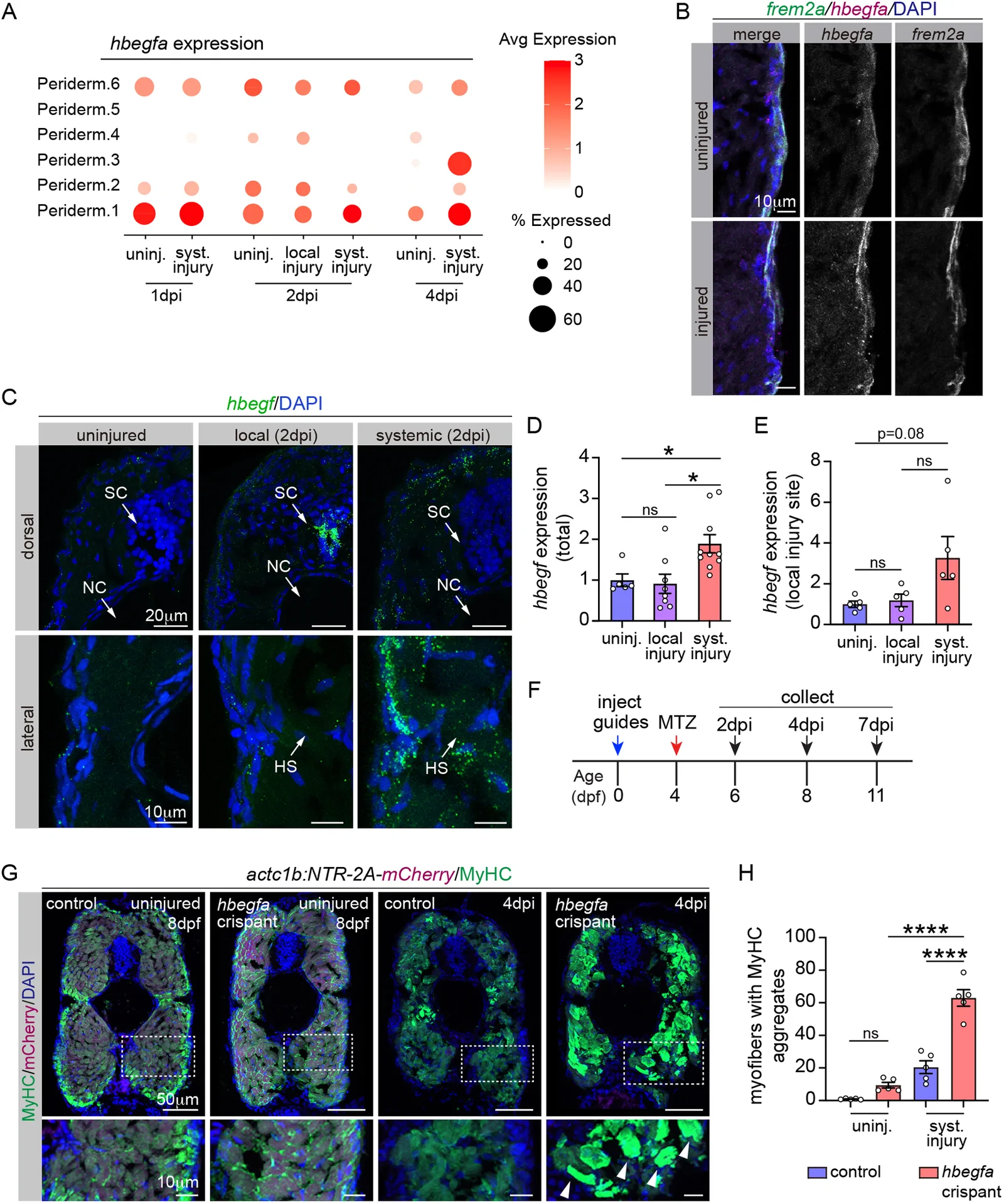
**Systemic repair requires *hbegfa*.** (A) Dot plot of *hbegfa* expression in the periderm from the complete data set. *hbegfa* was enriched in periderm.1 and periderm.3 after systemic injury. (B) *hbegfa*/*frem2a* double HCR in larvae transgenic for Tg(*actc1b*:NTR-2A-mCherry). Systemic injury enhanced *hbegfa* expression in cells that expressed the periderm marker gene *frem2a* at 2 dpi. Representative images from *n*=5 larvae per treatment group. (C) *hbegfa* HCR in Tg(*actc1b*:NTR-2A-mCherry) larvae at 2 dpi. *hbegfa* expression was induced in response to systemic, but not local, injury. (D,E) Quantification of *hbegfa* expression over entire transverse sections (D) or at the site of needlestick injury (E). (F) Experimental design for CRISPR-mediated deletion of *hbegfa*. Tg(*actc1b*:NTR-2A-mCherry) embryos were injected at the one-cell stage, injured at 4 dpf, and collected for histology. (G) Transverse sections of control and *hbegfa* crispant Tg(*actc1b*:NTR-2A-mCherry) larvae without injury (8 dpf) or at 4 dpi, labelled for MyHC (green) and mCherry (red). Uninjected siblings were used as controls. Areas outlined are shown at higher magnification below. Myofibers with intense MyHC expression are mostly cleared by 4 dpi in controls. Injured *hbegfa* crispants failed to clear MyHC aggregates (arrowheads). MyHC expression was similar between uninjured *hbegfa* crispants and controls. (H) Quantification of myofibers with MyHC aggregates at 4 dpi. Data points in D, E and H show average values±s.e.m. in three sections from an individual larva. Significance was determined using an unpaired *t*-test. ns, not significant. *P<0.05, ****P<0.0001. HS, horizontal septa; NC, notochord; SC, spinal cord.

We developed a high-throughput pipeline to validate candidates identified by scSeq analysis in zebrafish that uses hybridization chain reaction (HCR)-fluorescent *in situ* hybridization (HCR-FISH) to visualize candidate transcript expression in response to injury, and then assesses the functional requirement of the associated gene for regeneration using ribonucleotide (RNP)-mediated CRISPR genome editing (Saraswathy et al., 2024). The advantage of this validation pipeline is that the RNP-CRISPR mutagenesis is efficient enough to assess loss of gene function in F_0_ animals. The zebrafish genome encodes two *hbegfa* transcripts, *hbegfa* and *hbegfb*, but scSeq analysis showed only *hbegfa* was induced in the periderm at 1-, 2- and 4-days after systemic injury (Fig. 5A). HCR-FISH of *hbegfa* and a periderm specific marker, *FRAS1 related extracellular matrix 2a* (*frem2a*; Fig. S5A), showed that *hbegfa* is induced in the periderm by systemic injury (Fig. 5B,D). We also compared *hbegfa* expression in response to systemic and local injury. Local injury was consistently performed at the same position along the rostral-caudal axis. We quantified *hbegfa* expression at the local injury site and at an analogous position in larvae recovering from systemic injury, and found that *hbegfa* expression was not induced by local injury (Fig. 5C,E). These expression studies argue *hbegfa* is induced in the periderm in response to systemic, but not local, injury.

### *hbegfa* is required for systemic repair

To determine whether Hb-EGF is required for systemic repair, we used RNP CRISPR to delete the entire *hbegfa*-coding region. We injured F_0_ *hbegfa* ‘crispants’ double transgenic for Tg(*actc1b:*NTR-2A-mCherry) and *smyhc1^sfgfp^* at 4 dpf, and found slow myofiber regeneration was significantly disrupted in *hbegfa* crispants, resulting in missing and morphologically aberrant myofibers (Fig. S5B,C). Fast myofiber regeneration was also impaired in *hbegfa* crispants, which failed to clear MyHC aggregates at 4 dpi (Fig. 5F-H). Uninjured *hbegfa* crispants showed normal muscle morphology at 8 dpf (Fig. 5G), arguing the muscle phenotypes in injured *hbegfa* crispants are due to impaired regeneration rather than to developmental abnormalities. Hb-EGF is thus required for systemic repair of fast and slow myofibers.

Our expression studies showed Hb-EGF expression is induced by systemic injury but not by local injury, suggesting Hb-EGF is dispensable for local muscle repair. We performed local injuries on *hbegfa* crispants and found slow myofiber regeneration was comparable between *hbegfa* crispants and injured controls at 7 dpi (Fig. S5B,C). These studies are consistent with a model in which Hb-EGF activates a systemic muscle repair program that is distinct from the local muscle repair program.

### Hb-EGF promotes muscle stem cell expansion

We identified seven larval MuSC populations by scSeq, which we grouped as En1-positive MuSCs (En1+.MuSC-1,2), En1-negative MuSCs (MuSC-1,2,3) and activated MuSCs (act.MuSC-1,2). *en1b* is expressed in En1-positive MuSCs and in a subset of activated MuSCs; *pax7b* is expressed in all seven MuSC populations but not in every cell (Fig. 3D,E). By histology, En1 is expressed in spatially restricted MuSCs, known as the external cell layer (ECL), which are located superficial to slow myofibers (Keenan and Currie, 2019). We labelled Tg(*en1b-hs:*Gal4), (*UAS*:RFP), *smyhc1^sfgfp^* double transgenic larvae with a Pax7 antibody, and confirmed En1-positive cells in the ECL are immediately adjacent to slow fibers (Fig. S6A). This analysis further validated our scSeq, which showed only a subset of En1-positive cells express Pax7 (Fig. 3D,E; Fig. S6A). In addition, the En1 antibody labeled more ECL cells than the Tg(*en1b-hs:*Gal4) (*UAS*:RFP) transgenes (Fig. S6B), arguing that the ECL MuSCs are heterogenous and either express different combinations of *en1a* and *en1b*, or that *en1b* expression is regulated by multiple enhancers.

**Fig. 6.**
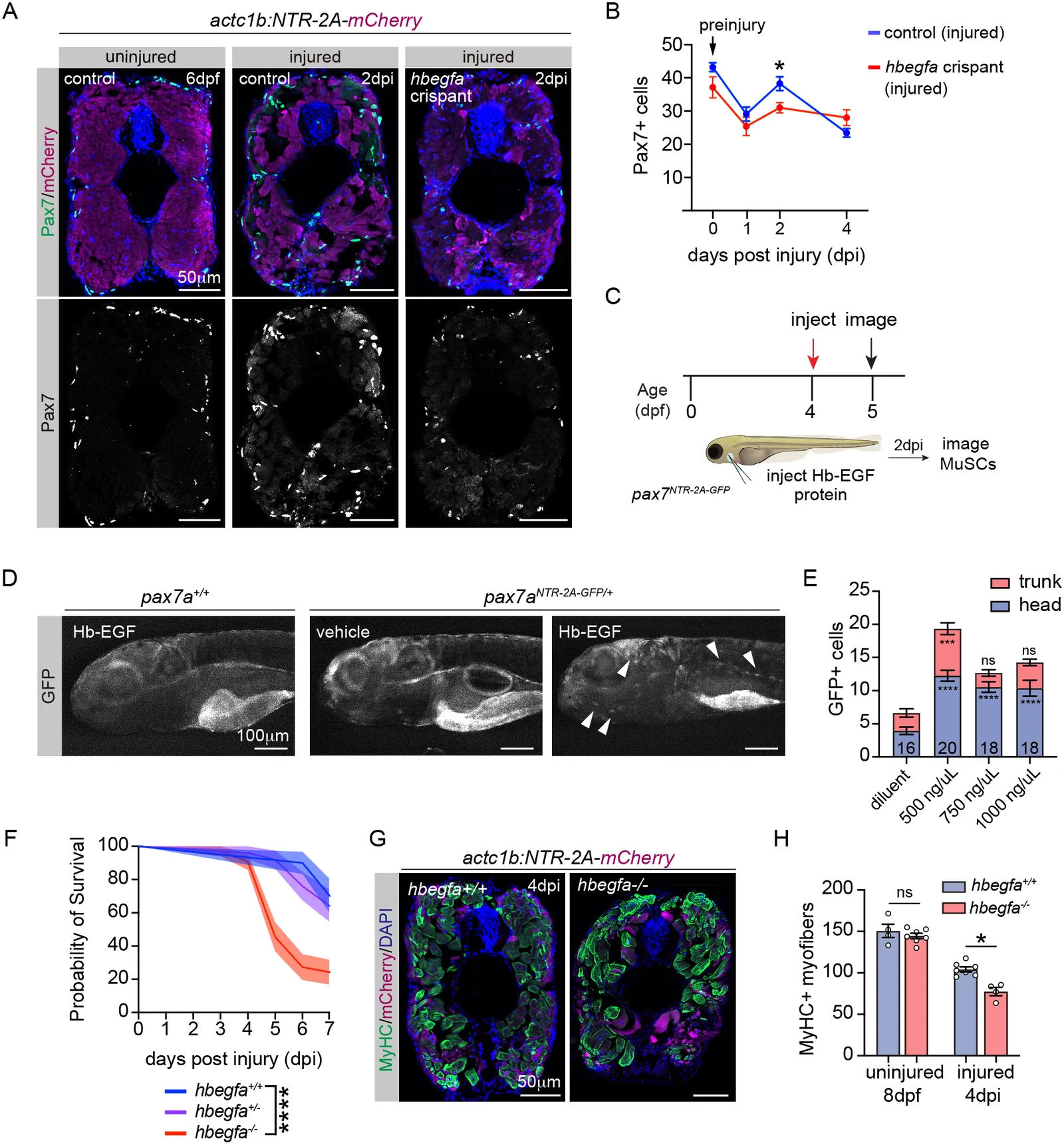
**Hb-EGF regulates the MuSC response to systemic injury.** (A) Transverse sections of Tg(*actc1b*:NTR-2A-mCherry) larvae at 6 dpf (uninjured) or 2 dpi (injured), labelled for Pax7 (green) and mCherry (red). *hbegfa* crispants were injected at the one-cell stage; uninjected siblings were used as controls. (B) Quantification of Pax7-positive cells from the analysis shown in A. The number of Pax7-positive cells was significantly lower in *hbegfa* crispants at 2 dpi. Control and *hbegfa* crispant larvae showed comparable numbers of Pax7-positive cells prior to injury. Data are mean±s.e.m. (*n*≥5 per group per time point). (C) Experimental design for systemic injection of human recombinant Hb-EGF. *pax7a^GFP-2A-NTR^* larvae were injected through the duct of Cuvier at 4 dpf and live imaged at 48hpi. (D) Live images of *pax7a^GFP-2A-NTR^*-injected larvae show GFP-positive cells in the head and trunk of Hb-EGF-injected larvae (arrowheads). PBS-injected siblings were used as controls. (E) Quantification of Pax7-positive (GFP-positive) cells in live injected larvae. Hb-EGF protein concentrations are given for each injection. The numbers of larvae quantified are shown. Data are mean±s.e.m. (F) Survival curve. Tg(*actc1b*:NTR-2A-mCherry) larvae were treated with MTZ. *hbegfa^−/−^* larvae showed significantly reduced survival compared to sibling controls. *n*≥40 per genotype. (G) Transverse sections of MTZ-treated Tg(*actc1b*:NTR-2A-mCherry) larvae at 4 dpi, labelled for MyHC (green) and mCherry (red). (H) Quantification of MyHC-positive myofibers. *hbegfa^−/−^* larvae had significantly fewer myofibers than sibling controls. Data points represent average myofiber number±s.e.m. in three transverse sections from a single larva. Significance was determined using an unpaired Student's *t*-test (B,E,H) and Log-rank test (F). ns, not significant. *P<0.01, ****P<0.0001.

To understand if Hb-EGF regulates MuSC dynamics during systemic repair, we used the En1 and Pax7 antibodies to broadly label and quantify MuSCs. *hbegfa* crispants and sibling controls showed a similar number of Pax7-positive cells prior to injury. One day after MTZ treatment, both groups showed a comparable decrease in the number of Pax7-positive cells but at 2 dpi control larvae showed significantly more Pax7-positive cells than *hbegfa* crispants (Fig. 6A,B). In contrast, the number of En1-positive cells was comparable between control larvae and *hbegfa* crispants at 1 and 2 dpi (Fig. S6B,C). Hb-EGF is therefore required for Pax7-positive MuSCs to expand during systemic muscle repair. Next, we injected human recombinant Hb-EGF protein into the systemic circulation of *pax7a^GFP-2A-NTR^* larvae to functionally assess the role of Hb-EGF in MuSC expansion. Larvae were injected with three doses of Hb-EGF, and each treatment induced a significant increase in the number of GFP-positive cells 48 h post-injection (Fig. 6C-E, Fig. S6D). These data argue that Hb-EGF treatment is sufficient to induce MuSC expansion.

### *hbegfa* mutants are defective for systemic repair

We recovered germ line-transmitted *hbegfa* mutations that deleted the entire *hbegfa*-coding region. *hbegfa^−/−^* larvae transgenic for Tg(*actc1b:*NTR-2A-mCherry) had reduced viability after systemic injury compared to wild-type and heterozygous siblings, with a sharp decline in survival starting at 4 dpi (Fig. 6F). Histological analysis showed *hbegfa^+/+^* and *hbegfa^−/−^* larvae had a comparable number of myofibers prior to injury, but *hbegfa^−/−^* larvae had significantly fewer myofibers at 4 dpi (Fig. 6G,H). To determine whether *hbegfa* is required for local muscle repair, we performed local needlestick injury on 4 dpf larvae and found the number of myofibers in the injured region was comparable between *hbegfa^−/−^* larvae and wild-type siblings at 7 dpi (Fig. S6E,F). Hb-EGF is therefore required for systemic, but not local, muscle repair.

## DISCUSSION

Our model of inducible systemic muscle injury revealed that systemic and local injuries induce distinct molecular responses to direct muscle repair. Systemic muscle injury resulted in myofiber damage, which was predominantly repaired, but not replaced, with new myofibers. Transcriptomic analysis showed intercellular signaling strength was higher during systemic muscle repair relative to local repair. This differential signaling strength may reflect the need to orchestrate an organism-wide response to repair muscle that involves signaling across tissues. Hb-EGF expression was significantly enriched outside the myotome after systemic injury and was required for systemic repair. In addition, systemic delivery of Hb-EGF activated MuSC proliferation, arguing Hb-EGF can act over long distances to promote muscle repair. These studies argue multiple regenerative programs are in place to ensure robust regeneration of injured skeletal muscle.

The systemic environment plays an important yet relatively unknown role in regulating MuSC function. Heterochronic parabiotic experiments, in which the circulatory systems of young and aged mice are mixed, revealed an incredible capacity for the young circulatory system to enhance MuSC proliferation and to reduce fibrogenic differentiation in aged mice (Hong et al., 2022). Conversely, the circulatory system from old mice increased fibrosis in young mice. These foundational studies revealed that the systemic environment provides an additional layer of regulation beyond the stem cell niche to control MuSC function. Although the circulating factors that direct age-related changes in MuSC function remain controversial (Hong et al., 2022), these studies demonstrate that MuSC activity can be remotely controlled over relatively long distances.

An essential clue that muscle injury induces an organism-wide response at the cellular level appeared in studies in which mammalian skeletal muscle was damaged in one limb, and MuSC activity was assessed in the uninjured, contralateral limb. Surprisingly, quiescent MuSCs in the contralateral limb transitioned into an ‘alert’ state after injury, and were poised to activate and repair tissue damage quickly (Rodgers et al., 2014). At the molecular level, hepatocyte growth factor activator (HGFA) is induced by muscle injury and activates human growth factor (HGF). HGF, in turn, acts at the stem cell niche, where it directs MuSCs to transition into the alert state (Rodgers et al., 2014, 2017). HGFA treatment also appears to alert skin and hair follicle stem cell pools (Rodgers et al., 2017), arguing organism-wide injury-induced stem cell activation is an evolutionarily conserved strategy to enhance regeneration and repair.

In our model of systemic muscle repair, Hb-EGF may act over long distances to promote muscle regeneration. Hb-EGF expression was induced outside the myotome in response to systemic muscle injury, and the MuSC population most affected in *hbegfa* crispants were tissue-resident MuSCs interstitially positioned among the fast myofibers. In addition, Hb-EGF protein injected into circulation induced MuSC proliferation at a distance from the injection site. These data are consistent with a model in which long-range Hb-EGF signaling across tissues directs systemic muscle repair.

While the *in vivo* function of Hb-EGF during mammalian muscle regeneration has not been identified, Hb-EGF stimulated bovine MuSC proliferation in culture (Thornton et al., 2015). Outside the nervous system, Hb-EGF is produced by both macrophages and endothelial cells in the liver after ischemia injury, and is required for hepatocyte proliferation during liver regeneration (Wu et al., 2024; Yang et al., 2024). In zebrafish, ependymal cells express Hb-EGF in response to spinal cord injury, and fish treated with recombinant Hb-EGF showed improved functional regeneration (Cigliola et al., 2023). Hb-EGF expression is also upregulated in response to olfactory epithelium injury, where it promotes proliferation of sensory neuron precursors and accelerates recovery (Sireci et al., 2024). Hb-EGF is thus a pro-regenerative factor under multiple injury contexts and activates the proliferation of reparative stem cells and cell type-specific progenitors. Although the molecular mechanisms by which injury activates Hb-EGF expression has been investigated in some detail (Cigliola et al., 2023), the intracellular pathways and targets that direct stem cell activation or progenitor proliferation in response to Hb-EGF are not known. Further investigation into these downstream pathways will be essential for understanding how MuSCs are activated in response to tissue cross-talk and may reveal important insights into the mechanisms by which stem cells illicit specific responses to individual growth factors.

A scalable muscle injury model was previously developed in zebrafish, in which either a single myofiber was injured or all the myofibers in a single somite were injured (Knappe et al., 2015). In this model the single myofiber injury recruited Pax7a-expressing cells for repair while the larger injury did not, arguing there is a differential response among MuSC population. In contrast, the local muscle injury used in this study, which is analogous to the larger somite injury reported by Knappe et al. (2015), appeared to activate the same MuSC populations as systemic injury, at least by scSeq analysis (Fig. 3F,H). The most significant difference in our informatics analysis at the cell population level was that systemic injury activated more MuSCs than local injury (Fig. 3F). Molecularly, Hb-EGF was required for systemic but not local repair (Fig. S5B). Systemic repair appears to require more MuSC activation than local repair, and Hb-EGF may act upstream of the local growth factors that activate MuSCs to indirectly amplify the magnitude of the MuSC response. Our transcriptomic analysis argues Hb-EGF also signals directly to MuSCs, suggesting Hb-EGF acts at multiple levels to activate MuSCs. Identifying the cellular and molecular targets of Hb-EGF signaling during muscle regeneration will provide an entry point for defining the regenerative programs that direct systemic repair.

If local and systemic muscle injuries induce distinct regenerative programs, then there must be a mechanism that evaluates the scale of the injury and elicits the appropriate response. Local injuries activate gene expression changes in remote tissues, suggesting injury information is delivered over long distances to coordinate an organism-wide response. For example, cardiac damage in zebrafish induces transcriptional changes in brain ependymal cells and kidney tubular cells through hormone signaling (Sun et al., 2022). The roles of long-range tissue interactions and inter-organ communication pathways are thus emerging as essential components of the regenerative response. It remains unclear if local and systemic muscle injuries activate inter-organ communication pathways that deliver injury information to distant tissues that, in turn, decipher the amplitude of an injury to activate the appropriate regenerative program. Our transcriptomic and genetic analyses, however, have revealed new insights into the organism-wide response to muscle injury, and suggest that systemic repair requires multiple inputs outside of the MuSC niche.

One limitation of our study is that systemic injury damaged myofibers that were subsequently repaired while local injury caused myofiber apoptosis. It is possible that the differential transcriptional responses we observed between the injury models is due to the type of myofiber damage and not the scale of the injury. While the type of myofiber injury likely determines some aspect of the regenerative response, systemic injury induced more MuSC activation and the activated MuSCs themselves were transcriptionally similar to the activated MuSCs induced by local injury (Fig. 5E,F). These results argue that the MuSC response to each injury is similar, but the magnitude of the response is dictated by the scale of the injury. A NTR-MTZ platform with temporal and spatial control of injury would enable comparative studies of the regenerative responses to NTR-MTZ-induced myofiber damage versus needlestick-induced myofiber cell death in a localized region of the myotome. This model could provide new insights into the mechanisms by which muscle diseases associated with repeated myofiber damage and repair, e.g. muscular dystrophies, activate or disrupt the regenerative response.

While mammals can regenerate small-scale muscle injuries, large-scale muscle injuries, such as trauma-induced muscle, loss do not fully repair and can lead to debilitative disabilities (Sun et al., 2022). To improve large-scale muscle repair, bioengineering approaches are being developed for the localized delivery of bioactive molecules at the site of muscle loss (Haas et al., 2021). However, conflicting outcomes argue growth factor loaded scaffolds are not yet fully optimized to treat major muscle injuries (Gahlawat et al., 2024). Pro-regenerative molecules discovered in zebrafish can improve muscle outcomes following large-scale muscle injuries in mammals (Ratnayake et al., 2021). One exciting possibility is that the regenerative programs used for systemic muscle repair in teleosts are simply dormant in mammals. If this is the case, activators of systemic repair programs in zebrafish would be attractive candidates to induce analogous regenerative responses in mammals and treat large-scale muscle injuries.

## MATERIALS AND METHODS

### Zebrafish

Zebrafish (*Danio rerio*) of the Tubingen genetic background were maintained at the Washington University Zebrafish Core Facility. All experimental protocols were approved by the Washington University School of Medicine Institutional Animal Care and Use Committee. Male and female larvae of the indicated ages were used for regeneration experiments. For all experiments, multiple cohorts (clutches) of injured fish were used. The number of animals for each experiment is reported in the figure or figure legend. The following published strains were used: *Tg(-80.0myf5:GFP)* (Chen et al., 2007), *Tg(HuC:EGFP)* (Park et al., 2000), *Tg(fli1:EGFP)* (Lawson and Weinstein, 2002), *Tg(cmlc2:EGFP)* (Burns et al., 2005) and *Tg(en1b-hs:Gal4)* (Kimura and Higashijima, 2019).

### Muscle injury

To induce a systemic injury, 50 larvae per experimental group were treated for 12 h with 5 mM of MTZ. Uninjured siblings were used as controls. Localized muscle injury was carried out as described previously (Gurevich et al., 2016). Zebrafish were anesthetized on a petri dish using 4 mg/ml tricaine solution (Syndel, 200-226). Localized injury was performed using size 0 insect pins (Roboz Surgical Instruments, RS-6081-35) at a 90° angle in three dorsal somites positioned above the cloaca. Injured fish were then transferred to a fresh petri dish with embryo water to recover.

### Generation of transgenic lines

For all transgenic lines, two founders with the most consistent levels of expression and activity were maintained and outcrossed to wild-type (Tu). For lines that lack a fluorescent marker, the F_1_ generation was in crossed to make multicopy lines that were validated by outcrossing and assessing Mendelian segregation. Lines with fluorescent markers were maintained as single copy. Lines derived from independent founders behaved similarly with respect to expression and activity, arguing our selection strategy minimized position effects between insertions. For each transgene, a single founder line was used for downstream experiments. Double and triple transgenic lines were generated using standard genetic crosses. Details of primers used in the study are provided in Table S4.

#### Tg(*actc1b*:NTR-2A-mCherry)

actc1F and actc1R primers were used to amplify a 3.5 kb region proximal to the *actc1b* promoter. This enhancer has previously been characterized as skeletal muscle specific (Higashijima et al., 1997). The genomic fragment was cloned into pCR2.1, then subcloned into a BamHI-digested PCS2-mCherry-Nitroreductase (NTR1.0) plasmid (Zhou et al., 2023) to generate pCS2. *actc1b*:NTR-2A-mCherry. This plasmid was co-injected with I-SceI into zebrafish embryos at the one-cell stage. Founders were identified by mCherry expression and confirmed by genotyping using actc1genoF and NTRgenoR primers. Ten founder lines were tested for muscle injury.

#### Tg(*actc1b*:NTR-2A)

The pCS2. *actc1b*:NTR-2A-mCherry plasmid was digested with XhoI to remove the mCherry-coding sequence and ligated. The plasmid was injected as described above and ten founder lines were tested for muscle injury.

#### Tg(*actc1b*:CreER^T2^)

Two EcoR1 sites were added to the 3.5 kb *actc1b* enhancer using standard PCR, cloned into pCR2.1 and then subcloned into EcoR1-digested pCS2.Flag.CreER^T2^ (Zhou et al., 2023) to generate pCS2.*actc1b*:CreER^T2^. The plasmid was co-injected with I-SceI into zebrafish embryos at the one-cell stage. Founders were genotyped with CreF and CreR primers. Twenty founder lines were crossed with the Tg(*ubb:LOXP-H2B-mCherry-STOP-LOXP-SFGFP*) recombination reporter line (a gift from Jimann Shin, Washington University in St. Louis, MO, USA) and offspring were treated with 5 μM tamoxifen (Sigma T5648) for 16 h to identify founders with robust recombination activity. Neither line activated GFP in the absence of tamoxifen.

### CRISPR/Cas9 mutagenesis

CRISPR/Cas9 design and mutagenesis was followed according to previous methods (Hoshijima et al., 2019; Klatt Shaw et al., 2021). crRNAs were designed using CHOPCHOP (Labun et al., 2019) against the GRCz11 zebrafish genome. crRNA sequences with no off-target sites, minimum mismatches and highest efficiency were chosen. Alt-R modified crRNAs ordered from IDT (2 nmol scale) and tracrRNAs (IDT, 1073190) were resuspended according to manufacturer's specifications. tracrRNA and crRNA were mixed and diluted to a final concentration of 50 μM, annealed by heating to 95°C and gradual cooling at 2°C/s to 25°C and stored at −20°C. For injections, duplexed dgRNAs were diluted 1:1 in duplex buffer (IDT, 1072570) to a working concentration of 25 μm. Equal volumes of dgRNA and Cas9 (IDT, 1081058) were incubated with 37.5 mM KCl and injection dye for 37°C for 5 min. For co-injections with two guides, Cas9 was added in equal molar concentration as the total gRNA concentration. Embryos at the one-cell stage were injected with ∼1 nl of CRISPR/Cas9 solution. Details of the CRISPR (crRNA) guides used in this study are provided in Table S5.

#### pax7a^GFP-2A-NTR2.0^ knock-in

A pCS2.pax7a.GFP-2A-NTR2.0 construct was generated by HiFi Assembly (New England Biolabs, E5520S) comprising two 1 kb genomic homology arms that flanked the *pax7a* stop codon and the GFP-2A-NTR2.0 cassette (Sharrock et al., 2022). To generate the GFP-2A-NTR2.0 knock-in, the crRNA *pax7a*.KI was duplexed and mixed with Cas9, KCl and injection dye, as described previously (Klatt Shaw and Mokalled, 2021). The template plasmid pCR2.pax7a.GFP-2A-NTR2.0 (25 ng/μl final concentration) and non-homologous end-joining inhibitor NU7441 (Selleckchem, 50 mM final concentration) were then added to the injection mixture. Embryos at the one-cell stage were injected with ∼1 nl of CRISPR/Cas9 solution. Founders were identified by PCR screening of F_0_ larvae for GFP (using GFPF and GFPR primers) and then confirmed with junctional primers that spanned the *pax7a* locus and the GFP-2A-NTR2.0 cassette (using Pax7AF and Pax7AR primers).

#### smyhc1^sfgfp^ knock-in

A pMK-RQ.smyhc1.GFP construct (generated by Invitrogen) comprising two 1 kb genomic homology arms that flanked the *smyhc1* stop codon and a 714 bp superfolder GFP-coding sequence was used as a template for homology-dependent repair. To generate the GFP knock-in, the crRNA *smyhc1*.GFP.KI was injected with the template plasmid pMK-RQ.smyhc1.GFP, and the injection cocktail described above.

#### hbegfa deletion

hbegfa.1 and hbegfa.2 crRNAs targeting the first and last coding exons of *hbegfa* were duplexed and injected as previously described. hbegfadelF, hbegfadelR and hbegfahetR primers were used to genotype the F_1_ and identify founders. Two founder lines were maintained.

### Recombinant protein injection

4 dpf larvae were anesthetized with tricaine and positioned laterally in custom molds. Injection needles were filled with PBS or Hb-EGF protein (Sigma E4643) and 1 µl Dextran-Alexa 594 (Invitrogen D22913), and larvae were injected in the duct of Cuvier as described previously (Benard et al., 2012). Larvae were live imaged 24 h after injection.

### Live imaging

Larvae were immobilized in imaging chambers with 1% low melt agarose. Fish water supplemented with tricaine was added to the chamber after the agarose had set. Live images were taken using a Zeiss LSM 800 confocal microscope or a Nikon W1 CSU SoRa spinning disk confocal.

### Histology

Larvae were either dissected to remove heads for genotyping or fixed whole with 4% paraformaldehyde in PBS for 1 h. Samples were then rinsed four times for 10 min each in PBT, transferred to 30% sucrose in PBS and incubated overnight at 4°C. Samples were embedded in tissue-freezing medium (General Data Healthcare, TFM-C), frozen on dry ice and stored at −80°C. 16 µm cross cryosections were generated for downstream application. Tissue sections were imaged using a Zeiss LSM 800 confocal microscope for immunofluorescence and HCR.

For hybridization chain reaction fluorescent *in-situ* hybridization (HCR-FISH), probes were designed against target mRNAs using an automated program (Kuehn et al., 2022) and ordered from IDT as pooled oligonucleotides (10 pmol scale). The HCR protocol was adapted from Choi et al. (2018) and Klatt Shaw et al. (2024 preprint). Slides were dried on a warmer for 1 h before dehydrating for 3 min each in 50% ethanol in PBT, 95% ethanol in PBT and 100% ethanol. They were hydrated in 50% PBT in ethanol, 95% PBT in ethanol and 100% PBT. Slides were treated with 0.2% TritonX-100 in PBS, placed in PBT five times for 5 min each. After drying the slides, samples were enclosed within a hydrophobic barrier. For blocking, slides were incubated for 1 h at 37°C in 200 μl of pre-warmed hybridization buffer (Molecular Instruments) per slide. Probes were prepared by adding 1 μl of each desired probe with 200 μl of pre-warmed hybridization buffer. The pre-hybridization buffer was replaced with the probe in hybridization buffer and sections were incubated at 37°C in the dark for 48 h. Slides were washed in 100 μl of wash buffer (Molecular Instruments) at room temperature four times for 5 min each followed by 5×SSCT [3 M NaCl, 0.3 M sodium citrate and 0.1% Tween-20 (pH 7.0)] twice for 5 min each. The sections were incubated in 200 μl of amplification buffer (Molecular Instruments) per slide for 1 h at room temperature. Prior to amplification, hairpin RNAs were preheated for 90 s at 95°C and cooled for 30 min. For amplification, each hairpin RNA was diluted 1:50 in amplification buffer and incubated at room temperature in the dark for ∼24 h. Samples were washed twice for 5 min each in 5×SSCT at room temperature. Sections were mounted in 60 μl Fluoromount-G, incubated at 4°C overnight and sealed with nail polish.

Immunohistochemistry (IHC) was carried out as described in The Zebrafish Book (Westerfield, 2000). Briefly, tissue samples were restricted within a hydrophobic boundary with an ImmEdge barrier pen (Vector Laboratories, H-4000) and rehydrated in PBT (0.1% Tween-20 in PBS). After two 5 min washes, samples were treated with a mix of 0.1% Triton, 1% BSA and 1% DMSO in PBS, and rinsed in PBT four times for 5 min. Tissue sections were blocked in 2% goat serum in a mix of 1% BSA, 1% DMSO in PBS for 1 h at room temperature. Samples were incubated overnight with primary antibody diluted in blocking agent at 4°C in the dark followed by four 5 min washes with 1% BSA and 1% DMSO in PBS. Samples were then incubated in secondary antibody in the wash solution for 2 h at room temperature in the dark followed by four 5 min washes with 1% BSA, 1% DMSO in PBS. Samples were incubated with 1 μg/ml of Hoechst for 5 min and rinsed twice with PBT for 5 min.

Primary antibodies used in the study were: mouse anti-4D9 (DSHB, 1:10), mouse anti-PAX7 (DSHB, 1:10), mouse anti-MF 20 (DSHB, 1:400), rabbit anti-DsRed (Living Colors, 632496, 1:400), chicken anti-GFP (Aves Labs, GFP-1020, 1:1500), rabbit anti-PCNA (Genetex, GTX124496, 1:250) and rabbit anti-D175 (Cell Signaling Technology, 966, 1:500). Secondary antibodies used were Alexa Fluor 488 goat anti-mouse (Invitrogen, A-21121, 1:250), Alexa Fluor 594 goat anti-rabbit (Invitrogen, A-11072, 1:250), Alexa Fluor 488 goat anti-chicken (Invitrogen, A-11039; RRID: AB_142924, 1:250).

### Quantification

Immunohistochemistry and HCR-FISH was performed on 3-5 serial transverse sections per fish. Quantification was performed on single-plane projected confocal images. The cell counter tool in ImageJ was used to quantify the number of cells that expressed specific proteins. To quantify HCR fluorescence, user-defined color thresholds were set manually in ImageJ. Integrated density was quantified for fluorescence signals inside a region of interest using the ‘Analyze Particles’ command.

### Statistical analyses

Statistical analyses for histology and functional assays were performed with GraphPad Prism software package and included unpaired Student's *t*-test (with Welch's correction in cases of non-Gaussian distribution) and one-way ANOVA. Appropriate corrections for multiple pairwise comparisons among groups were applied.

### Isolation of single cells from larval zebrafish

15 larvae per cohort were dissected to remove the head, caudal fin and internal organs, and then processed as described (Farnsworth, DB 459 2020). For tissue lysis, 500 μl of dissociation buffer (2 μg/μl collagenase P, 2 mM CaCl_2_, 0.2 μl DNaseI and 0.25% Trypsin in 1×PBS) was added to the samples and incubated at 28°C until samples were visually dissociated (∼15 min). Dissociation was stopped with equal volume of stop buffer (10% FBS and 0.001 mM EDTA in 1×PBS). Samples were centrifuged at 750 ***g*** for 7 min at 4°C, the supernatant was discarded and the pellet was resuspended in 100 μl of resuspension buffer (1% FBS, 2 mM CaCl_2_ and 1×penicillin/streptomycin in DMEM). The suspension was strained through 20 μm cell strainer (Pluriselect USA, 43-10020-40), centrifuged once (500 ***g*** for 1 min at 4°C) to pass cells through the strainer, and centrifuged a second time (750 ***g*** for 7 min at 4°C) to pellet the cells. The cells were resuspended in 100 μl of resuspension buffer, centrifuged (750 ***g*** for 7 min at 4°C) and the pellet was resuspended in 50 μl 0.04% BSA in PBS. 5 μl of cells were then combined with 5 μl of Hoechst (Invitrogen, H3570), incubated for 10 min and transferred to a hemocytometer (Bulldogbio, NC1731934) to determine cell concentration. An additional 5 μl of cells were incubated with Trypan blue (Sigma, T8154) for 5 min, transferred to a hemocytometer and counted to assess cell viability. Samples with >70% viability were then submitted for single-cell sequencing.

### scSeq

For scSeq, 30 µl of resuspension solution containing isolated cells at a concentration of ∼1000 cells/µl were submitted to the Genome Technology Access Center in the McDonnell Genome Institute at Washington University. One biological replicate was used for each time point. cDNA was prepared after GEM generation and barcoding, followed by the GEM-RT reaction and bead cleanup steps. cDNA was amplified for 11-13 cycles then purified using SPRIselect beads. Purified cDNA samples were then run on a Bioanalyzer to determine cDNA concentration. GEX libraries were prepared as recommended by the 10x Genomics Chromium Single Cell 3′ Reagent Kits User Guide (v3.1 Chemistry Dual Index) with appropriate modifications to the PCR cycles based on the calculated cDNA concentration. For sample preparation on the 10x Genomics platform, the Chromium Next GEM Single Cell 3′ Kit v3.1, 16 rxns (PN-1000268), Chromium Next GEM Chip G Single Cell Kit, 48 rxns (PN-1000120) and Dual Index Kit TT Set A, 96 rxns (PN-1000215) were used. The concentration of each library was accurately determined through qPCR utilizing the KAPA library Quantification Kit according to the manufacturer's protocol (KAPA Biosystems/Roche) to produce cluster counts appropriate for the Illumina NovaSeq6000 instrument. Normalized libraries were sequenced on a NovaSeq6000 S4 Flow Cell using the XP workflow and a 50×10×16×150 sequencing recipe according to manufacturer protocol. A median sequencing depth of 50,000 reads/cell was targeted for each Gene Expression Library.

### Aligning scSeq reads

After sequencing, the Illumina output was processed using the CellRanger (v8.0.1) pipeline to generate gene-barcode count matrices. A custom reference genome was made with the ‘cellranger mkref’ command, using the fasta file of zebrafish reference genome GRCz11 constructed from the Ensemble genome build (https://useast.ensembl.org/Danio_rerio/Info/Index) and the sorted Gene Transfer Format file (v4.3.2) from the improved zebrafish transcriptome annotation (Lawson et al., 2020). Base call files for each sample from Illumina were demultiplexed into FASTQ reads. Then, the ‘cellranger count’ pipeline was used to align sequencing reads in FASTQ files to the custom reference genome. Both exon and intron sequences were included during the alignment. The filtered gene-barcode count matrices generated by ‘cellranger count’ was used for downstream analysis.

### Quality control

DecontX package (v1.2.0) was used to remove droplets containing aberrant counts of ambient mRNA (Yang et al., 2020). The function ‘decontx’ was used on the raw ‘RNA’ counts to obtain ‘decontX’ count data, following the default pipeline. DecontX counts were used as default for pre-processing and normalization. After ambient RNA removal, DoubletFinder (v2.0.4) was used to identify doublets formed from transcriptionally distinct cells (McGinnis et al., 2019). Optimal pK values were calculated from the outputs of function ‘paramsweep’. The ‘doubletFinder’ function was then used to predict doublet cells, where 50 principal components and pN value of 0.25 were given as input along with the previously calculated pK value of 30. All the cells predicted as doublets were removed.

### Integrated analysis of scSeq dataset

Datasets were integrated and analyzed using Seurat (v4.4.0) package with R (v4.4.2) (Stuart et al., 2019; R Core Team, 2024). Each sample count matrix was filtered for genes that were expressed in at least three cells and cells expressing at least 200 genes, followed by cell quality assessment using commonly used QC matrixes (Ilicic et al., 2016). Cells with a unique number of genes between 200 and 6500, a mitochondrial gene percentage <5 and total number of reads (UMIs) between 300 and 35,000 were used for downstream processing. Each dataset was independently normalized and scaled using the ‘SCTransform’ function, which is an improved method for normalization, that performs a variance-stabilizing transformation using negative binomial regression (Hafemeister and Satija, 2019). Standard integration workflow of Seurat was used to identify shared sources of variation across experiments as well as mutual nearest neighbors (Butler et al., 2018; Haghverdi et al., 2018). Integration features were selected based on the top 6000 highly variable features using ‘SelectIntegrationFeatures’ function (nfeatures=6000), which was used as input for the ‘anchor.features’ argument of the ‘FindIntegrationAnchors’ function. PCA analysis was performed on the 6000 variable features and the top 50 principal components were selected based on the elbow plot heuristic, which measures the contribution of variation in each component. These 50 principal components were used in ‘FindNeighbors’ and ‘FindClusters’ functions to perform graph-based clustering on a shared nearest-neighbor graph (Levine et al., 2015; Xu and Su, 2015). The Louvain algorithm was used for modularity optimization in cell clustering using ‘FindClusters’ function. The resolution parameter (res=0.3) that determines the granularity of clustering was selected by visually inspecting clusters with resolutions ranging between 0.1 and 2.0, as well as clustree graph (Zappia and Oshlack, 2018). Uniform Manifold Approximation and Reduction (UMAP) was used for non-linear dimensional reduction of the first 50 principal components and the data visualized using ‘RunUMAP’ function (Becht et al., 2018). Data were graphed using different plot functions, such as ‘DimPlot’, ‘VlnPlot’, ‘FeaturePlot’, ‘Dotplot’ and ‘DoHeatmap’, to view the cell cluster identity and marker gene expression. Cell proportions were extracted using the ‘table’ and ‘prop.table’ functions. Differential gene expression for individual clusters was identified using Wilcoxon rank sum test in the ‘FindAllMarkers’ function. Marker genes detected in at least 25% of the clustered cells with a positive average log_2_(FC) were reported.

### Cluster identification using differentially expressed markers

We used a zebrafish single atlas (Sur et al., 2023) to generate a database of markers for each tissue and cell type (Table S1). The differentially expressed markers of each cluster were cross-referenced with our compiled database using our ‘DE-Marker-Scoring’ algorithm (Saraswathy et al., 2024). For every matching marker gene, one point was given to the respective cluster under the column name with matching cell identity. Iteration over every marker gene was performed to generate a scoring matrix with varying points for each cluster against the different cell identities compiled in the database (Table S2, sheet: scoring). The ‘phyper’ function in R was then used to calculate one-tailed binomial probabilities using hypergeometric distribution for the total score obtained from each cluster against each cell identity in the database (Table S2, see ‘Binomial probability’ sheet). −log10 of probability values were obtained for plotting the heatmap (Table S2, see ‘−log10P’ sheet). The resulting values were scaled from 0 to 100 and plotted as a heatmap using GraphPad prism. Each cluster was given an identity based on the maximum −log10 p-score obtained in the heatmap. The top DE markers of clusters with ambiguous scores were manually annotated using the ‘ident genes’ from the zebrafish single atlas (Sur et al., 2023) and literature search (McKellar et al., 2021). Top DE markers generated for each cluster are provided in Table S3 (see ‘topDEmarkers.all.clusters’ sheet). The ‘RenameIdents’ function was used to assign identity to each cluster. To confirm the assigned cluster identities, enrichment of classical markers of respective cell types were tested using Dot plot.

### Subset analysis of muscle clusters

Muscle cells identified from the complete dataset were subclustered using the ‘subset’ function for subcluster analysis. The muscle subset was again normalized and scaled using the ‘SCTransform’ function with the glmGamPoi method (Ahlmann-Eltze and Huber, 2021). Fifty principal components were used and the resolution parameter was set to 0.4. Downstream analysis was carried out as described above for the integrated analysis. The top DE markers generated for each muscle subcluster using ‘FindAllMarkers’ function are provided in Table S3 (see ‘topDEmarkers.muscle.clusters’ sheet).

### Cell-cell interaction assay

The R package CellChat (v2.1.2) was used to evaluate regenerative cell-cell interactions after local and systemic muscle injury (Jin et al., 2021). CellChat models the probability of cell-cell communication by integrating our gene expression data with a database of known interaction between signaling ligands, receptors and their co-factors (CellChatDB). The RNA data were used to create CellChat object using ‘createCellChat’ function, followed by the recommended preprocessing functions with default parameters for the analysis of individual datasets. Truncated mean method with 10% trimmed observation was used to compute average gene expression per cell group for the complete dataset. The default trimean method was used to compute average gene expression for the CellChat analysis. CellChatDB.zebrafish was used to infer cell-cell communication. All categories of ligand-receptor interactions in the database were used in the analysis. Communications involving fewer than 10 cells were excluded. The ‘netAnalysis_computeCentrality’ function was used to calculate network centrality scores at each time point. Functions such as ‘netVisual_circle’, ‘netAnalysis_contribution’, ‘netVisual_aggregate’, ‘netVisual_bubble’, ‘netAnalysis_signalingRole_heatmap’ and ‘netAnalysis_signalingRole_scatter’ were used to generate different plots used in this article.

## Supplementary Material



10.1242/develop.204993_sup1 Supplementary information

Table S1. The *Danio rerio* Muscle Marker (DMM) database created using Daniocell atlas by (Sur et al., 2023).

Table S2. A. Full dataset scoring output from the DE-Marker-Scoring algorithm. B. Full dataset normalized scoring output from the DE-Marker-Scoring algorithm. C. Full dataset hypegeometric probabilty output from the DE-Marker-Scoring algorithm. D. -log10 of hypergeometric probability of full dataset clusters. E. Subset of the DMM database entries found in the differentially expressed marker list that were used for cell type identification.

Table S3. **A.** The top differentially expressed markers of clusters in the full dataset. **B.** The top differentially expressed markers of clusters in the muscle subcluster dataset.
