# Supplementary material for: Hb-EGF directs systemic muscle repair
Source: Development. 2026 Mar 23;153(6):dev204993. doi: 10.1242/dev.204993 (PMC13495789; doi:10.1242/dev.204993)
Supplement: Supplementary file 1 [file develop-153-204993-s1.pdf]

Figure S1

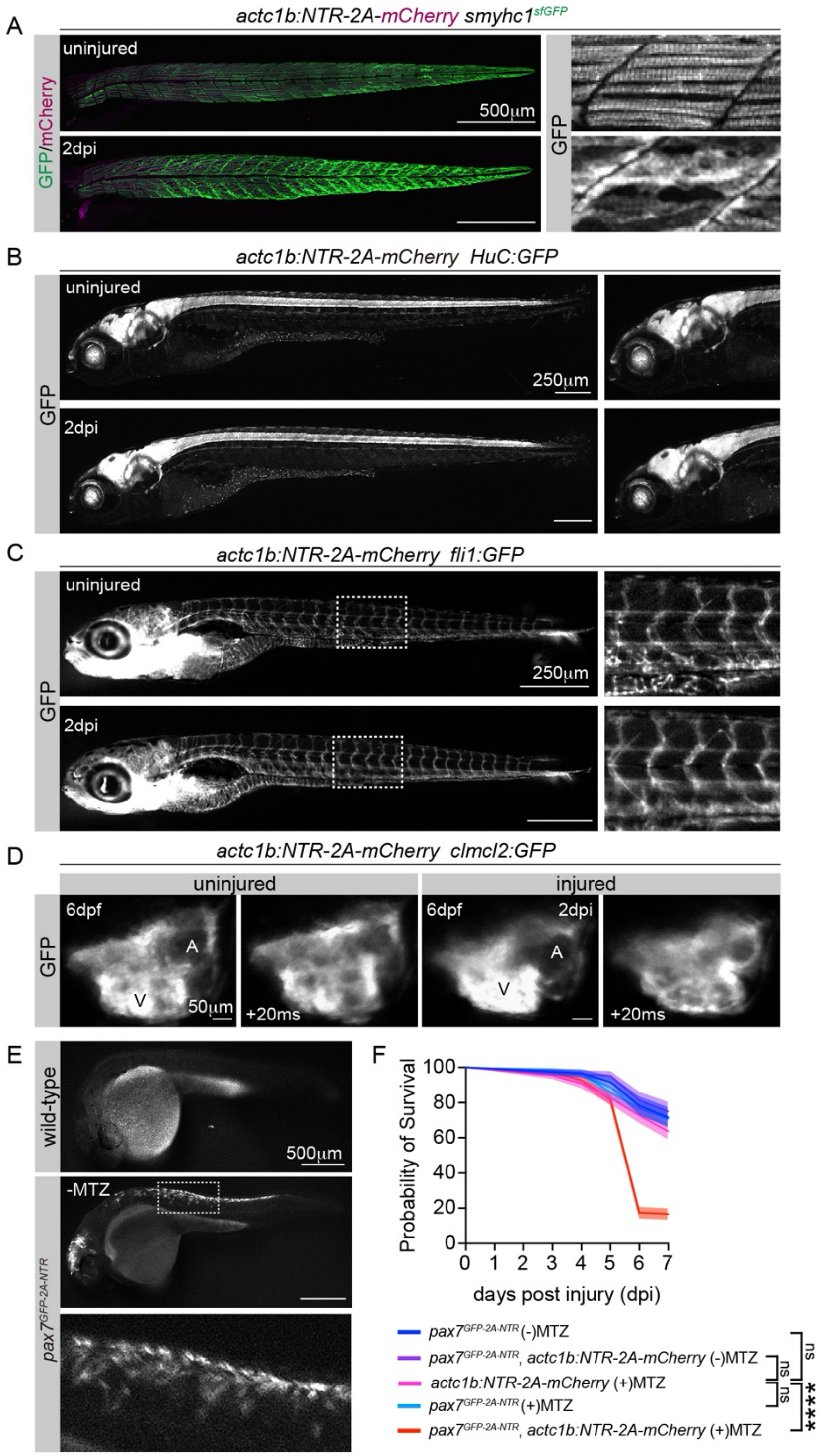

Development • Supplementary information

Fig. S1. *smyhc1* is expressed in slow but not fast myofibers during systemic repair. A. Live imaging of Tg(*actc1b*:NTR-2A-mCherry) *smyhc1*^{sfgfp} larvae, showing Smyhc1:GFP (green) and mCherry (violet). Smyhc1:GFP aggregated to somite boundaries in injured larvae at 2dpi. High magnification views show injured myofibers are disorganized and unstriated. Representative images from n≥20 larvae per treatment group. **B-D.** Tg(*actc1b*:NTR-2A-mCherry) larvae double transgenic for *Tg(HuC:EGFP)* (nervous system; **B**), *Tg(fli1:EGFP)* (vasculature; **C**), or *Tg(cmlc2:EGFP)* (heart; **D**). Non-muscle tissues were morphologically normal after muscle injury. Lateral images of the heart were taken 20ms apart to show heart contractions in uninjured and injured fish. n=5 larvae per treatment group per transgene. **E.** Live imaging of uninjured *pax7a*$^{GFP\text{-}2A\text{-}NTR}$ embryos and non-transgenic sibling controls. GFP(+) cells were visible in the head and trunk of *pax7a*$^{GFP\text{-}2A\text{-}NTR}$ knock-in fish at 26 hours post fertilization. **F.** Survival curve. *pax7a*$^{GFP\text{-}2A\text{-}NTR}$ Tg(*actc1b*:NTR-2A-mCherry) larvae treated with MTZ showed significantly reduced survival compared to Tg(*actc1b*:NTR-2A-mCherry) MTZ treated controls. n≥40 larvae per treatment group. (ns) not significant, ****(p<0.0001). Significance was determined by log rank test. Dashed rectangles in (**C,E**) denote magnified regions in subpanels.

Development • Supplementary information

Figure S2

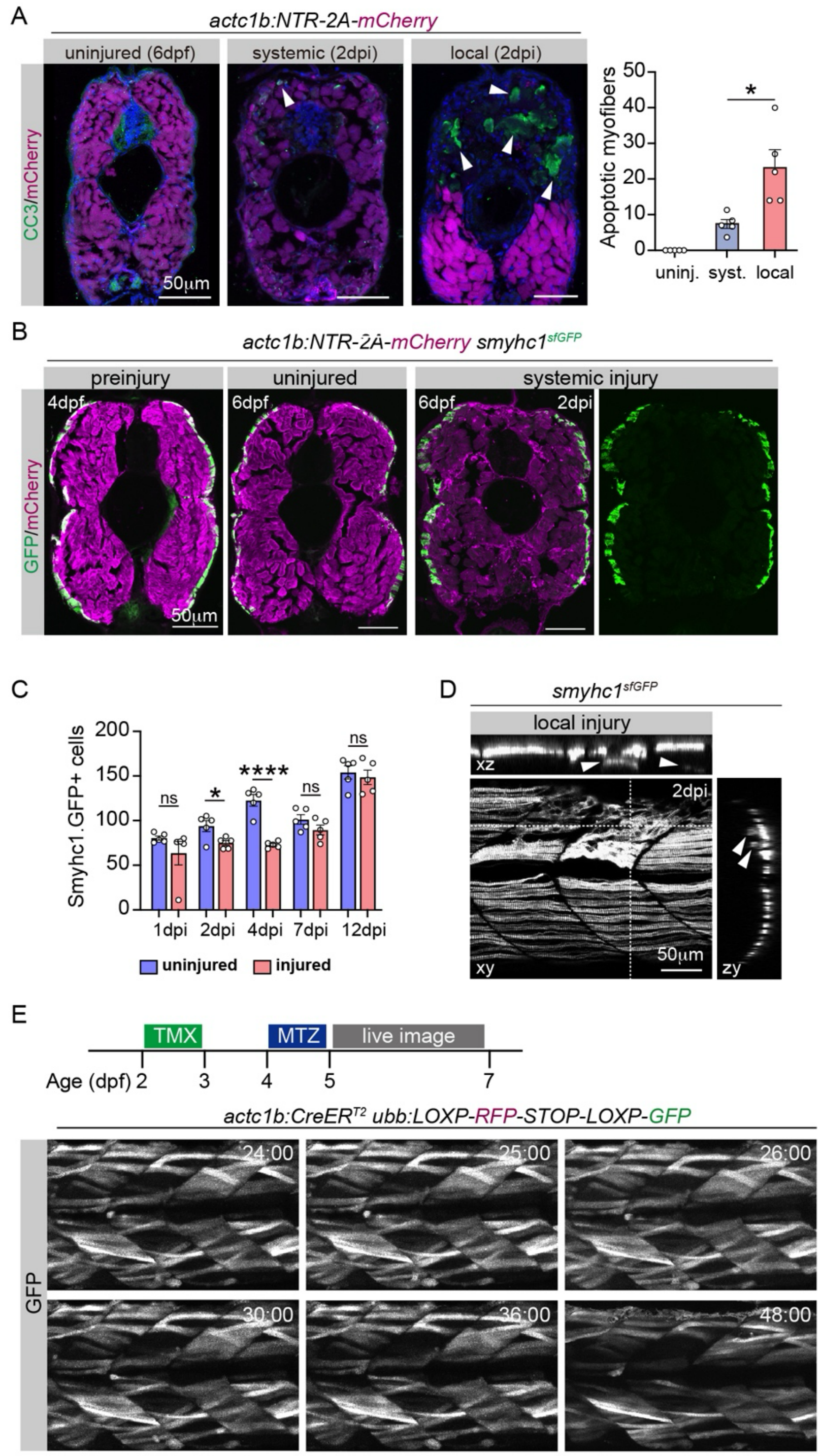

Development • Supplementary information

Fig. S2. Injured myofibers survive systemic injury. A. Transverse sections of Tg(*actc1b*:NTR-2A-mCherry) larvae labelled for mCherry (magenta) and cleaved Caspase 3 (green). Myofibers from uninjured larvae lacked cleaved Caspase 3. MTZ in-jured (systemic) larvae had significantly fewer cleaved Caspase 3 positive myofibers than needlestick injured (local) larvae (arrowheads). Quantification of cleaved Caspase 3 ex-pression. Data points represent average number of cleaved Caspase 3 positive myofibers in three sections from one larva. **B.** Transverse sections of Tg(*actc1b*:NTR-2A-mCherry), *smyhc1*^{sfgfp} larvae from pre- and post-injury timepoints labelled for mCherry (magenta) and Smyhc1.GFP (green). Smyhc1.GFP was largely maintained in slow myofibers during systemic repair but was not activated in fast myofibers. **C.** Quantification of GFP+ myofi-bers shown in (**B**). GFP expression was reduced in slow myofibers at 2- and 4dpi. Data points represent average number of GFP positive myofibers in three sections from one larva. **D.** Live images of *smyhc1*^{sfgfp} larvae two days after needlestick injury. GFP positive myofibers were visible internal to slow muscles (arrowheads). **E.** Larvae triple transgenic for Tg (*actc1b*:NTR), (*actc1b*:CreER^{T2}), (*ubb:LOXP-H2B-mCherry-STOP-LOXP-SFGFP*) were treated with TMX for 24hr at 2dpf, and then MTZ-injured for 24hr at 4dpf. GFP+ larvae were live imaged for 24hr. Labelled myofibers survived systemic injury. Time series shown is representative of three imaged larvae.

Development • Supplementary information

Figure S3

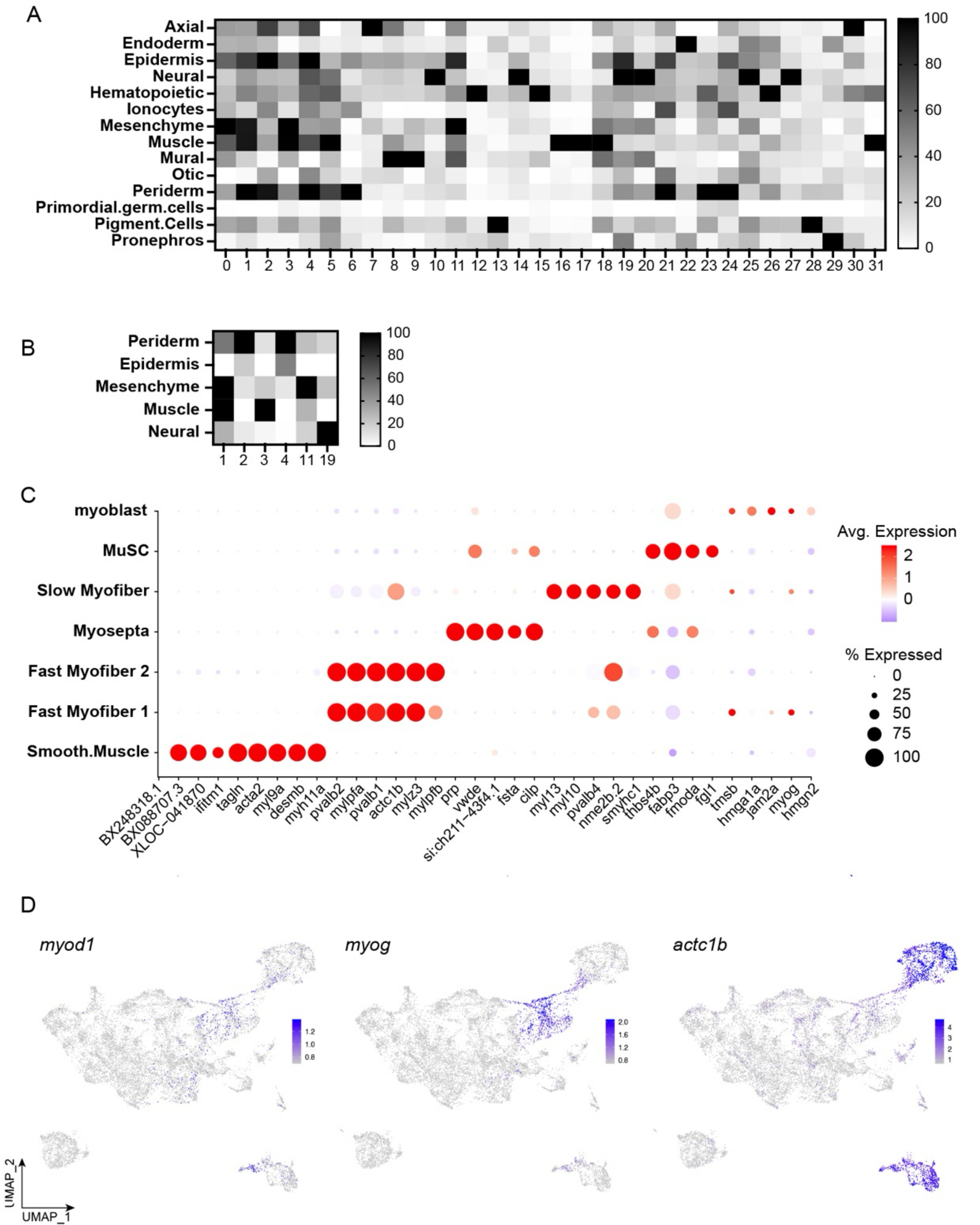

Development • Supplementary information

Fig. S3. Cluster identification and validation. A. Heatmap to define each cluster by tissue type using the DE-Marker-Scoring” algorithm as described (Saraswathy et al., 2024). Cluster numbers are on the horizontal axis; the cell types in our matrix are on the vertical axis. Our algorithm assigns a scaled probability based on the number of common genes between a known cell type and the marker genes in the cluster to produce the heat map. **B.** Six clusters from (**A**) associated with multiple cell types (1, 2, 3, 4, 11, and 19). We resolved these cluster identities by repeating the algorithm using only the “identity genes” from the zebrafish single cell atlas (Sur et al., 2023) to define the known cell types. **C.** Marker gene expression of muscle cell types in the complete data set. Dot plot shows top markers enriched in each cell type. **D.** Feature plots showing the distributions of dif-ferentiating myoblast markers (*myod*, *myog*) and the terminal differentiation marker *actc1b*.

Figure S4

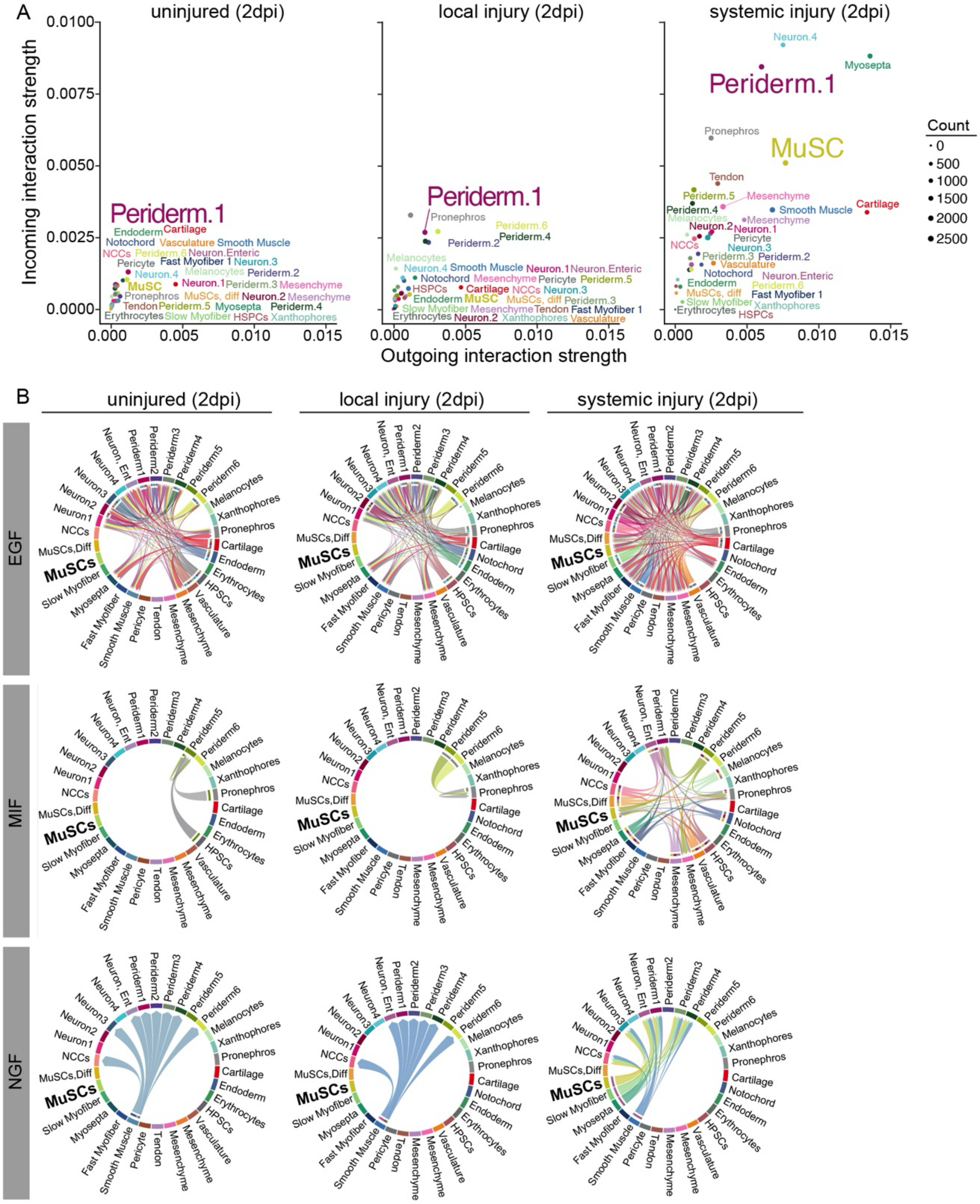

Development • Supplementary information

Fig. S4. Systemic injury activates unique cell-cell communication networks. A. Relative incoming and outgoing interaction strengths by cluster in uninjured, locally in-jured, and systemically injured samples at 2dpi. More clusters showed strong interactions in response to systemic injury than local injury or no injury. **B.** Chord diagrams showing Epidermal growth factor (EGF), Macrophage migration inhibitory factor (MIF), and Nerve growth Factor (NGF) intercellular communication at 2dpi. Notice only systemic injury in-duced EGF and MIF signaling to MuSCs or NGF signaling out of MuSCs.

Development • Supplementary information

Figure S5

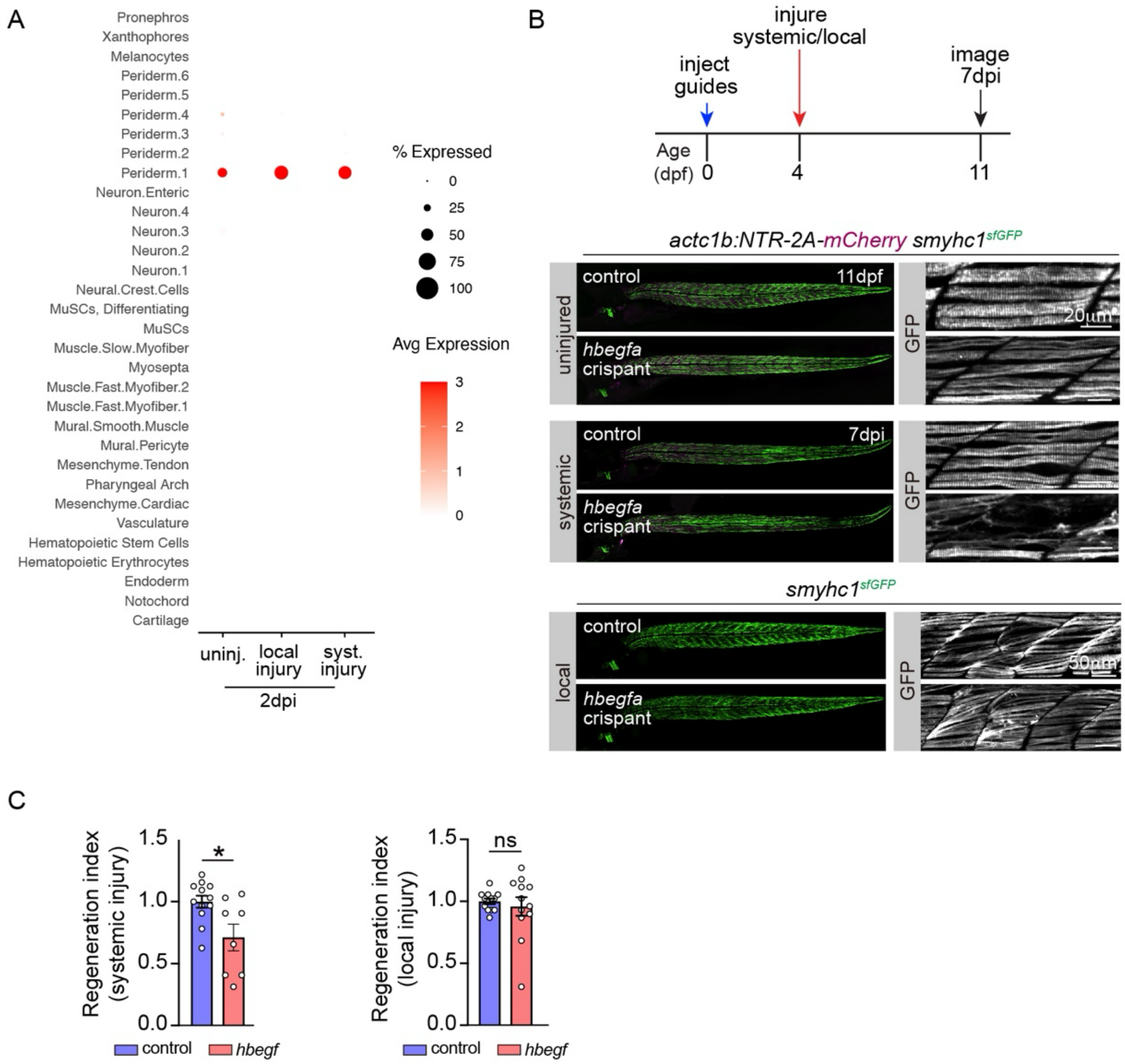

Fig. S5. ***hbega* is dispensable for local repair. A.** Dot plot of *frem2a* expression in the complete dataset. *frem2a* expression was restricted to the periderm. **B.** Experimental design for CRISPR-mediated deletion of *hbegfa*. Injected embryos were injured at 4dpf and collected at 7dpi for live imaging of Smyhc1:GFP (green). *hbegfa* crispants were defective in slow myofiber repair after systemic but not local injury. Uninjected siblings were used as controls. Uninjured crispants and control larvae showed similar muscle morphology (top panel). **C.** Quantification of regeneration. The number of Smyhc1:GFP positive myofibers were counted in three somites per larvae at 7dpi to calculate a regeneration index. *hbegfa* crispants showed significantly less slow myofiber repair after systemic injury compared to controls. Slow myofiber repair in response to local injury was comparable between crispants and controls. Data points represent individual larva. Error bars represent SEM. Unpaired students t-test. (ns) not significant (*) $p<0.05$.

Development • Supplementary information

Figure S6

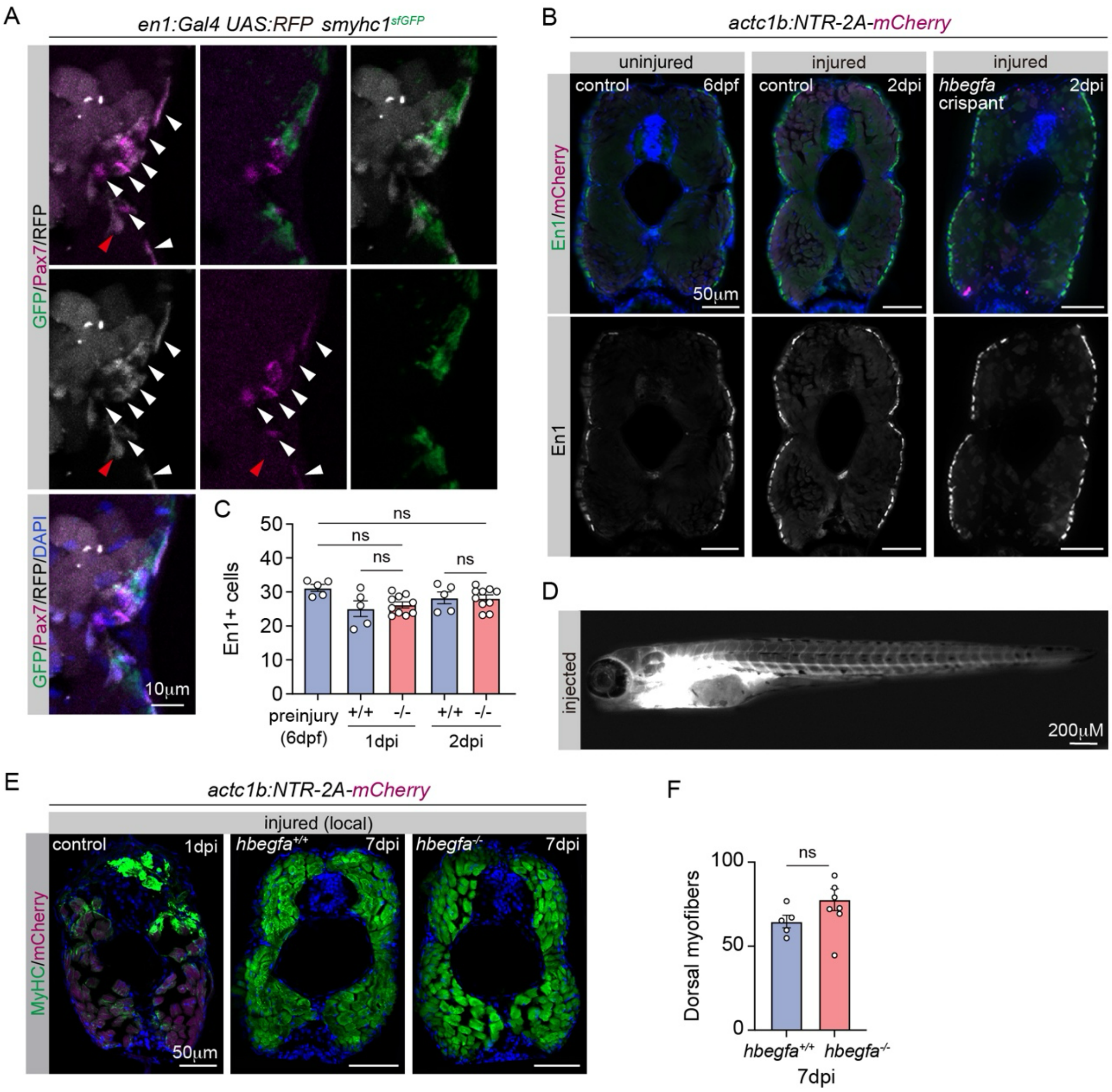

Development • Supplementary information

Fig. S6. Hb-EGF does not regulate the number of En1+ MuSCs and is dispensible for local muscle repair. A. Confocal micrograph of the trunk musculature of *smyhc1^{sfgfp}* Tg(*en1*:Gal4), (*UAS*:RFP) larvae labelled for DAPI (blue), GFP (green), Pax7(violet), and RFP (white) at 4dpf. Most En1+ cells were Pax7+ (white arrowheads), although some En1+ cells did not co-express Pax7 (red arrowhead). **B.** Transverse sections of the trunk musculature of Tg(*actc1b*:NTR-2A-mCherry) larvae labelled for En1 (green) at 2dpi. **C.** Quantification of En1+ cells from sections shown in (**B**). The number of En1 expressing cells in *hbegfa* crispants was comparable to uninjected sibling controls at 1- and 2-dpi. **D.** Live images of *pax7a$^{GFP\text{-}2A\text{-}NTR}$* injected with fluorescent Dextran-594 through the duct of Cuvier at 4dpf and live imaged 20 minutes later. Injected larvae showed 594-fluorescence throughout the vasculature. **E.** Transverse sections of Tg(*actc1b*:NTR-2A-mCherry) larvae at the site of local (needlestick) injury at 1- and 7-dpi, labelled for MyHC (green) and mCherry (red). Local injury caused MyHC aggregation at the injury site one day after injury. **F.** Quantification of myofiber number in the dorsal myotome from sections shown in (**E**). *hbegf$^{+/+}$* and *hbegfa$^{-/-}$* larvae showed comparable myofiber number at the site of injury (dorsal myotome) at 7dpi. Data points represent average myofiber number per section from a single larva. Error bars represent SEM. Significance was determined by unpaired students t-test. (ns) not significant.

Table S1. The *Danio rerio* Muscle Marker (DMM) database created using Daniocell atlas by (Sur et al., 2023).

Available for download at
https://journals.biologists.com/dev/article-lookup/doi/10.1242/dev.204993#supplementary-data

Table S2. A. Full dataset scoring output from the DE-Marker-Scoring algorithm. **B.** Full dataset normalized scoring output from the DE-Marker-Scoring algorithm. **C.** Full dataset hypegeometric probabilty output from the DE-Marker-Scoring algorithm. **D.** -log10 of hypergeometric probability of full dataset clusters. **E.** Subset of the DMM database entries found in the differentially expressed marker list that were used for cell type identification.

Available for download at
https://journals.biologists.com/dev/article-lookup/doi/10.1242/dev.204993#supplementary-data

Development • Supplementary information

Table S3. A. The top differentially expressed markers of clusters in the full dataset. **B.** The top differentially expressed markers of clusters in the muscle subcluster dataset.

Available for download at
https://journals.biologists.com/dev/article-lookup/doi/10.1242/dev.204993#supplementary-data

Table S4. Primers used in this study

actc1F	GATATCACTTTTACAATCTTACGCCCAAATTAG
actc1R	GATATCCTGGTTGGTCTGTGCAGG
actc1genoF	GTAGCGTGTGCATTTCTGTGT
NTRgenoR	CACCACATGGCTAGCATCCA
CreF	CGT ACT GAC GGT GGG AGA AT
CreR	GTGGCAGATGGCGCGGCAACA
GFPF	ATCATGGCCGACAAGCAGAA
GFPR	AACTCCAGCAGGACCATGTG
hbegfadelF	ACAGTCTTGCGTCTTTTCGTC
hbegfadelR	AGCGGCTCCCAGTTTAATCTT
hbegfahetR	ATCGGCAACCCATAATCCCC
PAX7AF	TCTCCAGCTGCTGTGGACTA
PAX7AR	TGGAAATGGCTCATGTGTGT

Table S5. CRISPR (crRNA) guides used in this study

pax7a.KI	TTTGTATGGTCAGATTCAGTGUUUUAGAGCUAUGCU
smyhc1.GFP.KI	AACAAACAAACUCGUUAUACGUUUUAGAGCUAUGCU
hbegfa.1	GAGCUACCGUUACUCACCGAGUUUUAGAGCUAUGCU
hbegfa.2	CGUCAACCUAUCAAACGUUUGUUUUAGAGCUAUGCU

Development • Supplementary information

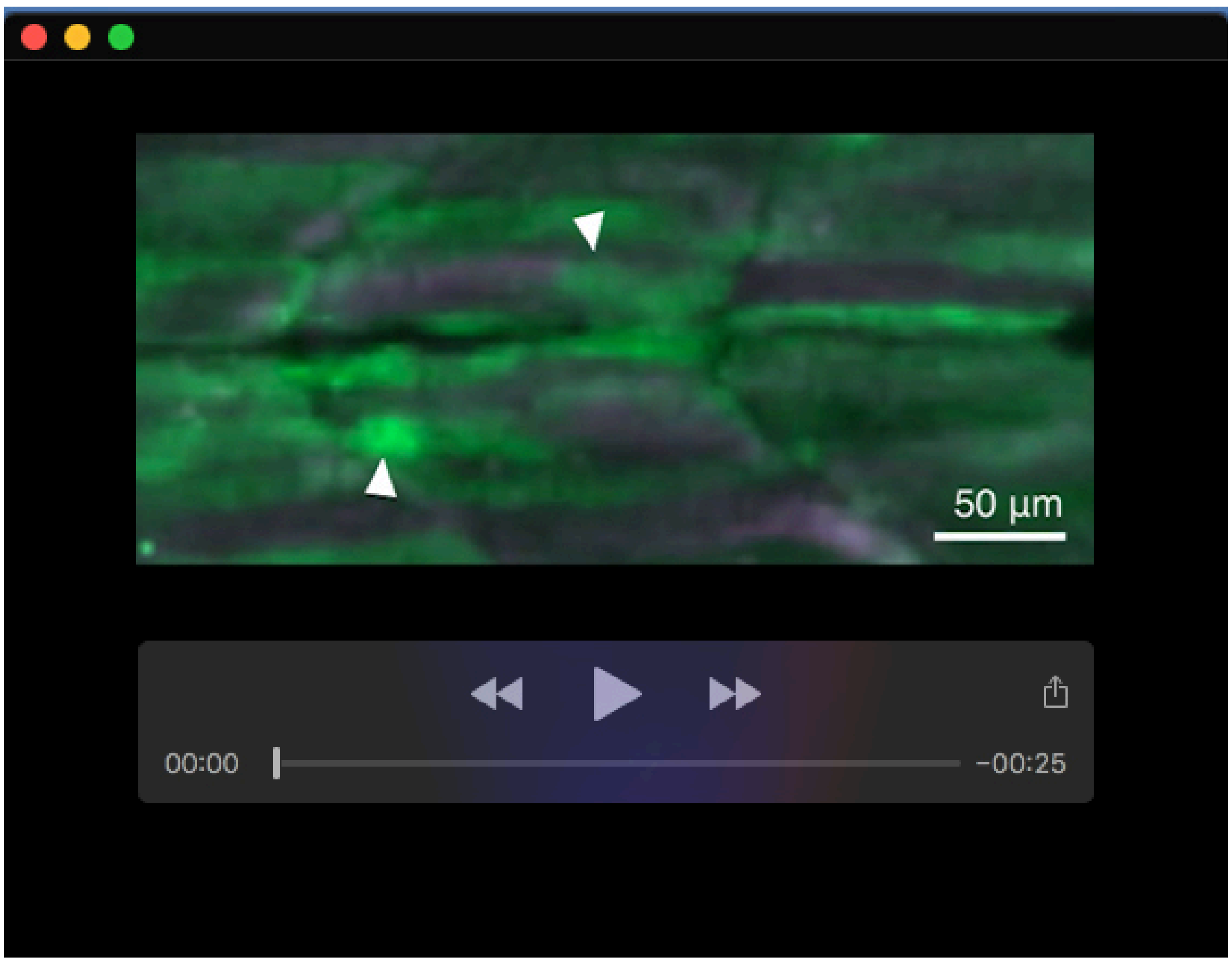

Movie 1. Live imaging of Tg(*actc1b*:NTR-2A-mCherry),(*-80.0myf5*:GFP) double transgenic larvae treated with MTZ for 12hr. Larvae was imaged from 12-24 hours post injury (5min/frame). Arrowheads show round GFP positive cells associated with a slow myofiber. As the round (presumably mononucleate) cells fade, the proximal myofibers gain GFP fluorescence that expands across the entire fiber over the imaging time course. These cellular events are similar to those reported for Tg(*pax7a*:GFP) larvae repairing needlestick muscle injuries (Pipalia et al., 2016).

Supplemental References

Pipalia, T. G., Koth, J., Roy, S. D., Hammond, C. L., Kawakami, K. and Hughes, S. M. (2016). Cellular dynamics of regeneration reveals role of two distinct Pax7 stem cell populations in larval zebrafish muscle repair. *Dis Model Mech* 9(6): 671-84.

Saraswathy, V. M., Zhou, L. and Mokalled, M. H. (2024). Single-cell analysis of innate spinal cord regeneration identifies intersecting modes of neuronal repair. *Nat Commun* 15(1): 6808.

Sur, Abhinav, Wang, Yiqun, Capar, Paulina, Margolin, Gennady, Prochaska, Morgan Kathleen and Farrell, Jeffrey A. (2023). Single-cell analysis of shared signatures and transcriptional diversity during zebrafish development. *Dev Cell* 58(24): 3028-3047.e12.
