# Supplementary material for: Hb-EGF directs systemic muscle repair
Source: Development. 2026 Mar 23;153(6):dev204993. doi: 10.1242/dev.204993 (PMC13495789; doi:10.1242/dev.204993)
Supplement: Supplementary file 2 [file develop-153-204993_review_history.pdf]

Hb-EGF directs systemic muscle repair

Haley C. Dean, Vishnu M. Saraswathy, Amulya Saini, Sibani Nachadalingam, Jennifer McAdow, Amruta Tendolkar, Mayssa H. Mokalled and Aaron N. Johnson

DOI: 10.1242/dev.204993

Editor: James Briscoe

Review timeline

Original submission:	3 June 2025
Editorial decision:	21 July 2025
First revision received:	28 November 2025
Editorial decision:	5 January 2026
Second revision received:	9 February 2026
Accepted:	10 February 2026

Original submission

First decision letter

MS ID#: dev.204993

MS TITLE: Hb-EGF directs systemic muscle repair

AUTHORS: Haley C. Dean, Vishnu Saraswathy, Amulya Saini, Sibani Nachadalingam, Jennifer McAdow, Amruta Tendolkar, Mayssa H. Mokalled and Aaron Johnson

Dear Dr Johnson,

I have now received all the referees' reports on the above manuscript, and have reached a decision. The referees' comments are appended below, or you can access them online: please go to:

As you will see, the referees express considerable interest in your work, but have some significant criticisms and recommend a substantial revision of your manuscript before we can consider publication. If you are able to revise the manuscript along the lines suggested, which may involve further experiments, I will be happy to receive a revised version of the manuscript. Your revised paper will be re-reviewed by one or more of the original referees, and acceptance of your manuscript will depend on your addressing satisfactorily the reviewers' major concerns. Please also note that Development will normally permit only one round of major revision. If it would be helpful, you are welcome to contact us to discuss your revision in greater detail.

Please attend to all of the reviewers' comments and ensure that you clearly highlight all changes made in the revised manuscript. Please avoid using 'Tracked changes' in Word files as these are lost in PDF conversion. I should be grateful if you would also provide a point-by-point response detailing how you have dealt with the points raised by the reviewers in the 'Response to Reviewers' box. If you do not agree with any of their criticisms or suggestions please explain clearly why this is so.
I strongly encourage you to pay attention to the following points:
Rev 1: Better contextualize your findings and cite the appropriate literature when required.
Rev 1: Provide clearer images when required
Rev 2: Address the potential point on toxicity
Rev 2: Address the issue on systemic muscle injury.
Rev 3: Acknowledge and discuss the alternative explanation

© 2026. Published by The Company of Biologists under the terms of the Creative Commons Attribution License (https://creativecommons.org/licenses/by/4.0/).

Reviewer 1

Advance summary and potential significance to field

Dean and Saraswathy et al. present a large-scale muscle injury model using a nitroreductase (NTR)-based system to investigate the genetic programs underlying muscle regeneration in zebrafish. Through lineage tracing, they show that systemic muscle injury is primarily repaired via myofiber fusion rather than de novo myofiber formation. Using single-cell RNA sequencing, the authors compare regenerative responses between systemic (NTR-based ablation) and local (needle stab) injuries, revealing distinct transcriptional programs associated with each injury type. Their functional analysis suggests that periderm-derived Hb-EGF is specifically required for regeneration following systemic, but not local, injury. Overall, the manuscript addresses an important question in regenerative biology and will be of broad interest to the Development readership. However, several concerns detailed below temper my enthusiasm.

Comments for the author

Major comments:

The authors use a nitroreductase-based system to induce systemic muscle injury. In other contexts, the MTZ-NTR system is commonly used to ablate cells by inducing apoptosis. However, in this study, it is not entirely clear whether the MTZ treatment leads to actual muscle cell death, muscle cell damage, or both. The reduction in mCherry expression shown in Fig 1D appears to suggest muscle cell ablation (i.e., loss of muscle cells), whereas results from Fig S2A argue that muscle cells do not die following MTZ treatment. The lack of obvious change in slow myofibers after ablation using a pan-muscle NTR line in Fig S1B raises similar questions: are slow myofibers only damaged, not ablated? The authors should provide stronger evidence to validate their muscle injury model, as this is critical for readers to accurately interpret the subsequent findings.

The authors propose that fusion to surviving myofibers is the predominant mechanism of muscle repair. However, could myoblasts initially generate de novo myofibers that later fuse with surviving fibers? This might explain why RFP+GFP- fibers appear more transient compared to GFP+ fibers. Would it be possible to use time-lapse imaging to directly visualize fusion events to strengthen the conclusions from the lineage tracing experiments?

The authors suggest that there are two populations of MuSCs: En1+ MuSCs in the ECL and Pax7+ interstitial MuSCs. However, these two populations are not clearly defined. According to the literature and as shown in Fig 3D-E, both ECL and interstitial MuSCs express Pax7. It is unclear whether the authors quantified all Pax7+ MuSCs or only the interstitial Pax7+ subset in Fig 6A-B. Additionally, the pattern of Pax7+ cells observed at the surface in Fig 6A, presumably representing ECL MuSCs, appears quite different from the En1+ MuSCs shown in Fig S6, which resemble superficial slow muscle fibers. Double labeling with Pax7, En1, and slow fiber markers would help clarify the identity and localization of these MuSC populations.

There is a substantial body of prior research examining muscle regeneration in zebrafish, yet only two papers from the Currie lab (Gurevich Science 2016; Ratnayake Nature 2021) are cited. For example, relevant studies include Seger Dev Dyn 2011; Knappe Front. Aging Neurosci. 2015; Pipalia Dis Model Mech 2016; Berberoglu Dev Biol 2017; and Sharma Development 2019. Several of these papers (e.g., Pipalia Dis Model Mech 2016 and Sharma Development 2019) address related questions such as muscle fusion versus de novo myofiber formation during regeneration. In addition, Berberoglu Dev Biol 2017 demonstrates that regenerating myofibers in zebrafish lack centralized nuclei—a point relevant to the current study. I encourage the authors to revise the introduction and discussion to better contextualize their work within this broader body of literature.

Although I appreciate images of the entire fish, it is difficult to visualize MuSCs or appreciate changes at the cellular level. Many figures would benefit from additional zoomed-in views and clearer annotations to highlight the relevant cells or structures. Additionally, in figures where

© 2026. Published by The Company of Biologists under the terms of the Creative Commons Attribution License (https://creativecommons.org/licenses/by/4.0/).

images and graphs are combined within a single panel, it would be clearer to separate them into different panels. This would make it easier to reference specific data or graph in the text.

Minor comments:

Page 5, "Regenerating myofibers in mammals are easily distinguished by the presence of centralized myonuclei, but we did not observe centralized nuclei in myofibers following systemic injury.": Fig 1D does not provide sufficient resolution to assess the localization of myonuclei. Please include appropriate references to support the statement regarding mammalian regeneration, and comment on prior zebrafish work (Berberoglu Dev Biol 2017), which reported similar findings.

Fig 1D: Why is there no MyHC staining in preinjured fish at 4 dpf? The MyHC aggregation phenotype and its cortical localization are not very clear. Higher magnification images are needed to better visualize these phenotypes.

Fig 1E: It appears that higher myf5:GFP expression is observed in newly regenerated muscles rather than in "activated MuSCs and proliferating muscle progenitors", as suggested. High magnification images are needed to visualize activated MuSCs.

Fig 1F: The image showed does not provide sufficient resolution to support the statement that "Pax7 is expressed in quiescent and activated MuSCs". It is not clear why the fish is labeled as "pax7aGFP,2A,NTR2.0 (-)". Are they positive for the reporter but not treated with MTZ? The genotype of the control should be clearly stated. Given that pax7 is expressed not only in MuSCs but also in cells in the brain, spinal cord, and neural crest lineage, it is possible that the observed reduction in survival following ablation is caused by loss of other pax7+ cells rather than impaired muscle regeneration.

Fig S1 legend: C should be B.

Page 6: The lineage trace reporter "Tg(ubb3:RFP.loxP.STOP.loxP.GFP)" should be ubb:LOXP-RFP-STOP-LOXP-GFP.

Fig 2A: To achieve removal of the RFP cassette, the loxP sites should be in the same orientation. There should be only one loxP left after recombination. Should "nRFP" in non-muscle cells be labeled as "RFP"?

Fig 2D: Is the RFP channel shown? It needs to be clearly labeled.

Fig 3: For the scRNA-seq data, it may be helpful to visualize dataset stratified by time point and experimental conditions. This approach would make the emergence of new cell clusters, such as activated MuSCs following injury, more apparent and easier to interpret.

Fig 3E: It is labeled as pax7b, but the text says pax7a (page 7).

Fig 3F: Why is the percentage of activated MuSCs in uninjured fish comparable to that in systemically injured fish at 1 dpi? Additionally, in the uninjured condition, why is the proportion of activated MuSCs higher at 1 dpi than at 2 and 4 dpi?

Fig S3B: Six clusters are shown, but the legend reads "Three ambiguous clusters". "ident genes" should be "identity genes"? There is not sufficient information in the figure legends. It is unclear which cell types were assigned to each ambiguous cluster (e.g., Cluster 1).

Fig 4: Panel C label is missing.

The sclerotome, a part of the somite, is a transient embryonic structure. By 4 dpf and beyond, it is unlikely that any sclerotome tissue remains.

© 2026. Published by The Company of Biologists under the terms of the Creative Commons Attribution License (https://creativecommons.org/licenses/by/4.0/).

Fig 4D and S4B: The predicted cell-cell interactions and their directionality under the systemic injury condition are difficult to see from the chord diagrams. It may be helpful to provide additional diagrams that specifically highlight interactions involving MuSCs to improve clarity.

Fig 5B: The authors state that "hbegfa was not expressed in Myf5+ MuSCs or muscle progenitors in response to systemic injury." However, Myf5+ MuSCs are not clearly indicated in Fig 5B. hbegfa appears to be expressed in some myf5:GFP cells at the surface, as well as in other cells located more medially, suggesting that its expression may not be restricted to the periderm, as claimed. Co-staining with a periderm-specific marker and other MuSC markers would help clarify the identity of the hbegfa-expressing cells.

Fig 5B-C: Labeling anatomical structures such as the spinal cord or notochord would help orient readers and clarify which region of the fish is being shown.

Page 9, "Incredibly, hbegfa was not induced at the local injury site but showed trending enrichment in the analogous position in response to systemic injury (Fig. 5C).": The image presented (lateral, systemic 2 dpi) does not appear to reflect what is shown in the graph on the right, which is not statistically significant. Describing this as a "trending enrichment" does not provide sufficient evidence for induced expression. Increasing the sample size may help clarify this issue. I suggest removing "Incredibly," especially given the lack of statistical significance in the observed changes. In addition, is hbegfa expression induced in the spinal cord (dorsal, local 2 dpi)? If so, does it mean that the needle stab also injures the spinal cord? This needs to be clarified in the text.

Fig 5D: The fish should be clearly labeled as "hbegfa crispants" to avoid confusion with hbegfa in situ staining. Are the image panels on the right zoomed-in views? This should be properly labeled in the figure and noted in the legend. Quantification of the observed effects is also needed to support the interpretation. What does the arrow point at?

Fig S5: Are the images in grey scale Smyhc1:GFP?

Fig 6A: Are the images in grey scale Pax7 staining?

Fig 6B, bottom graph: The non-significance might be due to small sample size (n=4) and high variability. Increasing the n number might help to clarify this.

Fig. 6D: Do hbegfa mutants have a similar number of myofibers as wild-type fish in the absence of injury? Without this information, it is difficult to determine whether the observed reduction in myofiber number in mutants results from a regeneration defect or reflects a developmental phenotype.

Fig 6E: The "pax7aNTR,2A,GFP" and "pax7aGFP" labels in the image need to be corrected so it is consistent with the rest of the paper.

Fig 6E-F, "% of fish with GFP+ cells": Does this refer to any GFP+ cells, or specifically GFP+ MuSCs? In the image shown in Fig 6E, the GFP+ cells (arrowheads) look like pigment cells, which are known to express Pax7, rather than MuSCs. Additionally, if all the fish carry the pax7aGFP,2A,NTR2.0 reporter, why are GFP+ MuSCs absent in the control image, and why do only 15% of control fish exhibit any GFP+ cells? High magnification images are needed to visualize Pax7+ MuSCs.

Fig S6A: The figure should be labeled as En1 to be consistent. The image needs to be labeled as "hbegfa crispant" for clarity. Are the images in grey scale En1 staining?

Fig S6: The title "Hb-EGF does not En1+ MuSCs or local muscle repair." needs to be corrected.

It is uncommon to write the genotype as actc1b:NTR,2A,mCherry instead of actc1b:NTR-2A-mCherry. I recommend using the standard nomenclature outlined by ZFIN for consistency and clarity.

Please define acronym "dpi" at first use.

© 2026. Published by The Company of Biologists under the terms of the Creative Commons Attribution License (https://creativecommons.org/licenses/by/4.0/).

The sample size (n) is not consistently reported in the figure legends.

Scale bars: µM should be µm.

In many images, the blue channel is not clearly labeled. Is this DAPI staining?

Reviewer 2

Advance summary and potential significance to field

The manuscript "Hb-EGF directs systemic muscle repair" by Dean et al., attempt to define the major regenerative events in a systemic muscle injury, and compare them to those happening after a local muscle injury. They use sc-RNA seq to find essential signalling pathways that define the systemic injury and describe and test Hb-EGF as such.
This is a well written manuscript making good use of many new lines created by CRISPR/Cas9 knock-ins and transgenesis, crispant etc. It also shows a methodically explained use of sc-RNA and the existing scSeq atlas. However, there are many strong statements on the cellular basis of regeneration post- systemic injury that lack in proof and some issues that need to be addressed before publication in Development as detailed below.
Major points:
1. In the results section: An inducible injury model to investigate systemic muscle repair, the authors write: "We treated 4dpf Tg(actc1b:NTR,2A,mCherry) larvae with a range of MTZ concentrations and incubation times and found 12hr treatment of 5mM MTZ drastically reduced swim function at 2dpi, but strikingly the injured larvae regained full swim function by 12dpi (Fig.1A,B)"
Previous use of NTR lines have shown in some cases various degrees of toxicity and a new improved NTR2.0 system was created (Sharrock et al., 2022, PMID: 35132245). It is not clear to me what NTR system is being used here. It is necessary to assess if only muscle fibres are damaged/ablated or that other tissues are also affected under the experimental regime selected by the authors. This is especially important if the first-generation NTR system was used. (In that regard, pax7aGFP,2A,NTR2.0 may have a direct effect on the brain due to pax7 expression)

2. The damage to the muscle is described very briefly and should be elaborated further; what happens to the sarcomeres?. MyHC only shown as a cross section (Figs.1D and 5D for example). A high magnification image of fibres with MyHC and/or other sarcomeric proteins in a lateral view would be beneficial to understand the cellular events in the systemic injury.

3. In addition to point 2, in the results section"Systemic regeneration repairs surviving myofibers", the authors claim that "Systemic repair therefore does not create new myofibers" and that "The absence of smyhc1sfgfp expression in fast myofibers during systemic repair and the persistence of GFP expression in lineage traced myofibers after injury argue the primary cellular mechanism of systemic muscle repair is fusion-based repair of surviving myofibers.". What happens then during the systemic injury to muscle nuclei?,is there any nuclear apoptosis and fusion? If not is the only change is in the MuSC state? The authors say : "Injured larvae transgenic for Tg(actc1b:NTR,2A,mCherry) and Tg(myf5:GFP) activated GFP expression at 2dpi, suggesting systemic repair is a MuSC-based process (Fig. 1E)." The regeneration however, could also be re-activation of muscle genes like myf5 in the existing myofiber nuclei and not in the MuSC. The manuscript needs some insights into these questions and the authors should rephrase their strong conclusions throughout unless they provide enough evidence to support fusion-based system. This can be done for example by use of DAPI stain, nuclear transgenics or other reagents and by looking into upregulation of fusion genes such as myomixer or jamb/jamc etc.)

Minor points

4. The authors say: "slow myosin heavy chain 1 (smyhc1) is expressed in newly created fast myofibers after local muscle injury in larvae (Gurevich et al., 2016) and say " Systemic repair therefore does not create new myofibers."The authors quote Gurevic et al. 2016 that used the Tg

© 2026. Published by The Company of Biologists under the terms of the Creative Commons Attribution License (https://creativecommons.org/licenses/by/4.0/).

(smyhc1:gfp) but use a novel knock-in line here. Does the knock-in show the same effect of transient activation in fast cells after local injury?

5. In Fig1F, actc1b:NTR,2A, mCherry shown only with Mtz. Where is 'no Mtz' control and how did the statistics done for this pair then?

6. "Uninjured smyhc1sfgfp larvae expressed GFP in the superficial slow myofibers of the truck musculature but not in the deep fast myofibers (Fig.S1A,B)." Should say trunk
7. Figure S6. Hb-EGF does not En1+ MuSCs or local muscle repair -nonsensical header. Please correct.
8. In the Discussion: "Hb-EGF is produced by both macrophages and endothelial __in the liver after ischemia injury and is required for hepatocyte proliferation during liver regeneration(Wu et al., 2024; Yang et al., 2024)."-Should be 'endothelial cells'

<u>Reviewer 3</u>

Advance summary and potential significance to field

The manuscript by Dean, Saraswathy and colleagues describes development of a new nitroreductase-based systemic skeletal muscle injury model and extensive comparison of this new model to the "standard" transection. They identify Hb-EGF signaling as uniquely induced in response to systemic muscle injury and necessary for repair after systemic injury. Along the way, authors generated and characterized five new transgenic lines: three by transposon transgenesis and two by knock-in.

Major concern:
In multiple places throughout the manuscript authors argue that the differences in response are primarily attributable to the scale of the injury. This may very well be the case. However, there is an alternative explanation: different modes of injury - targeted death of cardiomyocytes versus physical injury inevitably immediately affecting many different cell types - are likely to induce at least somewhat different responses. There does not seem to be a straightforward way to unequivocally rule this alternative explanation out. This has to be clearly acknowledged.

Minor concerns:
1. Please Clarify if generated and used Tg(actc1b:NTR,2A,mCherry), Tg(actc1b:NTR,2A) and Tg(actc1b:CreERT2) were single-copy by Mendelian segregation, or if multicopy fish were used. It appears that in each case, progeny of two independent founders were used. It would be good to let the readers know which founder was used for which experiments. Authors make it clear that the main issue when generating Tg(actc1b:CreERT2) line(s) was low recombination activity. Was expression of Tg(actc1b:NTR,2A,mCherry) and Tg(actc1b:NTR,2A) reproducible between founders, or was it highly subject to position effects?
2. Please provide a reference for the reporter Tg(ubb3:RFP.loxP.STOP.loxP.GFP) lineage tracing reporter (page 7, paragraph 3). The Mosimann et al 2011 paper cited for Tg(ubb:LOXP-RFP-STOP-LOXP-GFP) reporter on page 16 only describes ubiquitous green-to-red reporter Tg(ubi:loxP-GFP-loxP-mCherry). Please also reconcile if Tg(ubb3:RFP.loxP.STOP.loxP.GFP) (on page 7 and Tg(ubb:LOXP-RFP-STOP-LOXP-GFP) on page 16 describe the same construct.

First revision

<u>Author response to reviewers' comments</u>

We greatly appreciate all of the thoughtful and insightful reviewer comments. We have been hard at work addressing these comments experimentally, which has greatly improved the manuscript overall. Thank you for the feedback.

© 2026. Published by The Company of Biologists under the terms of the Creative Commons Attribution License (https://creativecommons.org/licenses/by/4.0/).

Reviewer 1

Comment: Overall, the manuscript addresses an important question in regenerative biology and will be of broad interest to the Development readership.

Response: Thank you for your interest and enthusiasm in our work!!!

Comment: The authors use a nitroreductase-based system to induce systemic muscle injury. In other contexts, the MTZ-NTR system is commonly used to ablate cells by inducing apoptosis. However, in this study, it is not entirely clear whether the MTZ treatment leads to actual muscle cell death, muscle cell damage, or both. The reduction in mCherry expression shown in Fig 1D appears to suggest muscle cell ablation (i.e., loss of muscle cells), whereas results from Fig S2A argue that muscle cells do not die following MTZ treatment. The lack of obvious change in slow myofibers after ablation using a pan-muscle NTR line in Fig S1B raises similar questions: are slow myofibers only damaged, not ablated? The authors should provide stronger evidence to validate their muscle injury model, as this is critical for readers to accurately interpret the subsequent findings.

Response: Our apologies if the survivability of myofibers was not clear. We had quantified the number of surviving myofibers by lineage trace, which showed no significant change between injured and uninjured larvae (Fig. 2D). In response to the reviewer's suggestion, we used a cleaved Caspase 3 antibody to assay apoptosis and found a few myofibers die in response to MTZ treatment (Fig. S2A, see below). By comparison, needlestick injury caused 300% more myofiber apoptosis than MTZ injury (Fig. S2A). We also quantified Smyhc1.GFP+ cells in transverse sections and found a significant decrease in the number of GFP+ cells at 1- and 2-dpi (Fig. S2C). However, we did not observe cleaved Caspase 3 expression in slow myofibers (Fig. S2A, see below), arguing the injury-induced changes in Smyhc1.GFP expression are due to protein mislocalization (perhaps from sarcomere disassembly). We agree that MTZ injury reduced mCherry expression in myofibers, but found mCherry expression was undetectable in apoptotic myofibers from needlestick injury (Fig. S2A, see below). These experiments show a majority of myofibers survive MTZ-NTR injury at the dose used in this study.

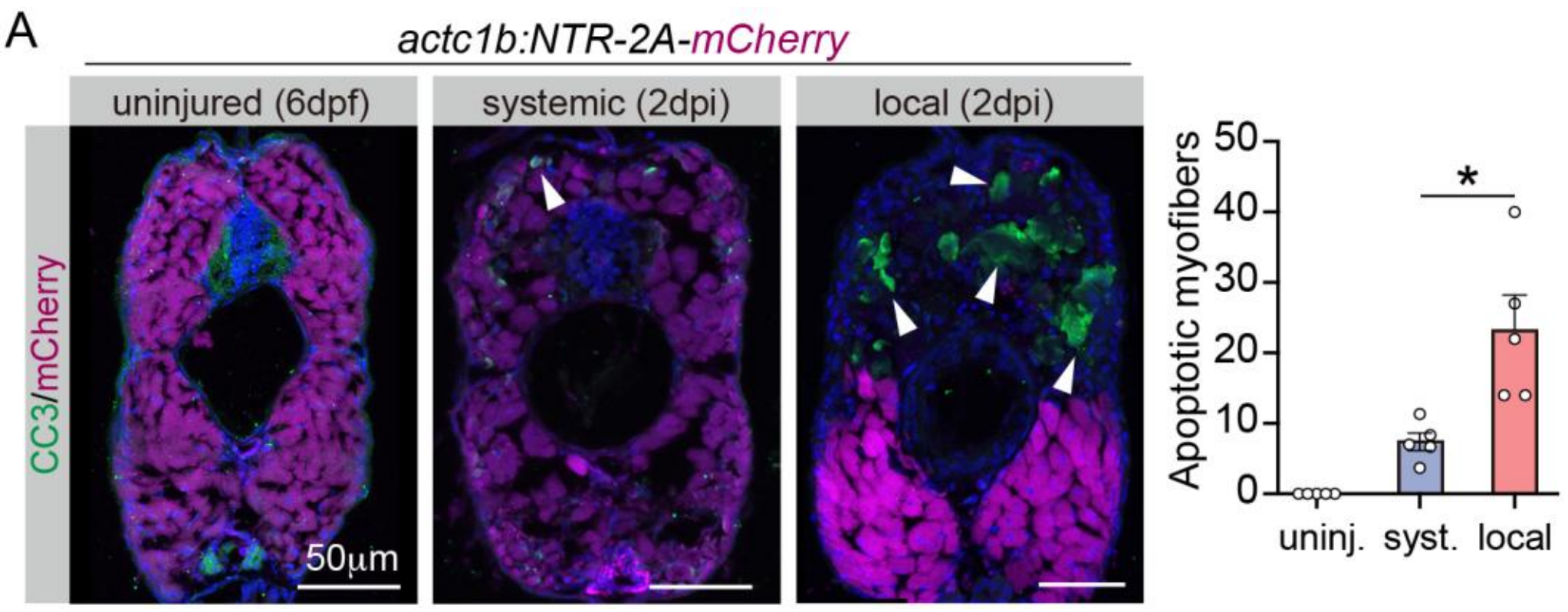

Comment: The authors propose that fusion to surviving myofibers is the predominant mechanism of muscle repair. However, could myoblasts initially generate de novo myofibers that later fuse with surviving fibers? This might explain why RFP+GFP- fibers appear more transient compared to GFP+ fibers. Would it be possible to use time-lapse imaging to directly visualize fusion events to strengthen the conclusions from the lineage tracing experiments?

Response: This is an interesting point. Although we have shown myotube-myotube fusion is possible in some mutant backgrounds during Drosophila myogenesis, this is an extremely uncommon event in vivo (and we are not sure it is even possible after maturation into myofibers). We live imaged myf5:GFP for 24hpi and found evidence for fusion events (Movie 1) that recapitulate previous reports of pax7a:GFP fusion (Pipalia Dis Model Mech 2016).

With respect to the lineage trace, the transient nature of the GFP- (or actually low-GFP expressing) myofibers in the lineage trace is likely due to mononucleate myoblasts fusing into

© 2026. Published by The Company of Biologists under the terms of the Creative Commons Attribution License (https://creativecommons.org/licenses/by/4.0/).

surviving fibers and transiently diluting GFP expression (the MuSC derived myoblasts presumably don't express GFP).

Comment: The authors suggest that there are two populations of MuSCs: En1+ MuSCs in the ECL and Pax7+ interstitial MuSCs. However, these two populations are not clearly defined. According to the literature and as shown in Fig 3D-E, both ECL and interstitial MuSCs express Pax7. It is unclear whether the authors quantified all Pax7+ MuSCs or only the interstitial Pax7+ subset in Fig 6A-B. Additionally, the pattern of Pax7+ cells observed at the surface in Fig 6A, presumably representing ECL MuSCs, appears quite different from the En1+ MuSCs shown in Fig S6, which resemble superficial slow muscle fibers. Double labeling with Pax7, En1, and slow fiber markers would help clarify the identity and localization of these MuSC populations.

Response: Thank you for pointing this out. We assayed all Pax7+ cells in Fig. 6A-B and have pointed this out in the revised manuscript. Double labeling with Pax7 and En1 antibodies was not possible because the antibodies that work in fish were both made in mouse. We crossed En1.Gal4, UAS.RFP fish with our smych1^{GFP} fish and labeled transverse sections with Pax7, GFP, and RFP antibodies. Although (En1.Gal4) RFP was not expressed as broadly as En1 protein in the ECL, a majority of RFP+ cells expressed Pax7 (Fig. S6A; see below). Importantly, Smych1.GFP slow muscle fibers did not express RFP or Pax7. According to our scSeq data (Fig. 3C-E), *pax7b* is not expressed in all *en1b*+ MuSCs and, as the reviewer points out, the Pax7 antibody does not react with all of the ECL cells recognized by the En1 antibody. Thus, there is molecular diversity among the En1 expressing MuSCs which we highlight in the revised manuscript.

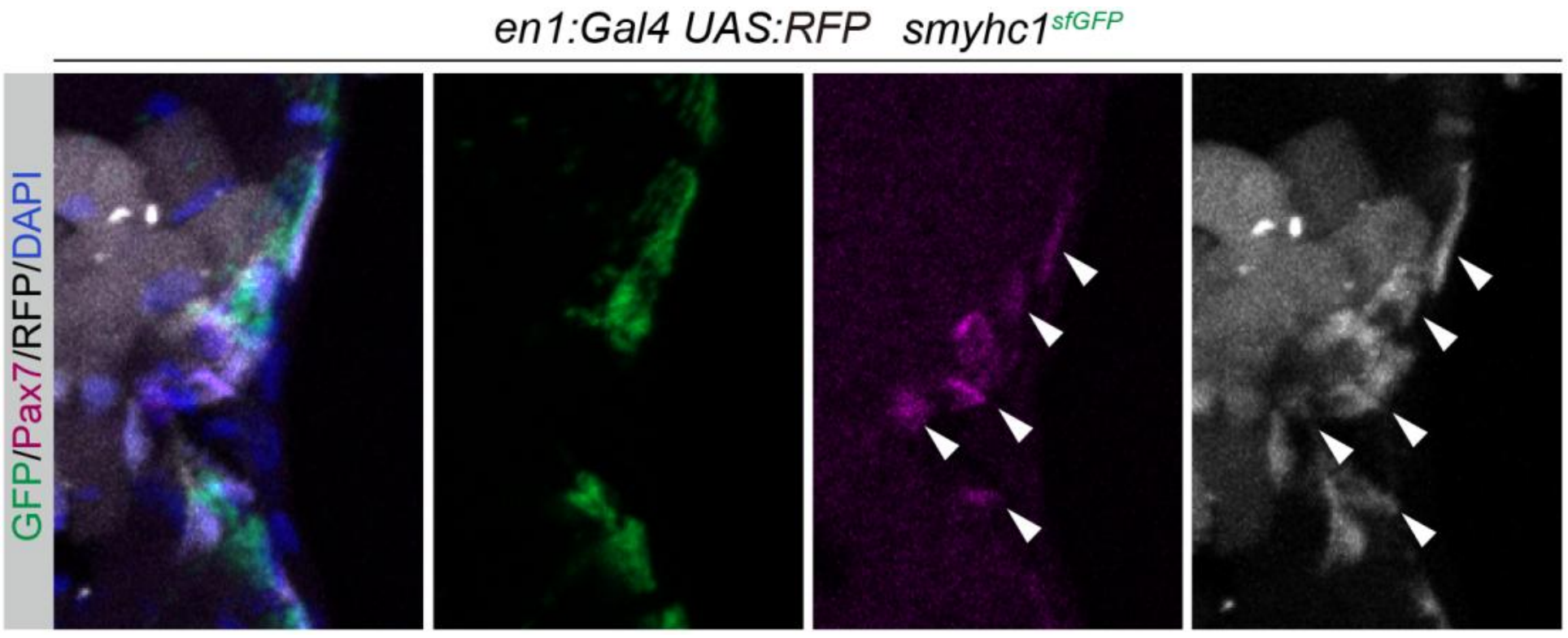

Comment: There is a substantial body of prior research examining muscle regeneration in zebrafish, yet only two papers from the Currie lab (Gurevich Science 2016; Ratnayake Nature 2021) are cited. For example, relevant studies include Seger Dev Dyn 2011; Knappe Front. Aging Neurosci. 2015; Pipalia Dis Model Mech 2016; Berberoglu Dev Biol 2017; and Sharma Development 2019. Several of these papers (e.g., Pipalia Dis Model Mech 2016 and Sharma Development 2019) address related questions such as muscle fusion versus de novo myofiber formation during regeneration. In addition, Berberoglu Dev Biol 2017 demonstrates that regenerating myofibers in zebrafish lack centralized nuclei—a point relevant to the current study. I encourage the authors to revise the introduction and discussion to better contextualize their work within this broader body of literature.

Response: Thank you for making us aware of the larger canon of fish regeneration papers. We have included the mentioned references where appropriate the Intro, Results, and Discussion.

Comment: Although I appreciate images of the entire fish, it is difficult to visualize MuSCs or appreciate changes at the cellular level. Many figures would benefit from additional zoomed-in views and clearer annotations to highlight the relevant cells or structures. Additionally, in figures where images and graphs are combined within a single panel, it would be clearer to separate them into different panels. This would make it easier to reference specific data or graph in the text.

Response: Great suggestions. Done!

© 2026. Published by The Company of Biologists under the terms of the Creative Commons Attribution License (https://creativecommons.org/licenses/by/4.0/).

Minor comments:

Comment: Page 5, "Regenerating myofibers in mammals are easily distinguished by the presence of centralized myonuclei, but we did not observe centralized nuclei in myofibers following systemic injury.": Fig 1D does not provide sufficient resolution to assess the localization of myonuclei. Please include appropriate references to support the statement regarding mammalian regeneration, and comment on prior zebrafish work (Berberoglu Dev Biol 2017), which reported similar findings.

Response: Thanks for these insights. After a more careful examination of our histology, we were able to identify centralized nuclei in injured fish. We also found centralized nuclei in uninjured larvae, which likely reflects myofiber growth (or replacement as shown in Kumar 2024 EMBO).

Comment: Fig 1D: Why is there no MyHC staining in preinjured fish at 4 dpf? The MyHC aggregation phenotype and its cortical localization are not very clear. Higher magnification images are needed to better visualize these phenotypes.

Response: There is certainly MyHC in preinjured fish. The fluorescent signal from the MyHC antibody is so bright in injured fish that imaging at uniform levels among samples makes the uninjured MyHC signal dim. However, we prepared a new set of samples and re-imaged all timepoints in Fig. 1D (now 1E) to better show MyHC among timepoints and provided higher magnification images.

Comment: Fig 1E: It appears that higher myf5:GFP expression is observed in newly regenerated muscles rather than in "activated MuSCs and proliferating muscle progenitors", as suggested. High magnification images are needed to visualize activated MuSCs.

Response: Great point. We show high magnification images of myf5:GFP (now in Fig. 1C; see below) and provide live imaging data of myf5:GFP (Movie 1). We have been more careful with our language regarding myf5+ cells in the Results.

C

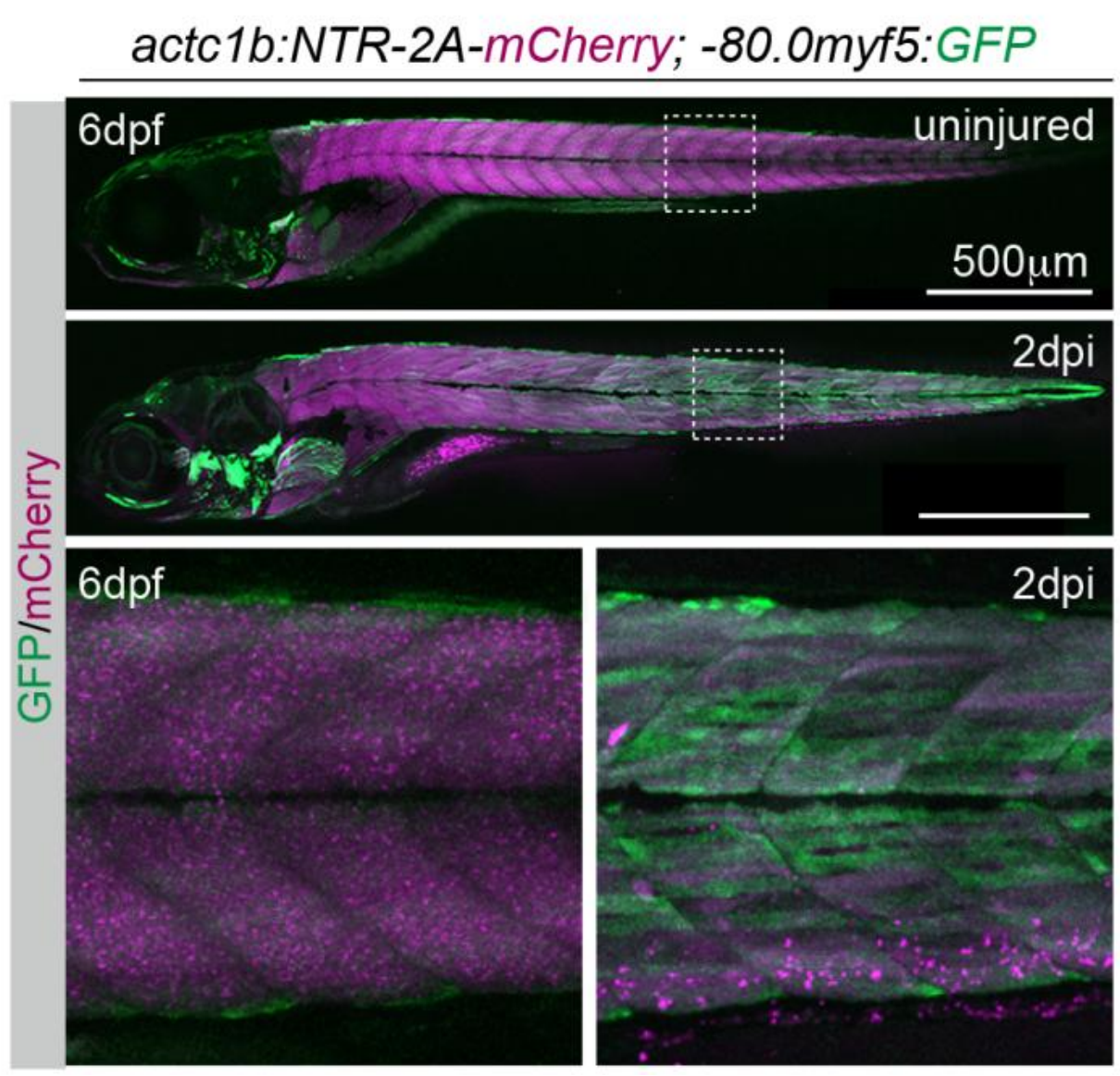

Comment: Fig 1F: The image showed does not provide sufficient resolution to support the statement that "Pax7 is expressed in quiescent and activated MuSCs". It is not clear why the fish is labeled as "pax7aGFP,2A,NTR2.0 (-)". Are they positive for the reporter but not treated with MTZ? The genotype of the control should be clearly stated. Given that pax7 is expressed not only in MuSCs but also in cells in the brain, spinal cord, and neural crest lineage, it is possible that the observed reduction in survival following ablation is caused by loss of other pax7+ cells rather than impaired muscle regeneration.

Response: We apologize for the confusion in our previous Fig. 1F (now S1E,F). The fish we show

© 2026. Published by The Company of Biologists under the terms of the Creative Commons Attribution License (https://creativecommons.org/licenses/by/4.0/).

have not been treated with MTZ, which we made clear in the revised figure (see below). This is a knock-in line, now correctly labeled, and the micrographs are meant to convey that GFP is visible in untreated animals, providing evidence that the line "works". The reviewer makes a great point about pax7 expression outside the muscle lineage. However, our survival curve shows treating pax7aGFP-2A-NTR fish with MTZ does not affect survival in the absence of muscle injury (light blue line in survival curve). Our interpretation is that Pax7a expressing cells are necessary for survival after muscle injury, but we cannot say for sure it is the MuSC population that contribute to survival. We have changed the text to reflect the reviewer's suggestion.

Our revised conclusion reads:

These data argue systemic muscle injury reduces motor function by disrupting the myofiber contractile machinery and that systemic repair requires *pax7a* expressing cells.

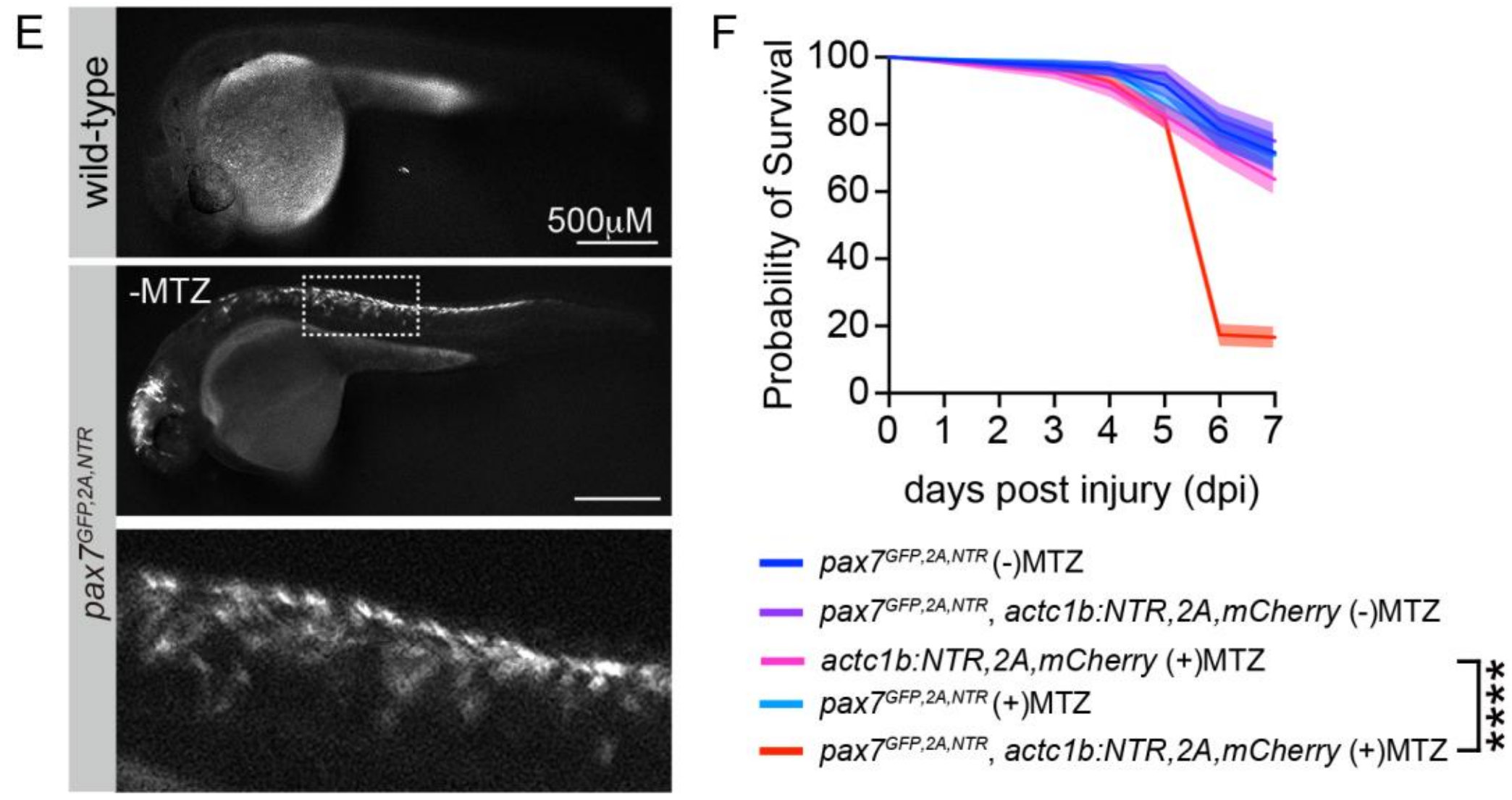

Comment: Fig S1 legend: C should be B.

Response: Thanks. Legend revised.

Comment: Page 6: The lineage trace reporter "Tg(ubb3:RFP.loxP.STOP.loxP.GFP)" should be ubb:LOXP-RFP-STOP-LOXP-GFP.

Response: Correct. Thanks!!!

Comment: Fig 2A: To achieve removal of the RFP cassette, the loxP sites should be in the same orientation. There should be only one loxP left after recombination. Should "nRFP" in non-muscle cells be labeled as "RFP"?

Response: Thanks. Corrections made.

Comment: Fig 2D: Is the RFP channel shown? It needs to be clearly labeled.

Response: Shown and labeled. Thanks!

Comment: Fig 3: For the scRNA-seq data, it may be helpful to visualize dataset stratified by time point and experimental conditions. This approach would make the emergence of new cell clusters, such as activated MuSCs following injury, more apparent and easier to interpret.

Response: We appreciate the reviewer's suggestion. Unfortunately, we did not observe any cell populations emerge in the overall analysis or in the muscle subcluster analysis (we looked very carefully). We feel showing fourteen UMAPs (seven for all cells and seven for the muscle subcluster) becomes too overwhelming to appreciate changes in cell populations. We show the

© 2026. Published by The Company of Biologists under the terms of the Creative Commons Attribution License (https://creativecommons.org/licenses/by/4.0/).

single bar graph in Fig. 3F because activated MuSCs were the only population to appreciable change among samples.

Comment: Fig 3E: It is labeled as pax7b, but the text says pax7a (page 7).

Response: Nice catch. Thanks!

Comment: Fig 3F: Why is the percentage of activated MuSCs in uninjured fish comparable to that in systemically injured fish at 1 dpi? Additionally, in the uninjured condition, why is the proportion of activated MuSCs higher at 1 dpi than at 2 and 4 dpi?

Response: It turns out this discrepancy at 1dpi was due to changes in the overall number of MuSCs among samples. We normalized to activated MuSCs to MuSCs in the samples, which made the proportions more logical among injury timepoints.

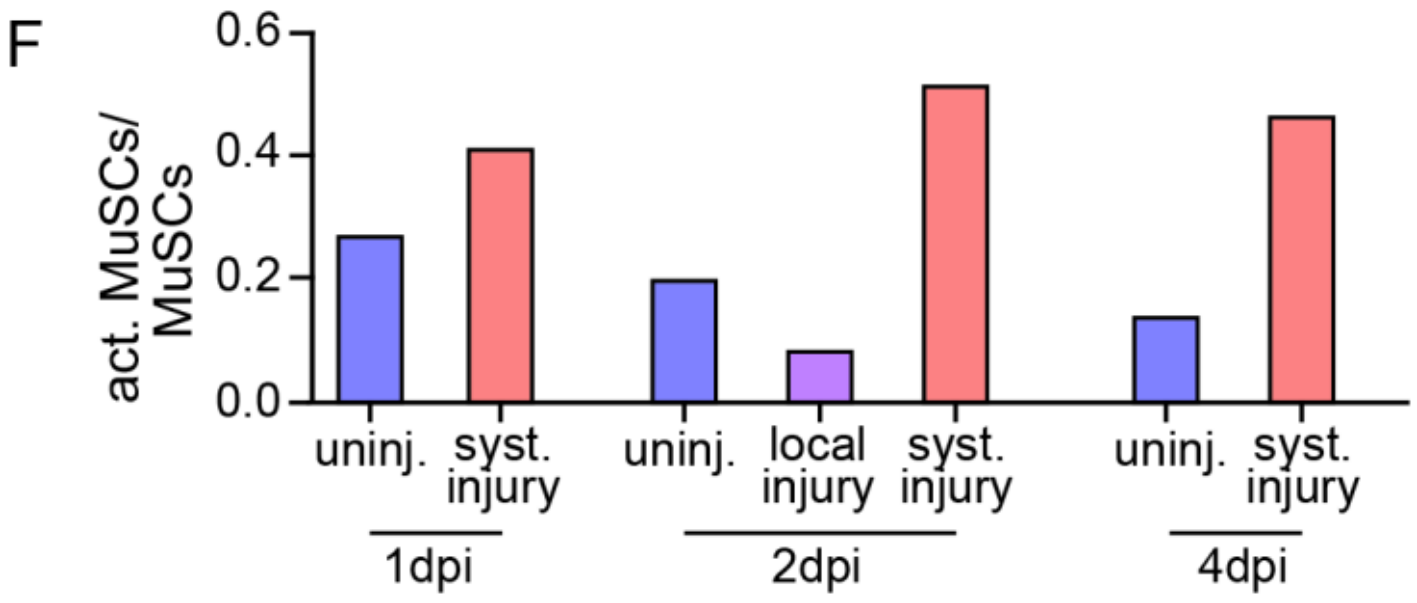

Comment: Fig S3B: Six clusters are shown, but the legend reads "Three ambiguous clusters". "ident genes" should be "identity genes"? There is not sufficient information in the figure legends. It is unclear which cell types were assigned to each ambiguous cluster (e.g., Cluster 1).

Response: Agreed. We rewrote the legend to clarify our approach and to highlight the cell types that required a little extra work to annotate.

Comment: Fig 4: Panel C label is missing.

Response: Nice catch. Thanks!

Comment: The sclerotome, a part of the somite, is a transient embryonic structure. By 4 dpf and beyond, it is unlikely that any sclerotome tissue remains.

Response: Great point. After some additional analysis of marker genes, it seems “myosepta” is the correct term. We have made the appropriate changes throughout the manuscript.

Comment: Fig 4D and S4B: The predicted cell-cell interactions and their directionality under the systemic injury condition are difficult to see from the chord diagrams. It may be helpful to provide additional diagrams that specifically highlight interactions involving MuSCs to improve clarity.

Response: We provided an additional chord diagram to clarify.

Comment: Fig 5B: The authors state that "hbegfa was not expressed in Myf5+ MuSCs or muscle progenitors in response to systemic injury." However, Myf5+ MuSCs are not clearly indicated in Fig 5B. hbegfa appears to be expressed in some myf5:GFP cells at the surface, as well as in other cells located more medially, suggesting that its expression may not be restricted to the periderm, as claimed. Co-staining with a periderm-specific marker and other MuSC markers would help clarify the identity of the hbegfa-expressing cells.

Response: Great suggestion. We identified a periderm marker from our scSeq (*frem2a*) and replaced the images in Fig. 5B with a double HCR that shows *hbegfa* is expressed in the periderm.

© 2026. Published by The Company of Biologists under the terms of the Creative Commons Attribution License (https://creativecommons.org/licenses/by/4.0/).

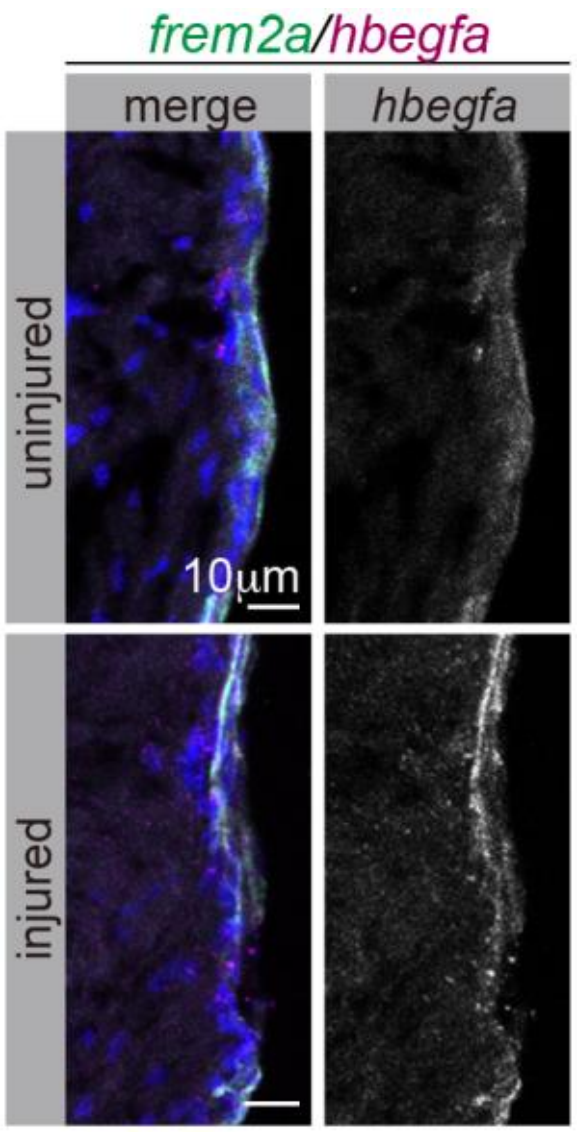

Comment: Fig 5B-C: Labeling anatomical structures such as the spinal cord or notochord would help orient readers and clarify which region of the fish is being shown.

Response: Great suggestion. Done!

Comment: Page 9, "Incredibly, hbegfa was not induced at the local injury site but showed trending enrichment in the analogous position in response to systemic injury (Fig. 5C).": The image presented (lateral, systemic 2 dpi) does not appear to reflect what is shown in the graph on the right, which is not statistically significant. Describing this as a "trending enrichment" does not provide sufficient evidence for induced expression. Increasing the sample size may help clarify this issue. I suggest removing "Incredibly," especially given the lack of statistical significance in the observed changes. In addition, is hbegfa expression induced in the spinal cord (dorsal, local 2 dpi)? If so, does it mean that the needle stab also injures the spinal cord? This needs to be clarified in the text.

Response: Perhaps it wasn't clear that we did observe a statistically significant increase in expression when assaying *hbegfa* signal across the entire transverse section. We have made that result more clear and revised our language accordingly.

Comment: Fig 5D: The fish should be clearly labeled as "hbegfa crispants" to avoid confusion with hbegfa in situ staining. Are the image panels on the right zoomed-in views? This should be properly labeled in the figure and noted in the legend. Quantification of the observed effects is also needed to support the interpretation. What does the arrow point at?

Response: Thanks for pointing this out. Changes have been made.

Comment: Fig S5: Are the images in grey scale Smyhc1:GFP?

Response: Yes. Clarified.

Comment: Fig 6A: Are the images in grey scale Pax7 staining?

Response: Yes. Clarified.

Comment: Fig 6B, bottom graph: The non-significance might be due to small sample size (n=4) and high variability. Increasing the n number might help to clarify this.

Response: Agreed. Variability was quite high. We repeated the quantification and combined the preinjury and injury timepoints in a new graph (Fig. 6B).

© 2026. Published by The Company of Biologists under the terms of the Creative Commons Attribution License (https://creativecommons.org/licenses/by/4.0/).

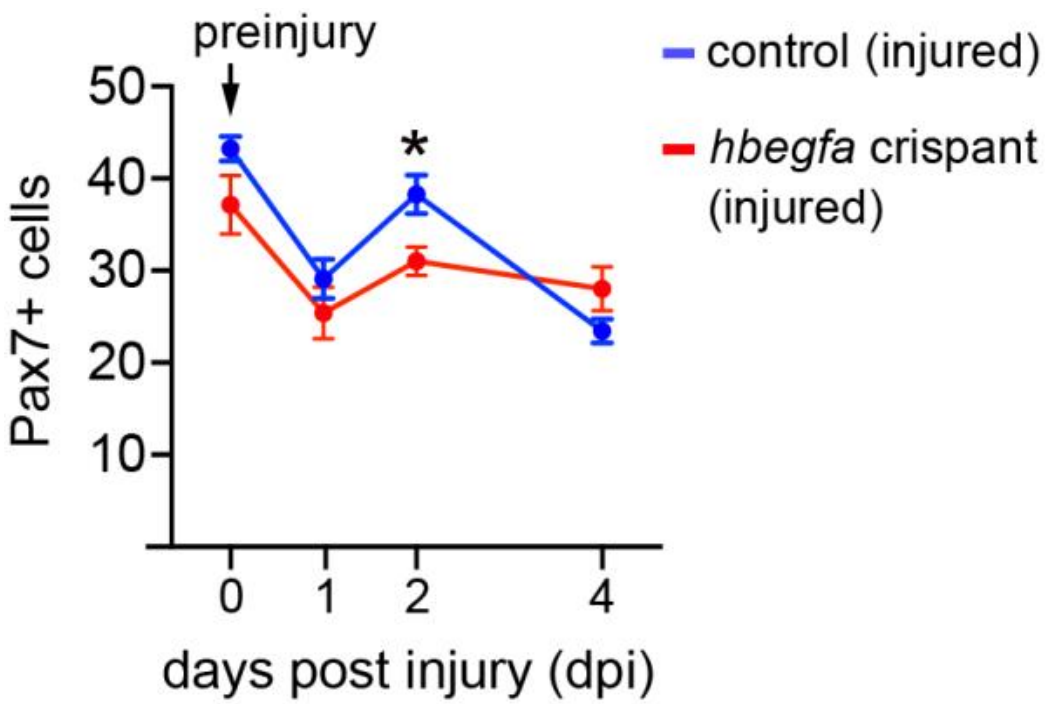

Comment: Fig. 6D: Do hbegfa mutants have a similar number of myofibers as wild-type fish in the absence of injury? Without this information, it is difficult to determine whether the observed reduction in myofiber number in mutants results from a regeneration defect or reflects a developmental phenotype.

Response: Good point. The number of myofibers is comparable between the genotypes in the absence of injury.

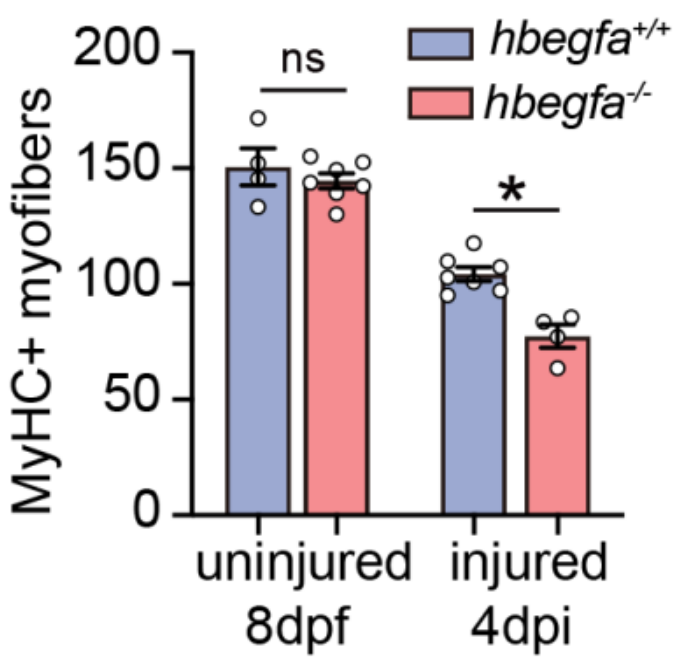

Comment: Fig 6E: The "pax7aNTR,2A,GFP" and "pax7aGFP" labels in the image need to be corrected so it is consistent with the rest of the paper.

Response: Corrected. Thanks.

Comment: Fig 6E-F, "% of fish with GFP+ cells": Does this refer to any GFP+ cells, or specifically GFP+ MuSCs? In the image shown in Fig 6E, the GFP+ cells (arrowheads) look like pigment cells, which are known to express Pax7, rather than MuSCs. Additionally, if all the fish carry the pax7aGFP,2A,NTR2.0 reporter, why are GFP+ MuSCs absent in the control image, and why do only 15% of control fish exhibit any GFP+ cells? High magnification images are needed to visualize Pax7+ MuSCs.

Response: Thanks for pointing this out. We repeated the Hb-EGF injections and used improved imaging methods to assess GFP+ cells. In these new experiments we were able to identify GFP+ cells in all fish and counted the total number of Pax7+ cells. The morphology of the cells we quantified are more consistent with MuSCs (see below), however we cannot say for certain that the cells are MuSCs. We have revised our text to clarify this point.

Revised text: Larvae injected with Hb-EGF showed a significant increase in the number of GFP+ cells, arguing Hb-EGF activates the proliferation of Pax7a expressing cells.

© 2026. Published by The Company of Biologists under the terms of the Creative Commons Attribution License (https://creativecommons.org/licenses/by/4.0/).

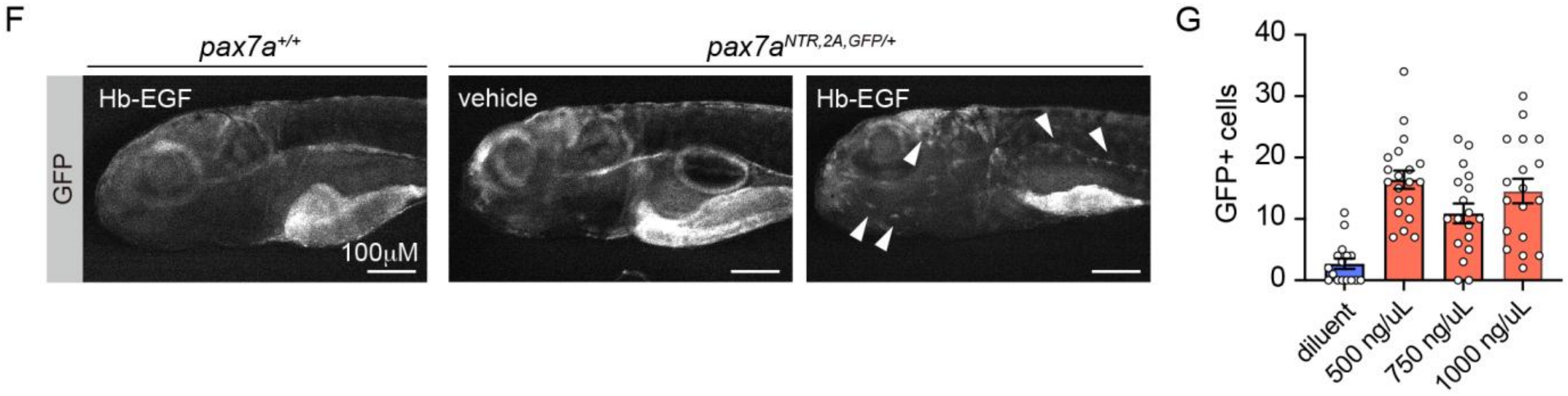

Comment: Fig S6A: The figure should be labeled as En1 to be consistent. The image needs to be labeled as "hbegfa crispant" for clarity. Are the images in grey scale En1 staining?

Response: Done.

Comment: Fig S6: The title "Hb-EGF does not En1+ MuSCs or local muscle repair." needs to be corrected.

Response: It sure does!! Done.

Comment: It is uncommon to write the genotype as actc1b:NTR,2A,mCherry instead of actc1b:NTR-2A-mCherry. I recommend using the standard nomenclature outlined by ZFIN for consistency and clarity.

Response: Thanks for pointing this out. Done.

Comment: Please define acronym "dpi" at first use.

Response: Done.

Comment: The sample size (n) is not consistently reported in the figure legends.

Response: Reported throughout.

Comment: Scale bars: μM should be μm.

Response: Yikes. Changed.

Comment: In many images, the blue channel is not clearly labeled. Is this DAPI staining?

Response: Yes. Fixed.

Reviewer 2:

Comment: This is a well written manuscript making good use of many new lines created by CRISPR/Cas9 knock-ins and transgenesis, crispant etc. It also shows a methodically explained use of sc-RNA and the existing scSeq atlas.

Response: Thank you!!!

Comment: In the results section: An inducible injury model to investigate systemic muscle repair, the authors write: "We treated 4dpf Tg(actc1b:NTR,2A,mCherry) larvae with a range of MTZ concentrations and incubation times and found 12hr treatment of 5mM MTZ drastically reduced swim function at 2dpi, but strikingly the injured larvae regained full swim function by 12dpi (Fig.1A,B)"

Previous use of NTR lines have shown in some cases various degrees of toxicity and a new improved NTR2.0 system was created (Sharrock et al., 2022, PMID: 35132245). It is not clear to me what NTR system is being used here. It is necessary to assess if only muscle fibres are damaged/ablated or

© 2026. Published by The Company of Biologists under the terms of the Creative Commons Attribution License (https://creativecommons.org/licenses/by/4.0/).

that other tissues are also affected under the experimental regime selected by the authors. This is especially important if the first-generation NTR system was used. (In that regard, pax7aGFP,2A,NTR2.0 may have a direct effect on the brain due to pax7 expression).

Response: Thank you for the comment. NTR1.0 was used to make actc1b:NTR-2A-mCherry and NTR2.0 was used for pax7aGFP-2A-NTR. The NTR versions for each line are now described in the Methods. Regarding toxicity, the NTR1.0 protein is not itself toxic but the high doses of MTZ (10uM for >24hrs) needed to ablate some cell types can be toxic (PMID: 35132245). The NTR2.0 is more efficient and circumvents the need for these high doses. However, we used a moderate MTZ dose for our experiments (5uM for 12hr), which should not be toxic. Although there is no evidence for a bystander effect for NTR1.0 (PMID: 35132245), we checked the overall morphology of the nervous system, the vascular system, and the heart in Tg(actc1b:NTR-2A- mCherry) larvae two days after MTZ treatment (revised Fig. S1B-D). We did not observe obvious changes in tissue morphology. While checking every cell type in great detail is not feasible, our studies suggest the actc1b:NTR-2A-mCherry injury model specifically targets skeletal muscle.

Regarding pax7a in the nervous system, the survival curve in Fig S1F shows treating pax7aGFP-2A-NTR fish with MTZ does not affect survival in the absence of muscle injury (light blue line in survival curve). Our interpretation is that Pax7a expressing cells are necessary for survival after muscle injury.

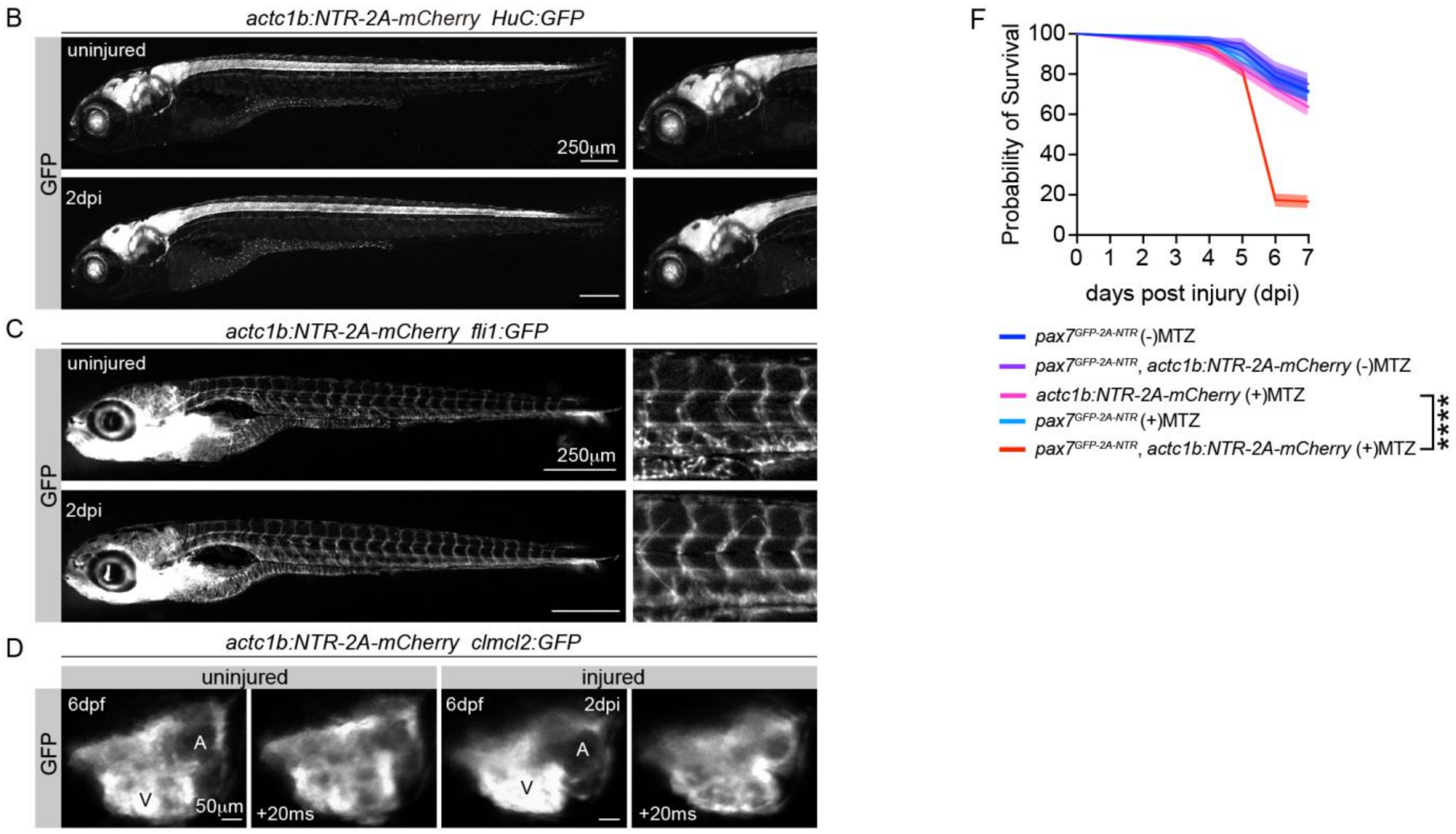

Comment: The damage to the muscle is described very briefly and should be elaborated further; what happens to the sarcomeres? MyHC only shown as a cross section (Figs.1D and 5D for example). A high magnification image of fibres with MyHC and/or other sarcomeric proteins in a lateral view would be beneficial to understand the cellular events in the systemic injury.

Response: Agreed. Our initial analysis was a bit thin. We have made two changes to address potential sarcomere disassembly. First, we provide high magnification images of slow muscles from injured and uninjured *smyhc1*^{sfgfp} larvae (Fig. S1A, see below). These images show myofibrils were highly disorganized in the injured myofibers and often lacked striations. Second, we used longitudinal sections of injured and uninjured larvae to label fast muscles with phalloidin, and found injured fast fibers lacked striations (Fig. 1G, see below). Since the resolution of these micrographs is not sufficient to visualize sarcomere structure we revised our conclusion, which now states systemic injury alters myofiber morphology and disrupts the contractile machinery in both slow and fast myofibers

© 2026. Published by The Company of Biologists under the terms of the Creative Commons Attribution License (https://creativecommons.org/licenses/by/4.0/).

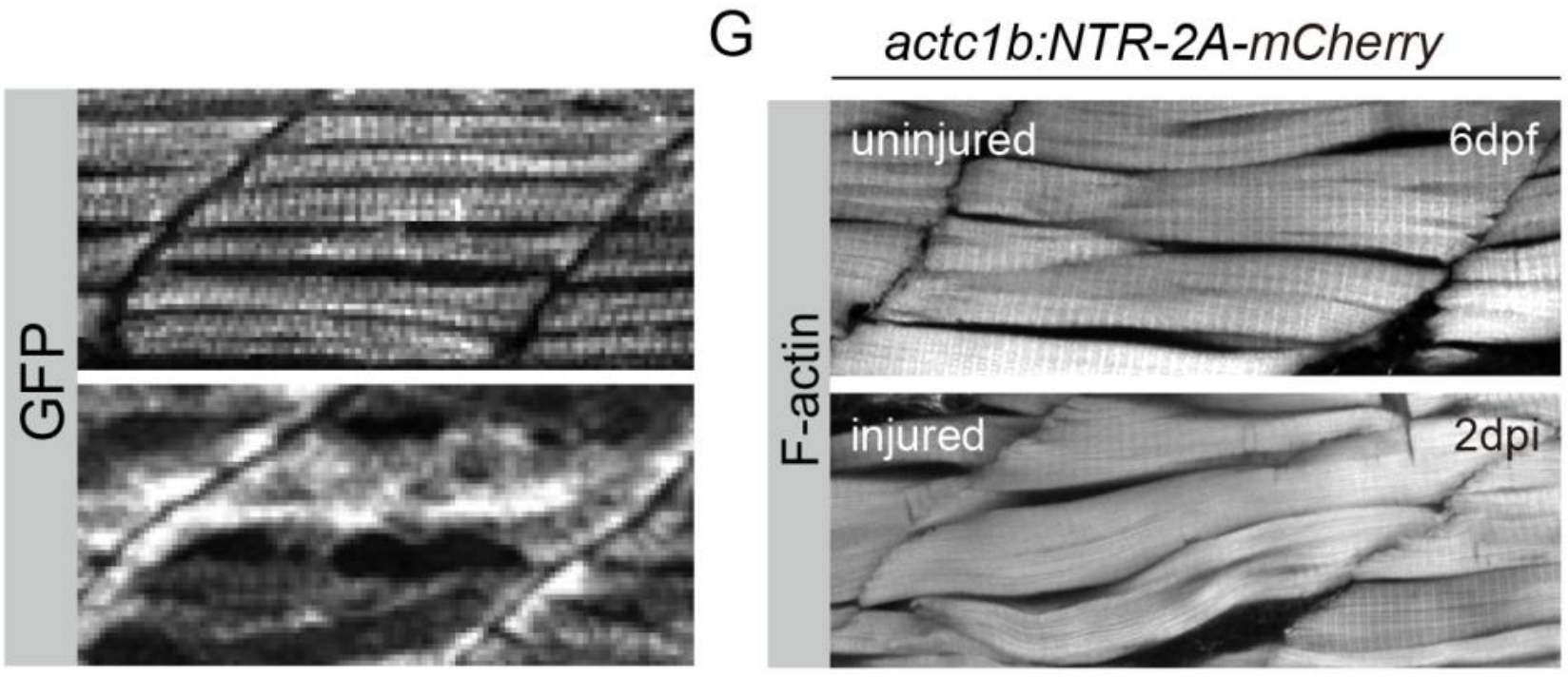

Comment: In addition to point 2, in the results section "Systemic regeneration repairs surviving myofibers", the authors claim that "Systemic repair therefore does not create new myofibers" and that "The absence of smyhc1sfgfp expression in fast myofibers during systemic repair and the persistence of GFP expression in lineage traced myofibers after injury argue the primary cellular mechanism of systemic muscle repair is fusion-based repair of surviving myofibers.". What happens then during the systemic injury to muscle nuclei?,is there any nuclear apoptosis and fusion? If not is the only change is in the MuSC state? The authors say : "Injured larvae transgenic for Tg(actc1b:NTR,2A,mCherry) and Tg(myf5:GFP) activated GFP expression at 2dpi, suggesting systemic repair is a MuSC-based process (Fig. 1E)." The regeneration however, could also be re-activation of muscle genes like myf5 in the existing myofiber nuclei and not in the MuSC. The manuscript needs some insights into these questions and the authors should rephrase their strong conclusions throughout unless they provide enough evidence to support fusion-based system. This can be done for example by use of DAPI stain, nuclear transgenics or other reagents and by looking into upregulation of fusion genes such as myomixer or jamb/jamc etc.).

Response: Thank you for these great suggestions. Visualizing fusion-based repair is indeed difficult. We live imaged Tg(actc1b:NTR,2A,mCherry) and Tg(myf5:GFP) larvae immediately after injury and found evidence for fusion events (Movie 1) that recapitulate previous reports of pax7a:GFP fusion (Pipalia Dis Model Mech 2016). DAPI did not penetrate to the myotome in live animals, but we were able to identify a significant increase in centralized nuclei at 1- and 2dpi, further supporting fusion-based repair (Fig. 1F). We used cleaved Caspase 3 antibody to visualize apoptosis, which showed minimal reactivity in myofibers after systemic injury (Fig. S2A). Further assessment of nuclear apoptosis with TUNEL was therefore not warranted. Myomixer and Jam2a (jamb) mRNA were similarly enriched in activated MuSC populations in response to systemic and local injury (Fig. 5F, see below). These data argue systemic and local injuries activate the fusogenic machinery in MuSCs to a similar extent.

F

		systemic (2dpi)	local (2dpi)
MuSC, act #1			
	mymk	241.59	32.72
	jam2a	543.98	350.02
MuSC, act #2			
	mymk	0.01	2.49
	jam2a	83.76	19.86

*normalized expression is shown for each transcript

Minor points

Comment:. The authors say: "slow myosin heavy chain 1 (smyhc1) is expressed in newly created fast myofibers after local muscle injury in larvae (Gurevich et al., 2016) and say " Systemic repair therefore does not create new myofibers."The authors quote Gurevic et al. 2016 that used the Tg (smyhc1:gfp) but use a novel knock-in line here. Does the knock-in show the same effect of transient activation in fast cells after local injury?

© 2026. Published by The Company of Biologists under the terms of the Creative Commons Attribution License (https://creativecommons.org/licenses/by/4.0/).

Response: Great idea. Yes!! The smyhc1^{gfp} knock-in behaves like the smyhc1-GFP transgene in response to local injury. We can clearly see GFP+ myofibers in the fast myotome after needlestick injury (Fig. S2D; see below).

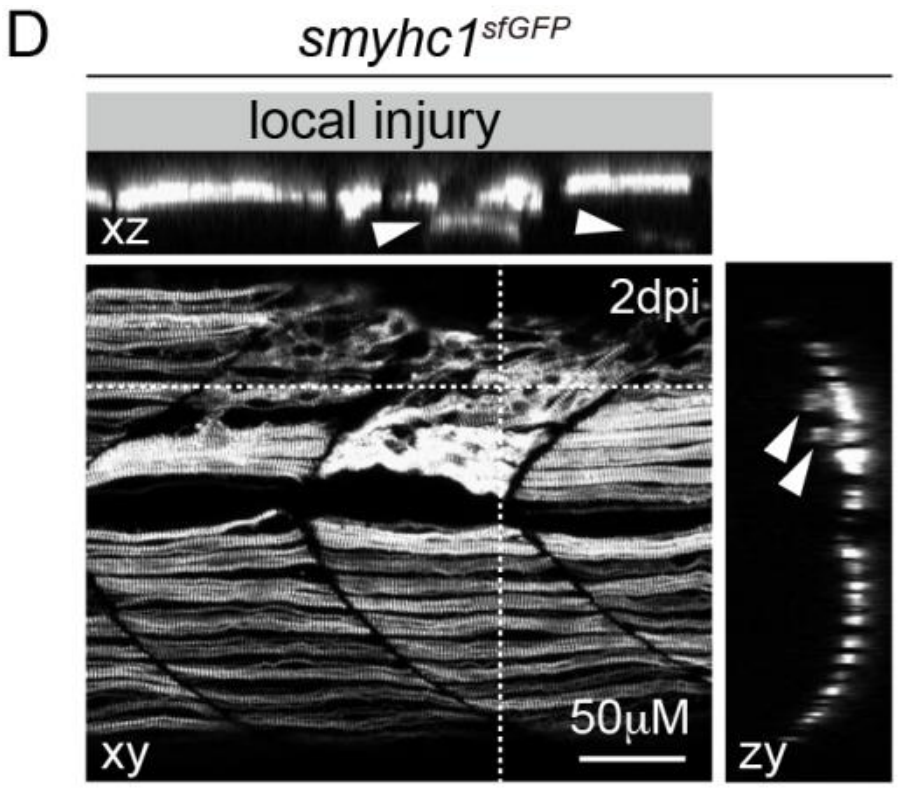

Comment:. In Fig1F, actc1b:NTR,2A, mCherry shown only with Mtz. Where is 'no Mtz' control and how did the statistics done for this pair then?

Response: We didn't observe any lethality in Tg(actc1b:NTR-2A-mCherry) fish without MTZ and chose to use the more stringent control with pax7a$^{NTR-2A-GFP}$, Tg(actc1b:NTR-2A-mCherry) double transgenic fish. We added additional statistical analyses.

Comment: "Uninjured smyhc1sfgfp larvae expressed GFP in the superficial slow myofibers of the truck musculature but not in the deep fast myofibers (Fig.S1A,B)." Should say trunk

Response: Nice catch. Thanks!!

Comment: Figure S6. Hb-EGF does not En1+ MuSCs or local muscle repair -nonsensical header. Please correct.

Response: Very nonsensical. Revised.

Comment: In the Discussion: "Hb-EGF is produced by both macrophages and endothelial in the liver after ischemia injury and is required for hepatocyte proliferation during liver regeneration (Wu et al., 2024; Yang et al., 2024)."-Should be 'endothelial cells'

Response: Thanks for the critical read of our text. Hopefully we have fixed all of these typos…

Reviewer 3

Comment: The manuscript by Dean, Saraswathy and colleagues describes development of a new nitroreductase-based systemic skeletal muscle injury model and extensive comparison of this new model to the "standard" transection. They identify Hb-EGF signaling as uniquely induced in response to systemic muscle injury and necessary for repair after systemic injury. Along the way, authors generated and characterized five new transgenic lines: three by transposon transgenesis and two by knock-in.

Response: Thank you for recognizing our hard work!!!

Comment: In multiple places throughout the manuscript authors argue that the differences in response are primarily attributable to the scale of the injury. This may very well be the case. However, there is an alternative explanation: different modes of injury - targeted death of cardiomyocytes versus physical injury inevitably immediately affecting many different cell types - are likely to induce at least somewhat different responses. There does not seem to be a straightforward way to unequivocally rule this alternative explanation out. This has to be clearly acknowledged.

© 2026. Published by The Company of Biologists under the terms of the Creative Commons Attribution License (https://creativecommons.org/licenses/by/4.0/).

Response: Thank you for the insightful comment. We agree the type of cell damage is also distinct between the models, and we have added the following paragraph to the Discussion:

One limitation to our study is that systemic injury damaged myofibers that were subsequently repaired while local injury caused myofiber apoptosis. It is possible that the differential transcriptional responses we observed between the injury models is due to the type of myofiber damage and not the scale of the injury. While the type of myofiber injury likely determines some aspect of the regenerative response, systemic injury induced more MuSC activation and the activated MuSCs themselves were transcriptionally similar to the activated MuSCs induced by local injury (Fig. 5E,F). These results argue the MuSC response to each injury is similar, but the magnitude of the response is dictated by the scale of the injury. An NTR-MTZ platform with temporal and spatial control of injury would enable comparative studies of the regenerative responses to NTR-MTZ induced myofiber damage verses needlestick-induced myofiber cell death in a localized region of the myotome. This model could provide new insights into the mechanisms by which muscle diseases associated repeated myofiber damage and repair, like muscular dystrophies, activate or disrupt the regenerative response.

Minor concerns:

Comment: Please Clarify if generated and used Tg(actc1b:NTR,2A,mCherry), Tg(actc1b:NTR,2A) and Tg(actc1b:CreERT2) were single-copy by Mendelian segregation, or if multicopy fish were used. It appears that in each case, progeny of two independent founders were used. It would be good to let the readers know which founder was used for which experiments. Authors make it clear that the main issue when generating Tg(actc1b:CreERT2) line(s) was low recombination activity. Was expression of Tg(actc1b:NTR,2A,mCherry) and Tg(actc1b:NTR,2A) reproducible between founders, or was it highly subject to position effects?

Response: We apologize if this was not clear. The specific comments regarding lines were addressed in a revised paragraph in the Methods (see below). We didn't have trouble with recombination activity of the CreERT2 line, but maybe the previous version of Fig. 2 didn't make that clear. The revised figure shows most myofibers are lineage trace labelled.

For all transgenic lines, two founders with the most consistent levels of expression and activity were maintained and outcrossed to wild-type (Tu). For lines that lack a fluorescent marker, the F1 generation was in crossed to make multicopy lines that were validated by outcrossing and assessing Mendelian segregation. Lines with fluorescent markers were maintained as single copy. Lines derived from independent founders behaved similarly with respect to expression and activity, arguing our selection strategy minimized position effects between insertions. For each transgene, a single founder line was used for downstream experiments.

Comment: Please provide a reference for the reporter Tg(ubb3:RFP.loxP.STOP.loxP.GFP) lineage tracing reporter (page 7, paragraph 3). The Mosimann et al 2011 paper cited for Tg(ubb:LOXP-RFP-STOP-LOXP-GFP) reporter on page 16 only describes ubiquitous green-to- red reporter Tg(ubi:loxP-GFP-loxP-mCherry). Please also reconcile if Tg(ubb3:RFP.loxP.STOP.loxP.GFP) (on page 7 and Tg(ubb:LOXP-RFP-STOP-LOXP-GFP) on page 16 describe the same construct.

Response: Wow. The reviewers certainly knows these lines well. The correct reference has been added.

Second decision letter

MS ID#: dev.204993R1

MS TITLE: Hb-EGF directs systemic muscle repair

© 2026. Published by The Company of Biologists under the terms of the Creative Commons Attribution License (https://creativecommons.org/licenses/by/4.0/).

AUTHORS: Haley C. Dean, Vishnu Saraswathy, Amulya Saini, Sibani Nachadalingam, Jennifer McAdow, Amruta Tendolkar, Mayssa H. Mokalled and Aaron Johnson

Dear Dr Johnson, dear Aaron,

I have now received all the referees reports on the above manuscript, and have reached a decision. The referees' comments are appended below.

The overall evaluation is positive and we would like to publish a revised manuscript in Development, provided that the Referee comments can be satisfactorily addressed. Please attend to all of the reviewers' comments in your revised manuscript and detail them in your point-by-point response. If you do not agree with any of their criticisms or suggestions explain clearly why this is so. If it would be helpful, you are welcome to contact us to discuss your revision in greater detail. Please send us a point-by-point response indicating your plans for addressing the referees' comments, and we will look over this and provide further guidance.

Reviewer 1

Advance summary and potential significance to field

The authors have addressed most of my concerns, and the manuscript is now much improved. Congratulations to the authors on this important work. I have only a few minor comments listed below.

Fig 1 legend: "Straited F-actin" should be corrected to "Striated F-actin"?

"… we knocked an NTR-2A-GFP cassette into the pax7a locus to generate pax7aGFP-2A-NTR fish" should read "GFP-2A-NTR cassette".

Fig S2D: The label "50μM" should be corrected to "50μm".

Fig 2D: Does nRFP mean nuclear RFP? But the genotype is listed as H2B-mCherry. What does cGFP indicate, cytoplasmic GFP? This needs to be clarified. The figure legend reads "ubb:LOXP-H2BmCherry-STOP-LOXP-SFGFP", whereas the images labels say GFP. I suggest using consistent labeling.

Fig 5B: It would be helpful to show the green channel of frem2a staining separately.

Fig S6: The legend does not match the figure panels.

Fig S6A: With the current layout, it is difficult to determine coexpression or lack thereof. It would be helpful to show some merged channels, for example, GFP and Pax7, RFP and Pax7, as well as GFP and RFP.

Fig 6D: pax7a is expressed in many cell types in addition to MuSCs. In particular, the pax7a□° cells in the head after Hb-EGF injections (arrowheads) are unlikely to be MuSCs. The authors need to show and quantify MuSCs in the trunk.

Movie 1: The cell fusions shown are not very clear.

Reviewer 2

Advance summary and potential significance to field

I am happy with the response of the authors and the extra work made. The changes made to the manuscript have strengthen it, and I can now recommend it for publication in Development.

© 2026. Published by The Company of Biologists under the terms of the Creative Commons Attribution License (https://creativecommons.org/licenses/by/4.0/).

Reviewer 3

Advance summary and potential significance to field

No further comments.

Comments for the author

No further comments.

Second revision

Author response to reviewers' comments

Reviewer 1

Comment: The authors have addressed most of my concerns, and the manuscript is now much improved. Congratulations to the authors on this important work. I have only a few minor comments listed below.

Response: Thank you for the kind words. We greatly appreciate the attention you have given to helping us improve the manuscript.

Comment: Fig 1 legend: "Straited F-actin" should be corrected to "Striated F-actin"?

Response: Fixed!

Comment: "… we knocked an NTR-2A-GFP cassette into the pax7a locus to generate pax7aGFP-2A-NTR fish" should read "GFP-2A-NTR cassette".

Response: Fixed!

Comment: Fig S2D: The label "50μM" should be corrected to "50μm".

Response: Fixed!

Comment: Fig 2D: Does nRFP mean nuclear RFP? But the genotype is listed as H2B- mCherry. What does cGFP indicate, cytoplasmic GFP? This needs to be clarified. The figure legend reads "ubb:LOXP-H2BmCherry-STOP-LOXP-SFGFP", whereas the images labels say GFP. I suggest using consistent labeling.

Response: Thanks for catching this. We relabeled the panel "cyto-sfGFP/H2B- mCherry/DAPI" and clarified the sfGFP is cytoplasmic in the figure legend.

Comment: Fig 5B: It would be helpful to show the green channel of frem2a staining separately.

Response: Great idea. Done.

Comment: Fig S6: The legend does not match the figure panels.

Response: Yikes. Fixed.

Comment: Fig S6A: With the current layout, it is difficult to determine coexpression or lack thereof. It would be helpful to show some merged channels, for example, GFP and Pax7, RFP and

© 2026. Published by The Company of Biologists under the terms of the Creative Commons Attribution License (https://creativecommons.org/licenses/by/4.0/).

Pax7, as well as GFP and RFP.

Response: Agreed. We made the recommended changes which added clarity but did not change our conclusions. En1 and Pax7 are largely co-expressed but there are instances were En1 positive cells lacked Pax7. Neither En1 nor Pax7 are expressed in Smyhc1+ slow fibers.

Comment: Fig 6D: pax7a is expressed in many cell types in addition to MuSCs. In particular, the pax7a^{+} cells in the head after Hb-EGF injections (arrowheads) are unlikely to be MuSCs. The authors need to show and quantify MuSCs in the trunk.

Response: We quantified the GFP+ cells as requested. The number of GFP+ cells in the trunk was significant at one concentration of Hb-EGF. While the reviewer makes a good point about pax7a expression outside MuSCs, the head contains quite a lot of skeletal muscle (Figs. 1C and 2D), and the micrographs used to quantify GFP+ cells-which are also used in Fig. 6D-are from an epifluorescence microscope with low tissue penetrance. The GFP+ cells are superficial and in close proximity to skeletal muscle. In addition, the injected protein itself may not be equally distributed, as Dextran itself remains localized anteriorly near the site of injection (Fig. S6D).

Comment: Movie 1: The cell fusions shown are not very clear.

Response: Unfortunately, these were the best images we could make with the available tools.

Reviewer 2: I am happy with the response of the authors and the extra work made. The changes made to the manuscript have strengthen it, and I can now recommend it for publication in Development.

Response: Thank you!!!

Reviewer 3: SUMMARY OF THE ADVANCE MADE IN THIS PAPER AND ITS POTENTIAL SIGNIFICANCE TO THE FIELD

No further comments.

Response: Thanks again to all the reviewers for the great insights to improve our manuscript.

Third decision letter

MS ID#: dev.204993R2

MS TITLE: Hb-EGF directs systemic muscle repair

AUTHORS: Haley C. Dean, Vishnu Saraswathy, Amulya Saini, Sibani Nachadalingam, Jennifer McAdow, Amruta Tendolkar, Mayssa H. Mokalled and Aaron Johnson

Dear Dr Johnson,

I am happy to tell you that your manuscript has been accepted for publication in Development, pending our standard publication integrity checks.

© 2026. Published by The Company of Biologists under the terms of the Creative Commons Attribution License (https://creativecommons.org/licenses/by/4.0/).
